# Progeria‐based vascular model identifies networks associated with cardiovascular aging and disease

**DOI:** 10.1111/acel.14150

**Published:** 2024-04-04

**Authors:** Mzwanele Ngubo, Zhaoyi Chen, Darin McDonald, Rana Karimpour, Amit Shrestha, Julien Yockell‐Lelièvre, Aurélie Laurent, Ojong Tabi Ojong Besong, Eve C. Tsai, F. Jeffrey Dilworth, Michael J. Hendzel, William L. Stanford

**Affiliations:** ^1^ The Sprott Centre for Stem Cell Research Ottawa Hospital Research Institute Ottawa Ontario Canada; ^2^ Ottawa Institute of Systems Biology Ottawa Ontario Canada; ^3^ Department of Cellular and Molecular Medicine University of Ottawa Ottawa Ontario Canada; ^4^ Cross Cancer Institute and the Department of Experimental Oncology, Faculty of Medicine and Dentistry University of Alberta Edmonton Alberta Canada; ^5^ Université de Strasbourg Strasbourg France; ^6^ School of Bioscience University of Skövde Skövde Sweden; ^7^ Division of Neurosurgery, Department of Surgery, Faculty of Medicine University of Ottawa Ottawa Ontario Canada; ^8^ Department of Cell and Regenerative Biology University of Wisconsin‐Madison Madison Wisconsin USA; ^9^ Department of Biochemistry, Microbiology & Immunology University of Ottawa Ottawa Ontario Canada

**Keywords:** aging, coronary artery, DNA damage repair, H4K16 acetylation, induced pluripotent stem cells, Lamin A, progeria, reprogramming, vascular smooth muscle cells

## Abstract

Hutchinson‐Gilford Progeria syndrome (HGPS) is a lethal premature aging disorder caused by a de novo heterozygous mutation that leads to the accumulation of a splicing isoform of Lamin A termed progerin. Progerin expression deregulates the organization of the nuclear lamina and the epigenetic landscape. Progerin has also been observed to accumulate at low levels during normal aging in cardiovascular cells of adults that do not carry genetic mutations linked with HGPS. Therefore, the molecular mechanisms that lead to vascular dysfunction in HGPS may also play a role in vascular aging‐associated diseases, such as myocardial infarction and stroke. Here, we show that HGPS patient‐derived vascular smooth muscle cells (VSMCs) recapitulate HGPS molecular hallmarks. Transcriptional profiling revealed cardiovascular disease remodeling and reactive oxidative stress response activation in HGPS VSMCs. Proteomic analyses identified abnormal acetylation programs in HGPS VSMC replication fork complexes, resulting in reduced H4K16 acetylation. Analysis of acetylation kinetics revealed both upregulation of K16 deacetylation and downregulation of K16 acetylation. This correlates with abnormal accumulation of error‐prone nonhomologous end joining (NHEJ) repair proteins on newly replicated chromatin. The knockdown of the histone acetyltransferase MOF recapitulates preferential engagement of NHEJ repair activity in control VSMCs. Additionally, we find that primary donor‐derived coronary artery vascular smooth muscle cells from aged individuals show similar defects to HGPS VSMCs, including loss of H4K16 acetylation. Altogether, we provide insight into the molecular mechanisms underlying vascular complications associated with HGPS patients and normative aging.

## Graphical abstract

We used a tractable model of vascular aging employing iPSC‐derived VSMCs from HGPS patients. This allowed us to track the progression of cellular aging phenotypes and begin to tease apart the molecular mechanisms behind these traits. Similar to HGPS VSMCs, we detected progerin accumulation, abnormal nuclei formation, induction of DNA damage as well as reduced H4K16ac in primary, aged CAVSMCs.

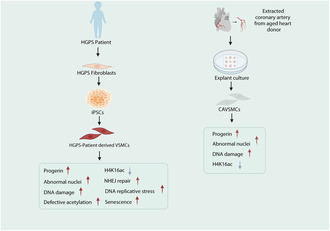

**Abbreviations**
53BP1: p53‐Binding Protein 1
8‐OxoG: 8‐oxo‐7,8‐dihydro‐2‐deoxyguanine
aniPOND: accelerated native isolation of proteins on nascent DNA
BDM: 2,3‐Butanedione monoxime
BRCA1L: breast cancer Type 1 susceptibility protein
BSA: bovine serum albumin
CAVSMC: coronary artery vascular smooth muscle cell
COL4A1: Collagen Type IV Alpha 1 Chain
DAPI: 4′,6‐Diamidino‐2‐Phenylindole
DMEM: Dulbecco's Modified Eagle Medium
DNA PKC: DNA‐dependent protein kinase catalytic subunit
DSB: double‐strand break
EB: embryoid body
ECM: extracellular matrix
FGF2: fibroblast growth factor 2
FITC: fluorescein isothiocyanate
GEO: gene expression omnibus
GO: gene ontology
H3K27me3: histone H3 lysine 27 trimethylation
H3K9me3: histone H3 lysine 9 trimethylation
H4K16ac: histone H4 lysine 16 acetylation
H4K20me3: histone H4 lysine 20 trimethylation
HAT: histone acetyltransferase
HDAC: histone deacetylase
HDACi: histone deacetylase inhibitor
HEPES: 4‐(2‐Hydroxyethyl)‐1‐piperazineethanesulfonic acid
HGPS: Hutchinson‐Gilford Progeria Syndrome
HR: homologous recombination
IgG: immunoglobulin G
iPSC: induced pluripotent stem cell
KEGG: kyoto encyclopedia of genes and genomes
LMNA: lamin A/C gene
MAPK: mitogen‐activated protein kinase
MEF: mouse embryonic fibroblast
MOF: histone acetyltransferase MOF
MRE11: meiotic recombination 11 Homolog A
MTA2: metastasis‐associated protein 2
NAC: N‐acetyl cysteine
NAT10: N‐acetyltransferase 10
NHEJ: non‐homologous end joining
PARP1: poly(ADP‐ribose) polymerase 1
PBS: phosphate‐buffered saline
PCNA: proliferating cell nuclear antigen
PFA: paraformaldehyde
PLA: proximity ligation assay
pRPA: phosphorylated replication protein A
RNA‐seq: RNA sequencing
ROS: reactive oxygen species
SA: senescence‐associated
SA‐βgal: senescence‐activated β‐galactosidase
SDS: sodium dodecyl sulfate
SIRT1: Sirtuin 1
TBST: Tris‐buffered Saline/0.05% Tween 20
TGF‐β1: transforming growth factor beta 1
TSA: trichostatin A
VSMC: vascular smooth muscle cell
XPA: xeroderma pigmentosum complementation Group A
Zmpste24: Zinc Metallopeptidase Ste24
βFGF: basic fibroblast growth factor

## 1 INTRODUCTION

Vascular diseases, such as heart attack and stroke, are ranked number one in morbidity and mortality in the developed world (Virani et al., 2020). Age is the primary risk factor driving cardiovascular disease (O'Donnell & Nabel, 2008; Wolf, 2012). The perturbation in the homeostasis of vascular smooth muscle cells (VSMCs) is a critical component underlying vascular calcification and atherogenesis during aging, whereby the vasculature undergoes significant remodeling in response to increased reactive oxygen species (ROS) and inflammatory cytokine secretion (Gorenne et al., 2006). This phenomenon ultimately leads to the formation of atherosclerotic lesions, driven in part by VSMC proliferation and migration to damaged sites in arterial walls (He & Zuo, 2015). An improved understanding of the molecular basis of vascular aging could identify novel therapeutic targets to develop effective prevention and treatment for cardiovascular and neurovascular diseases.

Accelerated aging syndromes encode dominant genetic determinants that could provide mechanistic insight into the molecular underpinnings of normative aging (Merideth et al., 2008). The most severe progeroid syndrome is Hutchinson‐Gilford Progeria Syndrome (HGPS), a premature aging disorder that occurs in 1 in every 4 million births worldwide (Hennekam, 2006). Children with HGPS develop severe osteoarthritis, alopecia, hearing loss, lipodystrophy, and other age‐related symptoms (Merideth et al., 2008). However, it is the deterioration of VSMCs that leads to terminal atherosclerosis in HGPS, which ultimately causes fatal cardiovascular complications such as heart attacks or stroke in their teenage years (Hennekam, 2006; Merideth et al., 2008). HGPS is caused by a single‐point mutation in *LMNA* (C1824T) that activates a cryptic splice site, leading to an internal 50 amino acid deletion which affects Lamin A maturation (De Sandre‐Giovannoli et al., 2003; Eriksson et al., 2003). Designated as “progerin”, the mutant Lamin A protein remains farnesylated and anchored to the nuclear membrane. Progerin expression in the normal aged population has been reported, suggesting that the cryptic splice site is functional in the absence of the C1824T mutation, although utilized at a much lower frequency (McClintock et al., 2007; Scaffidi & Misteli, 2006). Thus, it has been suggested that accumulation of progerin and other aberrant splice forms of Lamin A contribute to normative aging (Ashapkin et al., 2019; McClintock et al., 2007; Ragnauth et al., 2010; Scaffidi & Misteli, 2006).

One of the consequences of progerin accumulation is the alteration of the epigenetic landscape, which affects gene expression and chromatin dynamics. The loss of repressive marks associated with transcriptional repression, trimethylation of histone H3 at lysines 9 and 27, have been shown to decline in most (Chojnowski et al., 2020; Chojnowski et al., 2015; Shumaker et al., 2006; Zhang et al., 2016) but not all (Liu, Drozdov, et al., 2013; Liu, Wang, et al., 2013) studies. This correlates with a loss of peripheral heterochromatin (reviewed in (Dechat et al., 2008)). The acetylation of histone H4 on lysine 16, a key epigenetic mark that regulates chromatin accessibility (Shogren‐Knaak et al., 2006; Zhang et al., 2017), has been shown to be dysregulated in HGPS. However, the exact mechanism and specific role of histone acetylation at lysine 16 of histone H4 (H4K16), in HGPS and normative aging is not well understood. Some studies have reported increased levels of histone H4K16 acetylation in HGPS fibroblasts (Mattioli et al., 2018; Maynard et al., 2022), while others have shown decreased levels of H4K16 acetylation in the Zmpste24 mouse model of HGPS (Krishnan et al., 2011). Additionally, decreased H4K16 acetylation and changes in histone acetylation have been linked to normative aging (Krishnan et al., 2011; Peleg et al., 2016). Furthermore, aged hematopoietic stem cells have been correlated with reduced H4K16ac and loss of polar distribution of H4K16ac (Florian et al., 2012; Grigoryan et al., 2018).

To elucidate the initiation and progression of HGPS phenotypes, we previously generated a resource of HGPS and control induced pluripotent stem cells (iPSCs) (Chen, Chang, et al., 2017; Chen, Yang, et al., 2017). Here, we differentiated the iPSCs into VSMCs which were passaged in culture to examine cellular mechanisms of VSMC aging. Reminiscent of what has been observed in vascular disease and atherosclerotic plaques (Cervelli et al., 2012; Shah et al., 2018; Wang & Bennett, 2012), HGPS VSMCs exhibit increased ROS, DNA damage and cellular senescence. Unbiased proteomic analysis of replication forks identified altered usage of DNA repair pathways and histone acetylation. The unusual accumulation of 53BP1, which is displaced in S‐phase by the acetylation of histone H4K16 and is a key regulator of nonhomologous end joining (NHEJ), suggested that this acetylation may be compromised. We demonstrate that aging‐associated loss of histone acetyltransferase (HAT) activity and loss of histone H4K16 acetylation, required to displace 53BP1 during S‐phase, lead to increased engagement of the error‐prone double‐strand break (DSB) repair pathway. A similar loss of histone H4K16 acetylation has been reported in Fanconi anemia and also results in the accumulation of 53BP1 at sites of replication stress‐induced DNA damage (Renaud et al., 2016). Notably, we found that the acetylation rate is significantly reduced in HGPS VSMCs while the deacetylation reaction is increased. The net reduction in histone H4K16 acetylation provides a mechanism to explain the observed increased engagement of the NHEJ repair pathway in progerin‐expressing cells. Inhibition of the histone acetyltransferase MOF (KAT8, MYST1) in control VSMCs phenocopied various HGPS defects. Therefore, histone deacetylation (HDAC) inhibition offers the basis for a therapeutic target. Finally, we identified HGPS phenotypes, including nuclear morphology defects and progerin accumulation, in primary coronary artery VSMCs from aged individuals, supporting the use of HGPS as a model of vascular aging and disease.

## 2 MATERIALS AND METHODS

### 2.1 Patient samples and study approval

Ethics approval for all patient coronary samples used in this study was obtained from the Ottawa Health Science Network Research Ethics Board (Protocol ID 20150544‐01H). Coronary arteries were harvested from nontransplanted hearts of organ donors (*n* = 4) ranging in age from early 50s to late 60s. VSMCs were extracted from the medial layer of the coronary artery segments with modifications as previously described (Cornwell & Lincoln, 1989). Briefly, the samples were rinsed in 1x PBS or DMEM (Thermo Fisher) with Gentamicin (Wisent) and Antibiotic‐Antimycotic (LifeTech) three times, and one final time in isolation media [DMEM containing 25 mM HEPES pH 7.4, 1 mg/mL BSA (Wisent), Gentamicin and Antibiotic‐Antimycotic]. In a 10 cm petri dish, Peri‐adventitial fat was removed and the arteries were cut in the smallest possible pieces with a sterile blade and submerged with digestion media [isolation media supplemented with 200 units/mL collagenase type I (Thermo Fisher), 0.1 mg/mL elastase (Sigma) and 0.5 mg/mL soybean trypsin inhibitor (Thermo Fisher)]. The cells were collected in 6‐well plates coated with Matrigel in vascular smooth muscle cell media [Human Vascular Smooth Muscle Cell Basal Medium (Thermo Fisher) supplemented with Smooth Muscle Growth Supplement (Thermo Fisher), Antibiotic‐Antimycotic and Gentamicin].

### 2.2 Resources

All the iPSC lines used in this study were previously described (Chen, Chang, et al., 2017). The iPSC lines 0901C, 0031C, and AG1B generated from fibroblasts from the Progeria Research Foundation biobank are available via the Progeria Research Foundation through a Materials Transfer Agreement, while BJ1C and AG1B are available directly from the corresponding author (WLS). The RNA‐seq datasets are publicly available, deposited in the GEO database (GSE231761).

### 2.3 Human pluripotent stem cell culture

All iPSCs were maintained in Matrigel‐coated plates in E8 medium (DMEM/F12 supplemented with L‐ascorbic acid, sodium selenite, βFGF, TGF‐β1, sodium bicarbonate, holo‐transferrin, and Gentamicin) as previously described (Chen, Chang, et al., 2017).

### 2.4 VSMC differentiation

Differentiation of iPSCs to VSMCs was conducted based on a previously described protocol (Xie et al., 2007) with slight modifications. Briefly, embryoid bodies (EBs) were generated from iPSCs by treatment with Collagenase IV (Life Technologies) for 30 min at 37°C, before being gently dislodged with a cell scraper. Dislodged cell clusters were then plated in low‐cluster plates (Corning) for 7–10 days in EB medium (DMEM/F12 supplemented with 20% Knockout Serum Replacement (Invitrogen), GlutaMax, β‐mercaptoethanol, non‐essential amino acids, and Gentamicin After EB formation, EBs were plated on 0.1% gelatin‐coated plates for 3–5 days. EB outgrowths were trypsinized and cells were replated on Matrigel in Medium 231 (with growth supplement). Cells were passaged when confluency reached 80%. To differentiate VSMCs, cells were plated on gelatin‐coated 6‐well plates and grown in VSMC differentiation medium (Medium 231, differentiation supplement, Life technologies) for 7 days and characterized at each passage.

Primary coronary artery VSMCs were grown in the VSMC media supplemented with Smooth Muscle Growth Supplement and Gentamicin on gelatin‐coated 6‐well plates. The media was changed daily until the cells reached confluency. Confluent cells were gently treated with trypsin and split at 1:3. The cells were characterized with ACTA2 (CBL171, Millipore) and Transgelin (ab14106, Abcam).

### 2.5 Contractility assay and Ca2^+^ influx

VSMC contractility was assessed through vascular constrictor, Angiotensin II (Sigma Aldrich) and contraction inhibitor, 2,3‐Butanedione monoxime (BDM) (Cell Biolabs) treatment. In brief, both control and HGPS VSMCs, and CAVSMCs were seeded on a Matrigel‐coated 96‐well plate. After cells reached 60%–80% confluency, the cells were exposed to 20 mM BDM for 30 min and 20 μM Angiotensin II, and digital phase‐contrast images were captured at 10 min intervals for up to 80 min post‐treatment using Perkin Elmer Operetta CLS. Collagen contraction assays were performed according to the manufacturer's instructions (CBA‐201 Cell Contraction Assay Kit from Cell Biolabs). Cells were suspended on a freshly prepared bovine type 1 collagen solution (chilled on ice) and seeded at a density of ~1 × 10^6^ cells/well in a 24‐well plate. The plate was then placed in a 37°C incubator for 1 h to allow for collagen gel polymerization. After polymerization, 1.0 mL of culture medium was added on top of the gel lattice and incubated for 48 h. The cells were then treated with 20 mM BDM and 20 μM Angiotensin II at the indicated concentrations. Once the contraction agonists and inhibitor were added, the gels were carefully detached from the sidewalls of each well by a sterile scalpel. Pictures of the collagen gels were recorded using Bio‐Rad Gel Doc XR+ and the quantification of gel disc area was carried out using ImageJ software.

To investigate intracellular Ca2^+^ dynamics in VSMCs, the Fluo‐4 AM Ca2^+^ fluorescent indicator (Invitrogen) was employed. Both normal and HGPS VSMCs were treated by directly adding a 2 μM concentration of Fluo‐4‐Ca2^+^ indicator to the culture medium, and the cells were then incubated for 30 min before placing the cells on the microscope. Imaging of Angiotensin II‐stimulated Ca2^+^ flux signals was performed using a Leica SP8 laser scanning confocal microscope using a 25X 0.95 N.A. water objective and a white light laser source was used to excite Fluo‐4 AM at 488 nm. The emission was collected between 492 nm and 659 nm. Angiotensin II was added immediately after the first image was acquired. Cells were maintained at 37°C using a heated stage incubator in the presence of 5% CO_2_. Cells were then imaged every 5 s for up to 5 min following the addition of Angiotensin II. For quantification, the background was subtracted, and a region of interest was placed around the individual cells measured. The intensity values were then collected for each time point. The data was then normalized by using the maximum value obtained in each cell type as 100. The value of the medium following background subtraction was defined as zero and the resulting fluorescence intensity was scaled between 0 and 100.

### 2.6 ROS measurement

ROS levels in cultured cells were measured using the Image‐iT™ LIVE Green Reactive Oxygen Species Detection Kit (I36007, Life Technologies). Briefly, cells were incubated with 25 μM carboxy‐H2 DCFDA diluted in warm HBSS (Life Technologies) for 30 min at 37°C in the dark. Hoechst was added at 1.0 μM final concentration to the solution 5 min prior to the end of the incubation period. For ROS induction, control cells were treated with TBHP at a concentration of 100 μM for 1 h at 37°C before the addition of 25 μM carboxy‐H2 DCFDA. Treated cells were washed twice with warm HBSS before being imaged using an epifluorescence microscope or through a high‐content imaging system for downstream analysis.

### 2.7 Drug treatment

Experiments were performed on P14 cultures. For NAC treatment, cells were treated with NAC at 20 μM for 72 h before being harvested for further experiments. For HDAC inhibition, cells were incubated with 1.5 μM trichostatin A for 24 h prior to fixation or harvesting. Cells were irradiated with Cesium‐137 in ambient air at a dose rate of 1 Gy per minute for 3 min. Cells were then incubated at 37°C until fixation or harvesting.

### 2.8 H4K16ac turnover kinetics

#### 2.8.1 Acetylation rate

24 h after seeding, both HGPS and control VSMCs were treated with 250 nM Quisinostat (JNJ‐26481585), a histone deacetylase (HDAC) inhibitor. Cells were fixed with 4% paraformaldehyde (PFA) in 1x PBS pH 7.4 for 10 min at room temperature at various time points post‐treatment, including 0 min, 5 min, 10 min, 15 min, 30 min, 60 min, 90 min, and 120 min.

#### 2.8.2 Deacetylation rate

24 h after cell seeding, cells were treated with 150 nM Quisinostat for a duration of 4 h. Subsequently, cells were washed with 1x PBS pH 7.4, and fresh media was replaced. Following the media change, cells were fixed at 0 min, 5 min, 10 min, 15 min, 30 min, 60 min, 90 min, and 120 min with 4% PFA pH 7.4.

#### 2.8.3 Immunofluorescence staining

Following fixation, PFA was removed and 1x PBS was added. PBS was removed and cells were permeabilized by adding PBS 0.5% Triton X‐100 for at least 10 min. Cells were rinsed three times with 1x PBS and then incubated with H4K16ac rabbit polyclonal antibody obtained from Sigma Aldrich (07–329) as the primary antibody by placing the coverslip side down on a 40 μl drop of antibody on Parafilm, avoiding air bubbles, for 45 min. Cells were rinsed once with 1× PBS with 0.1% Triton X‐100 and then rinsed three times again with 1x PBS and left in 1x PBS. Cells were incubated with Alexa Fluor® 488‐Goat Anti‐Rabbit IgG as the secondary antibody by placing the coverslip side down on a 40 μl drop of antibody on a Parafilm for 45 min. After 45 min, cells were rinsed once with 1x PBS with 0.1% Triton X‐100 and three times with 1× PBS. Cells were then incubated with DAPI staining solution for 20 min. Coverslips were then mounted side down onto slides with Mowiol mounting media from Millipore (475904‐M). Immunofluorescence images were acquired using an Upright Zeiss AxioImager epifluorescence microscope equipped with a Photometrics PRIME BSI camera using a 40 × 1.4 N.A. Oil immersion objective.

#### 2.8.4 Western blotting

HGPS and Control VSMCs were treated with 1.5 μM TSA for 24 h. VSMCs then were washed in ice‐cold sterile 1x PBS, centrifuged at 2000 *g* for 10 min at 4°C, the supernatant aspirated and the cells frozen at −80°C. The cell pellet was thawed on ice before adding 200 μL ice‐cold 2x SDS lysis buffer (2% SDS, 50 mM Tris pH 7.5, 10% glycerol, protease inhibitor cocktail) and the sample was sonicated for 1 min with a Model 705 Sonic Dismembrator (Fisher Scientific) to generate the cell extract. 2x SDS sample loading buffer (0.195 M Tris pH 6.8, 0.2% bromophenol blue, 6% sodium dodecyl sulfate, 30% Glycerol, 3% β‐mercaptoethanol) was added to 20 μg of the lysed sample. The sample was denatured by heating at 95°C for 10 min using a preheated heat block, and then separated on 15% SDS‐PAGE gels at 100 V for 45 min. Nitrocellulose membrane (BIO‐RAD, 162–0112) was prepared according to the manufacturer's instructions and protein transferred at 30 mA at 4°C for 18 h. For immunoblotting, the transferred membrane was rinsed briefly in transfer buffer (20% methanol, 20% 10X PBS without SDS, 60% distilled water and then blocked in 5% non‐fat dry milk in 1x PBS for 1 h at room temperature. The membrane was rinsed three times in Tris‐Buffered Saline/0.05% Tween 20 (TBST) and incubated at 4°C overnight in the combined primary antibodies (mouse anti‐histone H3, (05–499) Sigma Aldrich) and rabbit anti‐acetyl histone H4K16 (Sigma Aldrich, 07–329) in TBST. The membrane was washed three times for 5 min in PBST prior to incubation with the secondary antibodies (IRDye® 800CW Goat anti‐Rabbit IgG and IRDye® 680RD Goat anti‐Mouse IgG, LI‐COR) for 2 h at room temperature. Following antibody incubation, the membrane was rinsed in TBST before being read on the Odyssey Fc Imaging System (LI‐COR Bioscience). Membranes were scanned on fluorescence, using 685 nm and 785 nm emission filters. For Western blot analysis, the resolution of the H3 and H4 bands enabled the export of the data as a single grayscale image. Background was subtracted from the image and the integrated intensity of each band was measured following segmentation using thresholding. The integrated intensity of the H4K16 acetylation band was divided by the integrated intensity of the H3 band to normalize for differences in protein loading. Subsequently, the value of the control histone H4K16 acetylation was set to 1.0 and the remaining sample values normalized to the value of the untreated control.

### 2.9 Immunofluorescence and histochemistry

Immunofluorescence analyses were performed as previously described (Chang et al., 2013). Briefly, cells were fixed in formalin for 15 min at room temperature and washed with 1x PBS. Samples were then permeabilized with 0.1% Triton X‐100 for 20 min, blocked for 1 h with 3% skim milk in 1x PBS, and primary antibodies were incubated for 2 h at room temperature or overnight at 4°C. Next day, samples were treated with secondary antibodies for 1 h, followed by Hoechst staining for 5 min being mounted for imaging using epifluorescence microscope, confocal microscope, or high‐content imaging analysis (Cellomics ArrayScan VTI). Unbiased quantification of fluorescent signals was performed on 1000–4000 cells using the Target activation, CellProfiler, or CellProfiler‐Analyst algorithms.

Tissues were fixed in 4% PFA and embedded in paraffin. Cross‐sections (5 μm) were stained with Hematoxylin/Eosin and Verhoeff‐van Gieson. Images were captured using a Meyer instrument PathScan Enabler, Zeiss Observer A1.

Primary antibodies used were Calponin (M3556, DAKO), ACTA2 (CBL171, Millipore), Transgelin (ab14106, Abcam), 53BP1 (4937S, New England Biolabs), γH2A.X (9718S, New England Biolabs), phospho‐RPA32 (S4/S8) (A300‐245A, BethylLabs), MOF (PLA0161, Sigma Aldrich), H4K16ac (07–329, Sigma Aldrich), H4K20me3 (PA5‐40089, Thermo Fisher Scientific), Pan BrdU (555,627, BD Biosciences), BRCA1 (sc‐6954, Santa Cruz), DNA PKC (MABC1236, Millipore), Progerin (05–1231, Sigma Aldrich), Lamin A/C (MAB3540, Sigma Aldrich), FITC‐avidin (A2050‐2ML, Sigma Aldrich), FGF2 (ab208687, Abcam), COL4A1 (AF6308‐SP, Bio‐Techne), Smoothelin (ab8969, Abcam), MYH11 (PA5‐82526, Thermo Fisher Scientific).

Cells that exhibited senescence‐associated β‐galactosidase (SA‐β‐gal) activity were detected by employing a β‐galactosidase staining kit (CBA‐232, Cell Biolabs) in accordance with the guidelines provided by the manufacturer.

### 2.10 AniPOND

Replication fork proteins were captured using the aniPOND technique as previously described (Leung et al., 2013). Briefly, 1 × 10^7^ cells from each line were labeled with 5‐ethynyl‐2′‐deoxyuridine (EdU) for 1 h before being treated with nuclear extraction buffer for 15 min to extract nuclei. Samples were then treated with biotin azide for 1 h to biotinylate nascently‐replicated chromatin through click reaction. Biotinylated chromatin was then sheared and extracted from nuclei through sonication before being purified from overnight streptavidin incubation.

Following chromatin extraction, replication fork proteins were identified through mass spectrometry that was performed at the Ottawa Hospital Research Institute Proteomics Core Facility (Ottawa, Canada). Proteins were digested in‐gel using trypsin (Promega). Peptide extracts were concentrated by Vacufuge (Eppendorf). LC–MS/MS was performed using a Dionex Ultimate 3000 RLSC nano HPLC (Thermo Scientific) and Orbitrap Fusion Lumos mass spectrometer (Thermo Scientific). MASCOT software version 2.6.2 (Matrix Science) was used to infer peptide and protein identities from the mass spectra. The observed spectra were matched against human sequences from SwissProt (version 2018–05) and also against an in‐house database of common contaminants. The results were exported to Scaffold (Proteome Software) for further validation and viewing.

Differentially abundant proteins were identified based on Fisher's exact test (*p* < 0.05) between control and HGPS proteins. Protein networks were identified using the STRING protein database, and significant protein complexes were analyzed on the basis of gene ontology (GO) using Metascape (Zhou et al., 2019).

### 2.11 RNA‐seq

RNA was isolated using the Nucleospin RNA Kit (Macherey‐Nagel) following the manufacturer's instructions. High‐quality total RNA was determined using Bioanalyzer. Libraries were sequenced on a HiSeq 2500 (Illumina) using the HiSeq SBS V4 kit to generate 100‐bp single reads. HISAT2 was used to map the reads to the GRCh38 genome. Differential expression was quantified using DESeq2 and subjected to filtering and test corrections. Genes with a *q*‐value <0.05 were considered differentially expressed. Hierarchical clustering was calculated using Euclidian distance between normalized expression values for all transcripts. GO analyses were performed using the random sampling method of the GOseq package. Pathway enrichment analysis was performed using the KEGG pathway database. Different combinations of control and HGPS VSMCs were analyzed in each passage, and only terms and pathways that were common in at least 2 combinations were chosen.

### 2.12 Alkaline comet assay

The alkaline comet assay was performed as previously described (Maganti et al., 2018). In preparation for the comet assay, 1% low melting‐point agarose (LMA) was melted by being submerged in a beaker of boiling water with the cap loosened, followed by a cooling period of 20 min at 37°C. Cell samples were typsinized and combined with warm LMA at a ratio of 1:10 cells:LMA, and 120 μL of the mixture was immediately pipetted onto labelled Gelbond film strip (Lonza, #53740). Cells were applied to the hydrophilic side of the film into a circle of approximately 25 mm in diameter. Films with the gel were then placed flat in the dark at 4°C for 10 min until the gel solidified. The films are then immersed in prechilled lysis solution and incubated at 4°C for 45 min. Following lysis, the films were immersed into freshly made Alkaline Unwinding solution (6 g NaOH, 250 μL 200 mM EDTA in 1 L solution with dH_2_O) for 45 min in the dark. The films were then transferred to a horizontal electrophoresis apparatus where the films are placed equidistant from each electrode in alkaline electrophoresis solution (12 g NaOH, 1 mM EDTA pH 8 in 1 L solution with dH_2_O), and electrophoresis was performed at 30 V for 30 min at constant amperage of 300 mA. During electrophoresis, temperature fluctuations were minimized by placing the apparatus in a walk‐in refrigerator. Following electrophoresis, films were dried in a desiccated container overnight at room temperature. The next day, the slides were stained by submerging in SYBR Green stain at 1:10000 dilution in TE buffer pH 7 for 5 min. The films were then air‐dried and mounted with coverslips for visualization by fluorescence microscopy using the GFP channel. The Olive tail moments were analyzed using the OpenComet Plugin in ImageJ.

### 2.13 qPCR

Quantitative PCR was performed as previously described (Walker et al., 2007). Serial dilution of qPCR primers that were used in this report have been previously reported to detect Progerin mRNA transcripts (Scaffidi & Misteli, 2006).

### 2.14 Proximity ligation assay (PLA)

After cell culture reached 80% confluency, cells were washed twice in PBS and fixed in 3.7% PFA for 10 min at room temperature. Fixed cells were subsequently permeabilized in 0.1% Triton X‐100 (Sigma Aldrich) for 20 min and blocked with Duolink Blocking reagent (Sigma Aldrich) for 1 hour at room temperature. Cells were incubated with primary antibodies diluted in Duolink antibody diluent (Sigma Aldrich) at 4°C overnight. The next day, samples were washed 3 times in 1x PBS, followed by incubation with Duolink In Situ PLA Probe Anti‐Mouse PLUS and Anti‐Rabbit MINUS (Sigma Aldrich) for 1 hour at 37°C in a humidity chamber. After incubation, cells were washed 3 times for 10 min in Buffer A (Sigma Aldrich) and incubated with DNA ligase in diluted ligase buffer (Sigma Aldrich) for 30 min at 37°C in a humidity chamber. Subsequently, cells were washed again 3 times for 10 min each in Buffer A and incubated with DNA polymerase diluted in polymerase buffer with far red fluorescence‐labelled oligonucleotides (Sigma Aldrich) for 90 min in a humidity chamber, before being washed and mounted in DAPI mounting medium (Duolink, Sigma Aldrich) for imaging and foci analysis.

### 2.15 DsiRNA knockdown

To knockdown MOF, DsiRNA transfection was performed using the RNAiMAX transfection protocol (Life Technologies). DsiRNAs for MOF were purchased from IDT. Cells were plated until reaching 80% confluency before being transfected with DsiRNAs using RNAiMAX (Life Technologies) and its suggested dilution format. Expression of MOF was tested using qPCR 3 days post‐transfection.

### 2.16 Statistical analysis

Data and statistical analyses were performed using Excel and GraphPad Prism software. Results are shown as mean ± SEM/SD, unless otherwise stated. Differences were considered significant at *p* < 0.05 (*p < 0.05, **p < 0.01, ***p < 0.001), and differences where significance is not shown are considered non‐significant, unless otherwise stated. The significance of intergroup differences was assessed via one‐way or two‐way ANOVA followed by Tukey's multiple comparison test, two‐tailed Student's *t* test for two‐component comparisons after a normal distribution was confirmed and Pearson correlation coefficients, as appropriate.

## 3 RESULTS

### 3.1 Passaged HGPS patient‐derived VSMCs exhibit hallmark HGPS defects

**FIGURE 1:**
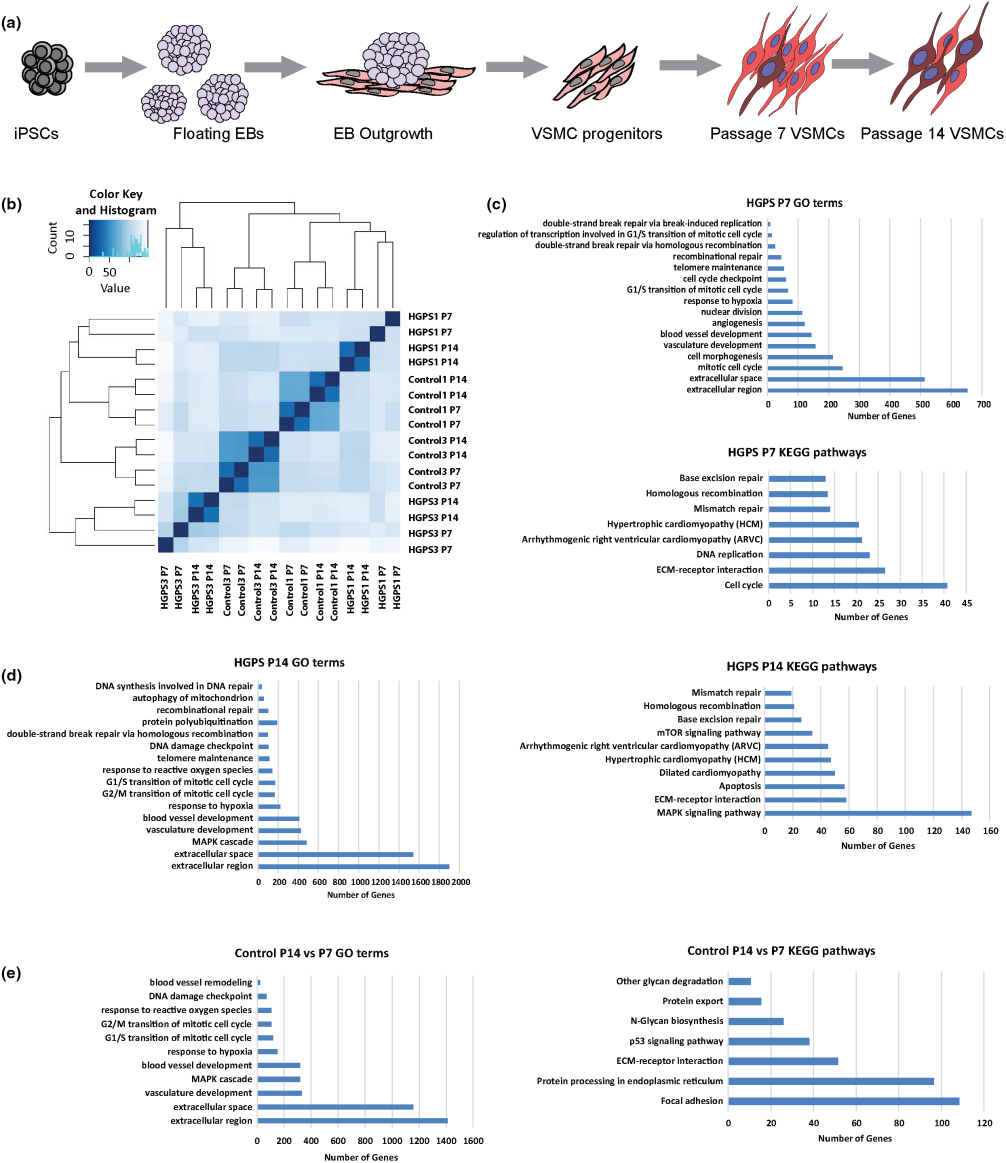
HGPS VSMCs exhibit transcriptomic dysregulation consistent with vascular aging. (a) A schematic of the directed differentiation approach adopted to generate VSMCs from iPSCs through embryoid body (EB) formation. (b) Hierarchical clustering of control [early passage Control1 P7, Control3 P7, and late passage Control1 P14 and Control3 P14] and HGPS VSMC samples [early passage HGPS1 P7, HGPS3 P7, and late passage HGPS1 P14 and HGPS3 P14]. Samples were hierarchically clustered based on their normalized gene expression values. (c) Top panel, gene ontology (GO) term enrichment analysis of HGPS VSMCs vs control at passage 7 (P7) using GOseq. GO terms with *p*‐values <0.001 were considered significantly enriched for differentially expressed genes. The x‐axis represents the number of genes that are differentially expressed, and the y‐axis represents the enriched GO terms. The bottom panel shows KEGG pathway enrichment analysis of HGPS VSMCs vs control at passage 7. KEGG pathways are significantly enriched with *p*‐values <0.05. (d) Left panel shows enriched GO terms of HGPS VSMCs vs control at passage 14 (P14). The right panel shows their enriched KEGG pathways. (e) Left panel shows enriched GO terms of the control VSMCs at passage 14 vs passage 7. The right panel shows the enriched KEGG pathways. All experiments represent two technical replicates of at least 2 cell lines (biological replicates) for each genotype.

To better understand the molecular determinants of vascular aging, we generated VSMCs from HGPS patient‐derived iPSCs via an established directed differentiation strategy (Chen, Chang, et al., 2017) (Figure 1a) and modeled vascular aging in vitro via serial passaging. To assess the efficiency of VSMC differentiation, we quantified the expression of VSMC‐specific markers Calponin (CNN1), Smooth Muscle Actin α2 (ACTA2), Transgelin (TAGLN, SM22α), Smoothelin (SMTN), and Myosin Heavy Chain 11 (MYH11) at both early (passage 7, P7) and late (P14) passages of HGPS and control VSMCs using automated high‐content imaging analysis. The percentages of cells positive for VSMC markers were comparable across genotype and passage with human coronary artery VSMCs (CAVSMCs) (Figure S1a) and confirmed by analysis of mRNA transcript abundance (Figure S1b). Furthermore, physiological contraction in response to the vascular constrictor, angiotensin II was observed across both control and HGPS VSMCs, and CAVSMCs (Figure S1c–e). Thus, the generation of functional VSMCs was unaffected in HGPS iPSCs.

Undifferentiated control and HGPS iPSCs do not express Lamin A or Progerin. Upon the initiation of cellular differentiation, Lamin A is produced, thereby also activating progerin transcription in HGPS cells (Chen, Chang, et al., 2017). By passage 7, close to a third of HGPS VSMCs demonstrated detectable nuclear progerin accumulation, which increased to more than 50% in late (P14) passage cultures, which represent the late stages of the replicative lifespan of HGPS VSMCs (Figure S2a). Similarly, abnormal nuclear shape was detected by automated high‐content imaging analysis in approximately 30% of HGPS VSMCs by passage 7, increasing further in late passage cultures, consistent with the association of structural defects of the nuclear lamina with progerin accumulation (Figure S2b). Rare progerin^+^ cells were detected in aged (P14) wild‐type control VSMC cultures, while nuclear deformation was identified in a passage‐dependent manner in control VSMCs, albeit at a much lower frequency compared to HGPS VSMCs (Figure S2b).

To assess senescence in HGPS and control VSMCs, we first quantified senescence‐activated β‐galactosidase (SA‐βgal) in early and late passage VSMC cultures. The percentage of SA‐βgal^+^ HGPS VSMCs increased from 20% to 40% between passage 7 and 14 (Figure S2c). We next measured the abundance of trimethylated histone H4 lysine 20 (H4K20me3), a histone posttranslational modification increased in senescent cells (Nelson et al., 2016). The percentage of H4K20me3^+^ nuclei increased in both progerin^+^ and progerin^−^ cells within control and HGPS VSMC cultures with passaging (Figure S2d). Thus, serial passaging elicited a progression of progerin accumulation, nuclear defects, and cell senescence in iPSC‐derived HGPS VSMCs, while passaging induced nuclear defects and cell senescence at a reduced frequency in control iPSC‐derived VSMCs with accumulation of progerin in a rare population of cells.

### 3.2 Transcriptional activation of cardiovascular remodeling and oxidative stress response in HGPS VSMCs

To test whether HGPS VSMCs exhibit transcriptional hallmarks of vascular aging, we performed RNA‐sequencing (RNA‐seq) of control and HGPS VSMCs at passage 7 and 14. Hierarchical clustering analysis of the normalized gene expression of the samples showed similar groups corresponding to genotypes (HGPS and control) and cell lines, indicative of high reproducibility (Figure 1b). To identify differentially regulated pathways in HGPS VSMCs, we implemented GO enrichment analysis. Comparing HGPS and control VSMCs at passage 7, we detected a substantial number of pathways associated with aging (Johnson et al., 2015), including stress response (DSB repair and response to hypoxia) and cell fate and proliferation (Figure 1c, Top). We also noted increased extracellular matrix production pathways (extracellular space and extracellular region) that are associated with atherosclerosis (Fisk et al., 2014; Hopkins, 2013). KEGG pathway enrichment analysis revealed dysregulation of DNA damage response and replication pathways as well as cardiovascular disease and atherosclerosis pathways (including cardiomyopathy and ECM‐receptor signaling).

We next examined the transcriptome signatures of HGPS compared to control VSMCs at passage 14. In addition to stress responses, cell fate, and extracellular matrix GO terms, the MAPK signaling cascade is enriched in HGPS late passage VSMCs (Figure 1d, Left), consistent with downstream signaling of ROS and other cellular aging signals (Zhu et al., 2020). Similar to early passage VSMCs, a variety of cardiovascular disease/atherosclerosis KEGG pathways were activated in HGPS compared to control VSMCs at passage 14 (Figure 1d, Right), coherent with the fatal atherosclerosis and vascular smooth muscle deterioration in HGPS (Olive et al., 2010; Varga et al., 2006). Moreover, the number of dysregulated genes within these pathways increased between early and late passage HGPS cultures, analogous to progression of vascular aging and disease.

To determine whether serial passaging increased vascular aging and disease signatures within non‐HGPS VSMCs, we compared passage 14 to passage 7 control VSMCs pathway signatures. Indeed, GO analysis demonstrated that aged control VSMCs are enriched in many of the same known aging pathways as HGPS VSMCs, including stress responses, cell fate, and extracellular matrix GO terms (Figure 1e, Left). In addition to enrichment of ECM‐receptor signaling and related focal adhesion, KEGG pathway analysis identified p53 signaling and pathways related to protein biosynthesis and export (Figure 1e, Right), congruous with age‐induced vascular remodeling. In summary, transcriptional profiling revealed that serial passaging of control and HGPS‐derived VSMCs elicits aging and atherosclerotic transcriptional signatures.

To validate the upregulation of disease‐associated transcripts with protein abundance, we performed immunofluorescent staining for FGF2 and COL4A1. Animal models have shown that FGF2 is important for the manifestation of cardiac hypertrophy (Kardami et al., 2004), whereas COL4A1 is involved in extracellular matrix organization, which has been linked with atherogenesis (Fisk et al., 2014; Hopkins, 2013). Moreover, genome‐wide association studies have correlated COL4A1 expression to coronary heart disease (Yang et al., 2016). Notably, HGPS VSMCs show higher level of FGF2 at late passage than control, as well as late passage compared to early passage (Figure S2E). In addition, HGPS VSMCs exhibit elevated levels of COL4A1 at late passage than early passage (Figure S2F), consistent with higher passage VSMCs exhibiting signatures of cardiovascular disease.

### 3.3 HGPS VSMCs exhibit increased replicative stress

**FIGURE 2:**
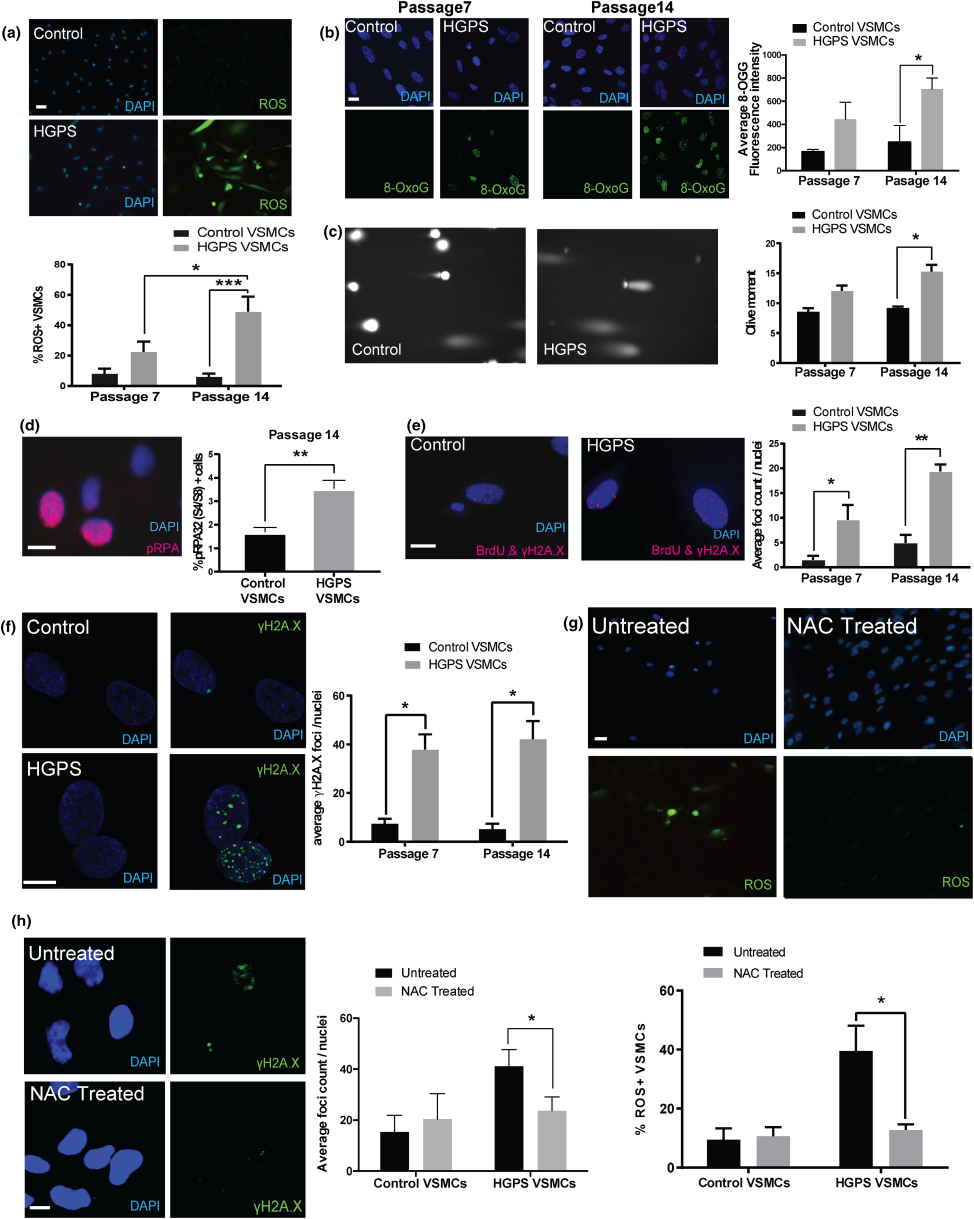
HGPS VSMCs exhibit increased oxidative and DNA replicative stress. (a) HGPS VSMCs exhibit a higher level of ROS. Scale bar = 25 μm. (b) HGPS VSMCs contain significantly higher level of 8‐OxoG compared to control at late passage. Scale bar = 10 μm. (c) DSBs assessed via analysis using alkaline comet assay. (d) HGPS VSMCs exhibit an increased level of replicative stress at late passage as quantified by percentage of pRPA32 (S4/8) positive cells. Scale bar = 25 μm. (e) HGPS VSMCs exhibit increased colocalization between BrdU and γH2A.X in the nucleus measured using the proximity ligation assay and a markedly higher level of DNA damage γH2A.X foci (f). Scale bar = 10 μm. NAC treatment also decreased cellular ROS levels (g) (Scale bar = 25 μm) as well as γH2A.X foci (h) (Scale bar = 10 μm) in HGPS VSMCs. Calculated values represent mean ± SEM (*n* = 6, 2x for 3 cell lines per test group at each passage). All values represent mean ± SEM (*n* = 4, >2x for each cell line per test group).

The transcriptomic data identified an altered response to oxidative stress. Excess production of ROS is common in atherosclerotic plaques and plays an important role in vascular inflammation, irreversible smooth muscle senescence, and arterial remodeling (He & Zuo, 2015; Tavakoli & Asmis, 2012). Consistent with normative vascular aging, HGPS VSMCs demonstrate a significant increase in ROS between early (P7) and late (P14) passages (22% vs 48%), in contrast to control VSMCs that remain less than 10%‐positive cells at these passage numbers (Figure 2a). High levels of ROS can induce DSBs by direct oxidation of DNA nucleotides and the formation of 8‐oxo‐7,8‐dihydro‐2‐deoxyguanine (8‐OxoG) DNA adducts, leading to stalled replication forks (Mahmoudi et al., 2006). Using FITC‐conjugated avidin, which binds to 8‐OxoG with high specificity (Struthers et al., 1998), we detected significantly increased 8‐OxoG signal in HGPS by passage 14 compared to control VSMCs (Figure 2b), demonstrating that ROS‐induced DNA adducts are markedly increased in HGPS VSMCs. The increased oxidized DNA adducts predict increased DNA damage in HGPS VSMCs by passage 14. To confirm DNA damage at the single‐cell level, we performed the alkaline comet assay. Consistent with our findings of increased ROS and 8‐OxoG foci by passage 14, HGPS VSMCs exhibited significantly greater DNA fragmentation compared to control cells by passage 14, measured by the Olive tail moment (Figure 2c).

The increased oxidative damage and altered oxidative stress response suggest that HGPS VSMCs exhibit exacerbated replicative stress. Concordant with this supposition, passage 14 HGPS VSMCs display markedly increased phosphorylated replication protein A (pRPA) (Figure 2d), a critical scaffolding protein for DNA repair and marker of replication stress (Marechal & Zou, 2015; Wootton & Soutoglou, 2021). To quantify active DNA repair at the single‐cell level, the in situ proximity ligation assay (PLA) was used to quantify γH2A.X foci (as a marker of DSBs) associated with synthesized DNA labeled by BrdU. As shown in Figure 2e, HGPS VSMC nuclei contain much higher levels of BrdU‐γH2A.X foci than control VSMCs at both early and late passages. Immunofluorescence of γH2A.X confirmed the increased γH2A.X at early and late passage HGPS VSMCs compared to controls (Figure 2f).

Finally, to assess the impact of ROS on DNA damage and activation of DNA repair, we treated passage 14 control and HGPS VSMCs with the ROS scavenger N‐acetyl Cysteine (NAC). Following NAC treatment, not only did ROS^+^ HGPS VSMCs decrease from 40% to 13% (Figure 2g), γH2A.X foci also dropped from 41 to 24 per nuclei in HGPS VSMCs (Figure 2h). Together, these data show that, like vascular aging and disease (Cervelli et al., 2012; Shah et al., 2018; Wang & Bennett, 2012), iPSC‐derived HGPS VSMCs accumulate ROS‐induced DNA damage.

### 3.4 HGPS VSMCs engage in NHEJ repair on replicating DNA

To tease apart how HGPS VSMCs respond to replication stress, we performed an unbiased mass spectrometry analysis of the proteins recruited to replication forks in control and HGPS VSMCs at early (P7) versus late (P14) stage cultures. We utilized accelerated native isolation of proteins on nascent DNA (aniPOND) (Leung et al., 2013) to immunoprecipitate protein complexes associated with newly synthesized DNA, which were then identified by mass spectrometry. Consistent with the recent finding that Lamin A/C binds nascent DNA and recruits single‐strand DNA‐binding proteins to support replication fork stability (Graziano et al., 2021), lamins were pulled down in both HGPS and control passage 7 VSMCs (Table S1). AniPOND‐mass spectrometry also identified a variety of complexes integral to DNA replication and repair such as DNA topoisomerases and MCM helicases as well as mRNA splicing complexes, consistent with R‐loop formation (Table S1) (Allison & Wang, 2019).

**FIGURE 3:**
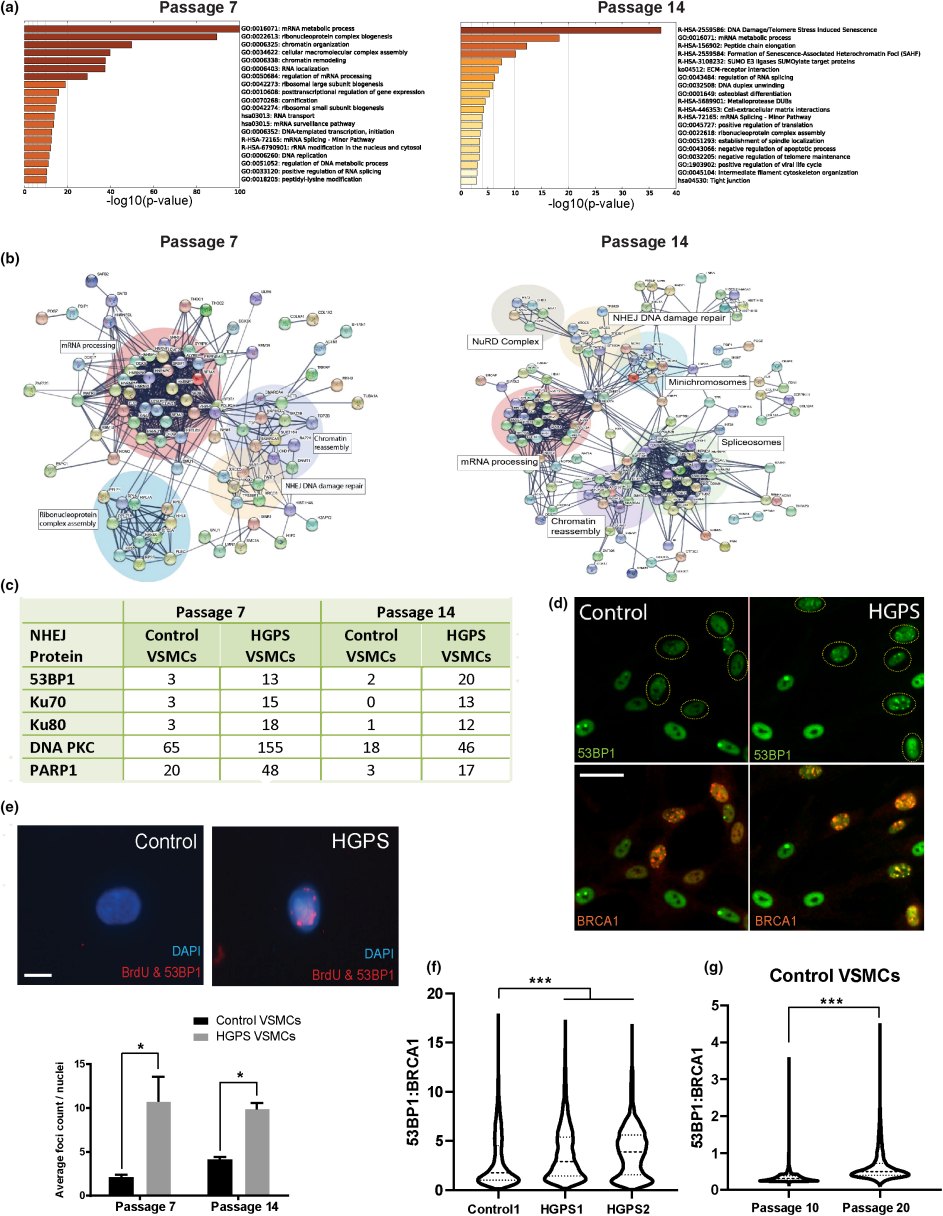
HGPS VMSCs exhibit abnormal engagement with NHEJ repair on replicating DNA. (a) Metascape‐generated heat map of GO enriched terms of differentially enriched proteins from aniPOND‐mass spectrometry analysis from both passage 7 (left) and passage 14 (right). Color indicates *p*‐values of the GO terms. (b) STRING interactome analysis of differentially abundant proteins from aniPOND‐mass spectrometry analysis from passage 7 (left) and passage 14 (right). The confidence level was set to 0.5 (medium). The thickness of the blue lines connecting various proteins represents the level of confidence with which functional partners can be predicted. (c) Quantification of protein abundance (spectra count) of proteins involved in NHEJ repair in control and HGPS VMSCs at both passages from aniPOND‐mass spectrometry analysis. (d) Distribution of 53BP1 foci in control and HGPS VSMCs. The circles are highlighting the G1 cells in the right panel and the presumed S/G2 cells in the left panel. Scale bar = 25 μm. (e) Representative images of proximity ligation assay staining between BrdU and 53BP1 in control and HGPS VSMCs. Red foci represent interactions between BrdU and 53BP1. Bar graphs representing average count of red dots between control and HGPS VSMCs at early and late passages as analyzed by ImageJ. Values represent mean ± SEM (*n* = 2, 2 cell lines per passage per test group). Scale bar = 10 μm. (f) Violin plots represent assembly of 53BP1 and BRCA1 foci that arise at passage 7, and the generation of 53BP1 and BRCA1 foci between passage 10 and 20 control VSMCs (g).

Next, we assessed differential abundance of proteins associated with the replication forks between HGPS and control VSMCs at passage 14. Indicative of increased DNA damage and replication stress in HGPS VSMCs, GO analysis categorized DNA damage and senescence‐associated heterochromatin as prominent enriched pathways in addition to mRNA and peptide processing pathways (Figure 3a, right panel). Differentially abundant proteins were used to generate protein interactome networks, identifying striking differences in the replication fork proteome (Figure 3b, left panel). In particular, the interactome networks identified error‐prone NHEJ DNA damage response pathway as markedly overrepresented in HGPS replication forks compared to control VSMC replication forks.

Strikingly, in addition to mRNA and peptide processing pathways, differentially abundant replication fork‐associated proteins in passage 7 cultures were enriched in chromatin remodeling complex proteins, suggesting altered epigenetic landscapes in aged HGPS VSMCs (Figure 3a, left panel). Further, replication forks in passage 14 HGPS VSMCs are enriched in NHEJ repair networks, minichromosome helicases, spliceosomes and chromatin assembly and remodeling networks including the NuRD complex, compared to control P14 VSMCs (Figure 3b, right panel). At both early and late passages, we observed enrichment for proteins involved in NHEJ repair, such as Ku70, DNA‐PKcs, PARP1, and 53BP1 in HGPS VSMCs compared to control, while the control VSMCs only show repair proteins involved in the processing of replication errors (MSH2/3, FANCI, EXO1) (Figure 3c). This indicates an altered DNA damage response arises soon after the expression of progerin.

To better understand the nature of this alteration, we further probed DNA double‐strand break signaling pathways. Normally, DSBs are repaired by two main mechanisms, NHEJ and homologous recombination (HR). While HR repairs DSBs with great fidelity using the sister chromatid as template for DNA repair, NHEJ is error‐prone, commonly creating small insertions or deletions (Cisneros‐Aguirre et al., 2022). To investigate whether NHEJ is persistently activated in HGPS VSMCs, we stained cells with anti‐53BP1 antibody. Both control and HGPS VSMCs showed cells with high nucleoplasmic signal and typically 1–3 bright foci. These are G1 cells and are thought to reflect DNA damage at under‐replicated regions of the genome such as fragile sites (Spies et al., 2019; Watts et al., 2020), (Harrigan et al., 2011). Strikingly, however, there was an unusual abundance of cells that contained much larger numbers of foci enriched in 53BP1 (Figure 3d). These foci appear in cells that are strongly positive for BRCA1 (Figure 3d, circled). In contrast, the control cells show cells with similar numbers of foci for BRCA1 but the 53BP1 does not accumulate (Figure 3d, circled). The expression and recruitment of BRCA1 is indicative of DNA damage arising during S‐phase, but it is unusual to accumulate 53BP1 at these sites. To directly test whether 53BP1 accumulates in DNA damage sites associated with newly replicated DNA, we performed in situ PLA of BrdU incorporation and 53BP1. This revealed a significant increase in 53BP1 on newly replicating DNA in HGPS VSMCs (Figure 3e), confirming that HGPS cells engage the NHEJ machinery to repair breaks arising during DNA replication.

DNA repair pathway selection involves a competition between 53BP1, which inhibits the DNA end resection necessary for HR, and BRCA1, which promotes DNA end resection and, consequently, stimulates HR‐directed repair (reviewed in (Chen & Sleckman, 2022)). To directly test whether the balance between NHEJ and HR is altered in HGPS VSMCs, we exposed both HGPS and control VSMCs to 3 Gy ionizing radiation and examined the assembly of 53BP1 and BRCA1 foci that arise. By Passage 7, HGPS VSMCs demonstrate higher ratios of 53BP1/BRCA1 foci than control cultures (Figure 3f). Finally, we compared the generation of 53BP1 and BRCA1 foci between passage 10 and 20 control VSMCs. As shown in Figure 3g, control VSMCs demonstrate increased use of NHEJ repair with passaging.

### 3.5 HGPS VSMCs exhibit global histone acetyltransferase MOF and H4K16 acetylation loss

**FIGURE 4:**
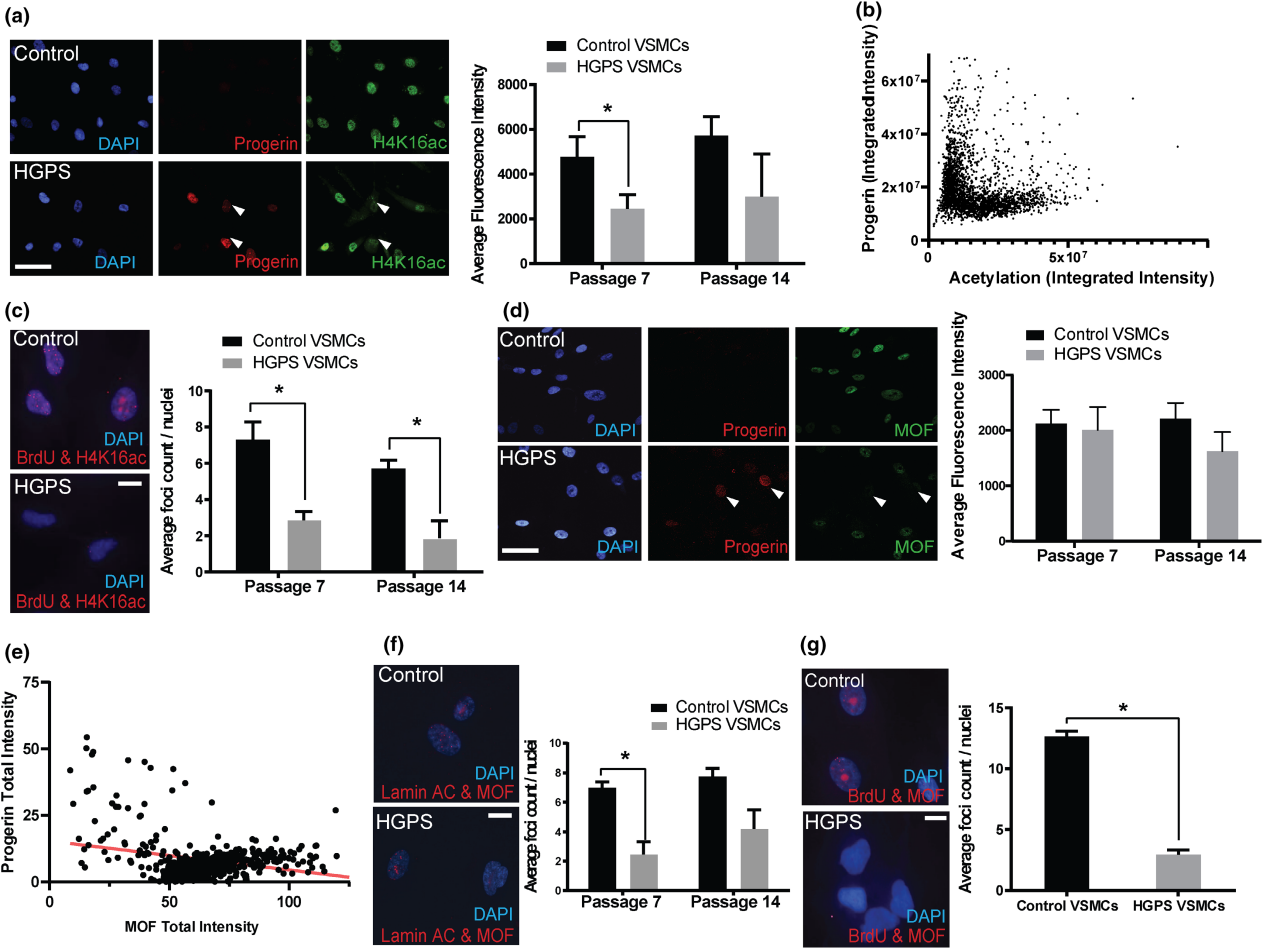
HGPS VSMCs exhibit altered histone acetylation. (a) Representative immunofluorescence images of Progerin and H4K16ac expression in control and HGPS VSMCs. (Right) Bar graphs representing average immunofluorescence intensity of H4K16ac expression between control and HPGS VSMCs at early and late passages. Scale bar = 25 μm. Values represent mean ± SEM (*n* = 4, 2x per cell line, 2 cell lines per passage per test group at each passage). (b) Correlation plot from high‐content immunofluorescence imaging analysis between Progerin and H4K16ac abundance. (c) (Left) Representative images of proximity ligation assay between BrdU and H4K16ac in control and HGPS VSMCs. Red dots represent positive ligation between BrdU and H4K16ac. (Right) Bar graphs representing average count of red dots by ImageJ showing colocalization of H4K16ac and BrdU between control and HPGS VSMCs at early and late passages. Scale bar = 10 μm. Values represent mean ± SEM (*n* = 2, 2 cell lines per passage per test group). (d) (Left) Representative confocal images of immunofluorescence staining for Progerin (red) and MOF (green) proteins in control and HGPS VSMCs. (Right) Bar graphs representing average fluorescence intensity of MOF in both control and HGPS VSMCs at early and late passages. Scale bar = 25 μm. Values represent mean fluorescence intensity ± SEM (*n* = 2, 2 iPSC cell lines per passage per test group). (e) Correlation scatter plot showing immunofluorescence imaging analysis between Progerin and MOF expression in G2/M phase. Red trend line shows inverse correlation. (f) (Left) Representative images of proximity ligation assay between Lamin A/C and MOF in control and HGPS VSMCs. Red dot represents positive ligation between Lamin A/C and MOF. (Right) Bar graphs representing average count of red dots counted by ImageJ analysis showing colocalization of Lamin A/C and MOF in both control and HGPS VSMCs at early and late passages. Scale bar = 10 μm. Values represent mean ± SEM (*n*= > 2, >2 cell lines per passage per test group). (g) (Left) Representative images of proximity ligation assay between BrdU and MOF in control and HGPS VSMCs. Red dot represents positive ligation between BrdU and MOF. (Right) Bar graphs representing average count of red dots by ImageJ showing colocalization of MOF and BrdU between control and HPGS VSMCs at early and late passages. Scale bar = 10 μm. Values represent mean ± SEM (*n*= > 2, >2 cell lines per passage per test group).

53BP1 binding is negatively regulated in S and G2 phase cells through cell cycle regulation of histone acetylation and methylation (Becker et al., 2021; Chapman et al., 2013; Escribano‐Diaz et al., 2013; Hsiao & Mizzen, 2013; Hu et al., 2021; Michelena et al., 2021; Nakamura et al., 2019; Pellegrino et al., 2017). The best‐characterized inhibitory acetylation is H4K16, which interferes with the binding of the 53BP1 Tudor domain to H4K20 methylation (Tang et al., 2013). Histone H4K16 acetylation has been reported to be upregulated in HGPS fibroblasts (Mattioli et al., 2018; Maynard et al., 2019), but downregulated in Zmpste24 mice (Krishnan et al., 2011) and in vitro aged normal human dermal fibroblasts (Gonzalez‐Bermudez et al., 2022). We analyzed H4K16 acetylation in HGPS VSMCs relative to control cell lines and found that global H4K16 acetylation is significantly reduced in HGPS VSMCs (Figure 4a). Plotting progerin expression versus H4K16 acetylation in HGPS VSMCs revealed that high acetylation is almost exclusively observed in low progerin‐expressing cells whereas elevated progerin expression correlates with decreased acetylation (Figure 4b). This demonstrates that the loss of H4K16 acetylation in HGPS VSMCs positively correlates with progerin expression. Using in situ PLA of H4K16ac and BrdU, we further found that H4K16ac is markedly reduced at replicating DNA (Figure 4c).

Histone H4K16 acetylation is deposited by the histone acetyltransferase MOF. MOF and the corresponding H4K16 acetylation have previously been reported to be reduced in the Zmpste24 null mouse fibroblasts (Krishnan et al., 2011). To examine whether global loss of H4K16ac in HGPS VSMCs is due to loss of MOF, we analyzed the expression of MOF via high‐content imaging and found a trend towards reduction of MOF abundance in HGPS VSMCs compared to control at late passage (Figure 4d). MOF was shown to be important in G2/M cell cycle progression (Li et al., 2010; Taipale et al., 2005). Among G2/M cycling cells, high MOF expression is almost exclusively observed in low progerin‐expressing cells, whereas elevated progerin expression correlates with decreased MOF expression (Figure 4e and Figure S2g). In situ PLA analysis of MOF and Lamin A/C showed reduced interaction of MOF with the nuclear lamina (Figure 4f), while in situ PLA analysis of MOF and BrdU demonstrated markedly reduced MOF interaction with replicating DNA (Figure 4g).

### 3.6 Widespread disruption of acetylated H4K16 alters the balance between NHEJ and HR repair in HGPS VSMCs

**FIGURE 5:**
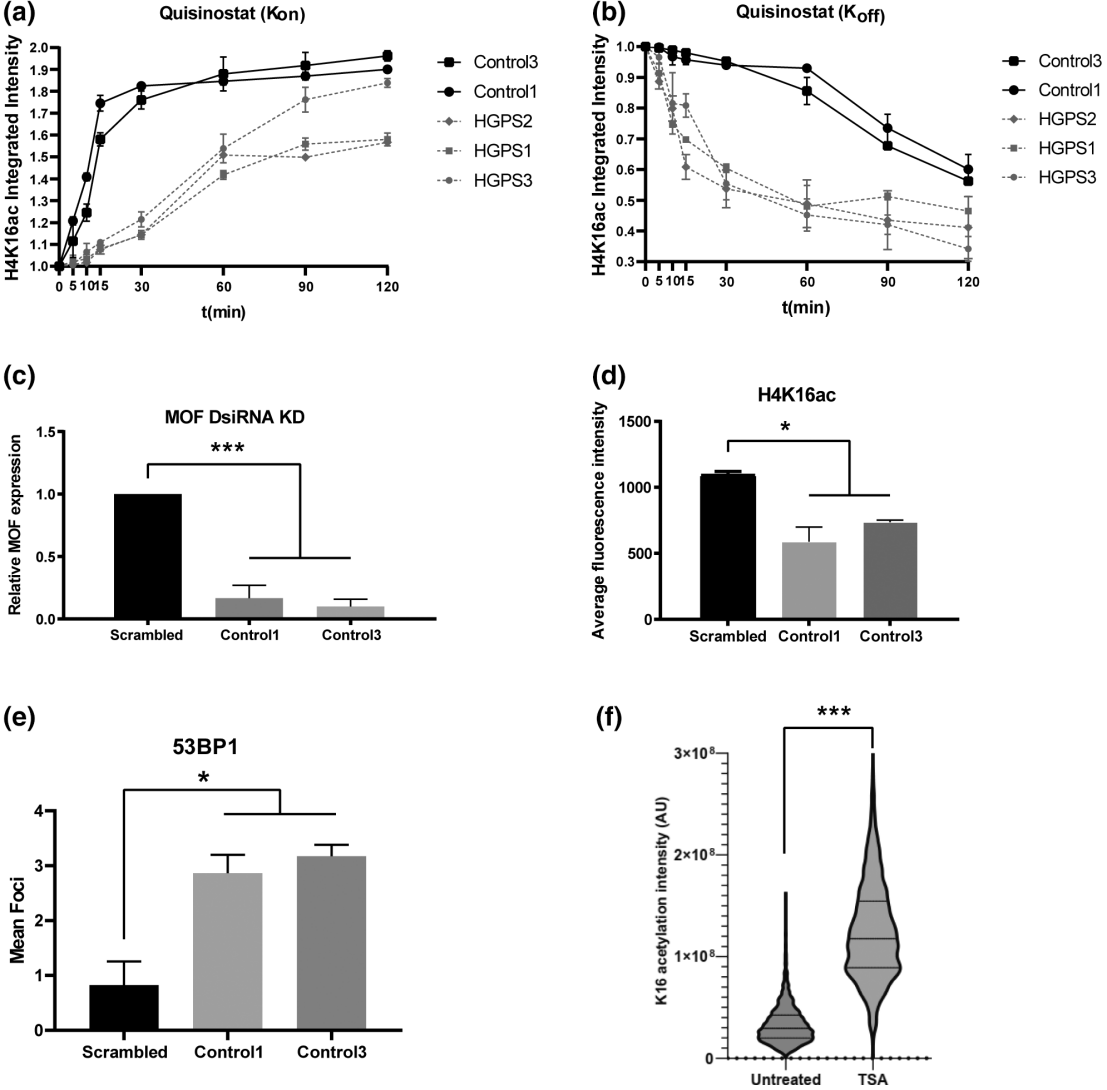
Decreased MOF causes decreased H4K16ac and increased NHEJ events in HGPS VSMCs. (a) Time‐dependent changes in H4K16ac were measured by treating cells with an HDAC inhibitor for 4 h to acetylate H4K16 and then changed the media to study the rate of deacetylation (K‐Off) over different time intervals (0, 5, 10, 15, 30, 60, 90, 120 min). (b) The rate of acetylation (K‐On) was measured by treating cells with the HDAC inhibitor at different intervals (0, 5, 10, 15, 30, 60, 90, 120 min) and observed H4K16 acetylation. Values represent integrated intensity ± SD (n > 2, 3 times per cell line). (c) Relative MOF mRNA expression compared in two control VSMCs to scrambled control following MOF partial knockdown using DsiRNA knockdown strategy. Values represent average expression ± SD (*n* = 2, 2 times per cell line). (d) High‐content imaging fluorescence quantification demonstrating a decrease in H4K16ac expression following MOF knockdown. Values represent average fluorescence intensity ± SD (*n* = 2, 2 times per cell line). (e) High‐content imaging fluorescence quantification demonstrating an increase in 53BP1 expression following MOF knockdown. Values represent average fluorescence intensity ± SD (*n* = 2, 2 times per cell line). (f) Treatment of HGPS VSMCs with HDAC inhibitor TSA leads to increased levels of H4K16ac compared to untreated.

To further determine the underlying factors contributing to the global reduction in H4K16ac levels in HGPS VSMCs, we analyzed the acetylation turnover kinetics at histone H4 lysine 16. The rate of acetylation was determined by a time course in the presence of histone deacetylase inhibitors. For characterizing the kinetics of deacetylation, cells were pretreated with HDAC inhibitor and then time points were assayed following its removal. All three HGPS cell lines showed markedly faster deacetylation of H4K16 than the control lines (Figure 5a). Consistent with a reduction in MOF expression, the three HGPS cell lines all showed a loss of the fast kinetic component of H4K16 acetylation (Figure 5b). Thus, the decreased levels of histone H4K16 acetylation are a result of both decreased acetylation and increased deacetylation. This indicates that these cells retain functional MOF protein but that the steady‐state is biased towards deacetylation in the HGPS VSMCs.

Finally, to test whether decreased H4K16 acetylation mediated the increase in NHEJ activity in HGPS VSMCs, we transiently knocked down MOF in control VSMCs using dicer‐substrate siRNA. Inhibition of MOF expression (Figure 5c) by about 80% decreased global H4K16 acetylation by 2‐fold (Figure 5d). This resulted in a significant increase in 53BP1 foci in control VSMCs (Figure 5e), suggesting that reduced MOF expression and H4K16 acetylation in HGPS VSMCs leads to loss of HR‐directed DNA repair. Treatment of HGPS VSMCs with the HDAC inhibitor TSA for 24 h led to increased H4K16ac levels across the cell population (Figure 5f and Figure S3a). Inhibition of HDACs has previously been reported to inhibit proliferation of VSMCs (Findeisen et al., 2011). We also found that this treatment inhibited proliferation and hence was unsuitable for rescue experiments.

### 3.7 Patient‐derived coronary artery vascular smooth muscle cells exhibit features of HGPS VSMC defects

**FIGURE 6:**
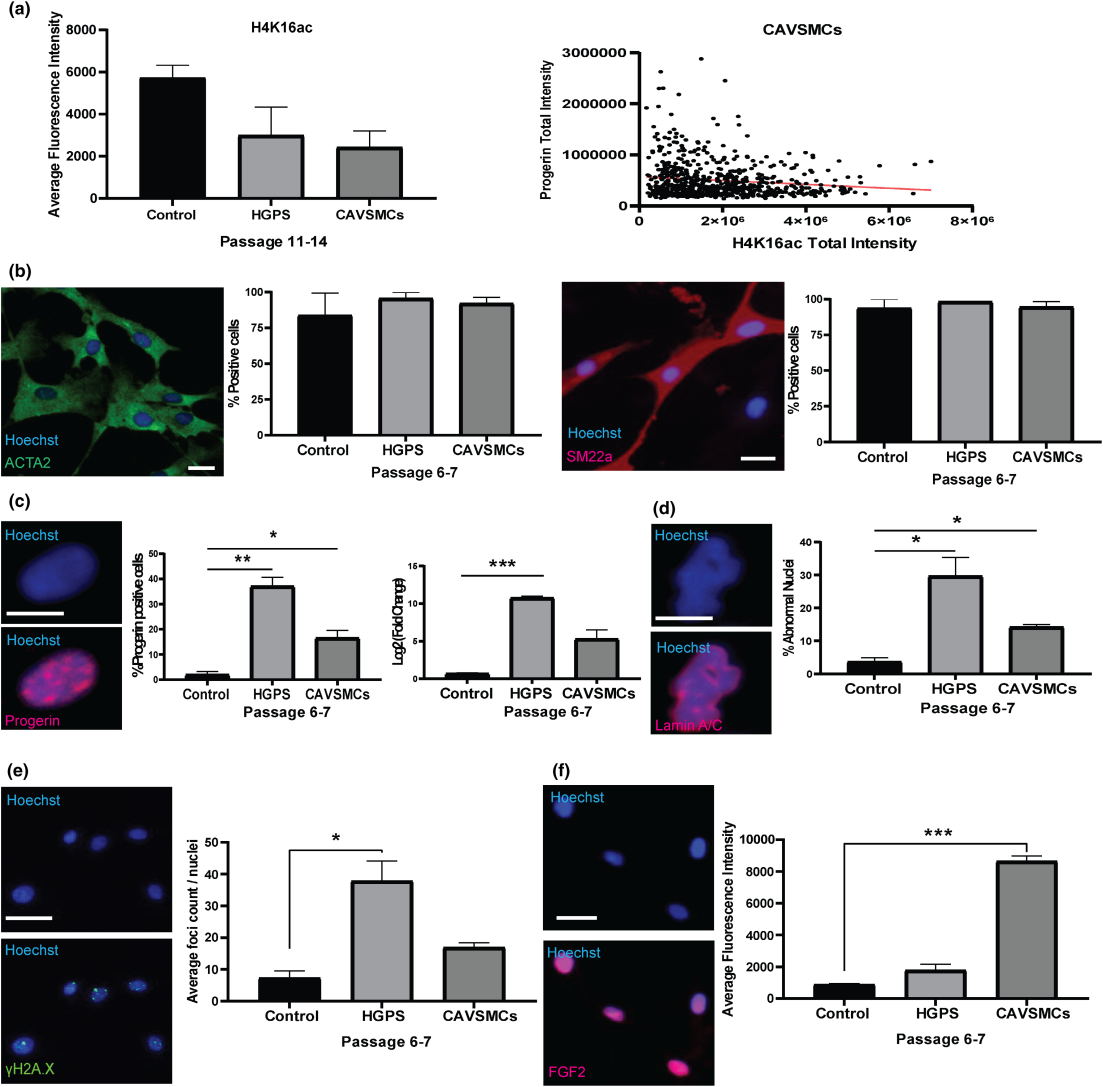
Patient‐derived CAVSMCs exhibit features of HGPS VSMC defects. (a) Quantification of histone mark, H4K16ac in control VSMCs, HGPS VSMCs and CAVSMCs (left). The experiments represent at least two technical replicates of at least 2 cell lines (biological replicates) for each genotype, while values represent mean ± SEM. (Right) Correlation plot from high‐content immunofluorescence imaging analysis between Progerin and H4K16ac expression in patient CAVSMCs. Red trend line shows inverse correlation. (b) Primary CAVSMCs from aged donors express typical VSMC markers such as ACTA2, and SM22alpha. Scale bar = 25 μm. (c) Representative images (left), quantification (middle) of primary aged CAVSMCs expressing Progerin compared to iPSC‐derived control and HGPS VSMCs. (Right) Progerin transcript levels assessed by qPCR. Scale bar = 10 μm. (d) High‐content scanning images of Lamin A/C staining demonstrate that both HGPS VSMCs and primary aged CAVSMCs display increased incidence of abnormal nuclear morphology compared to control VSMCs. Scale bar = 10 μm. (e) Primary aged CAVSMCs show increased level of DNA damage γH2A.X foci. Scale bar = 25 μm. (f) Primary aged CAVSMCs exhibit elevated levels of FGF2. Scale bar = 25 μm. The experiments represent at least two technical replicates of at least 2 cells lines (biological replicates) from organ donors (>50 years old), while values represent mean ± SEM.

The role of H4K16 acetylation in vascular aging and disease has not been reported. The cellular and molecular pathologies in HGPS are notably similar to vascular disease (Miyamoto et al., 2014; Olive et al., 2010; Silvera et al., 2013). Hence, we sought to compare HGPS‐derived VSMCs to primary coronary artery VSMCs (CAVSMCs). Organ‐donated hearts that were unsuitable for transplantation were obtained under a research ethics board‐approved protocol. Histological examination of the coronary arteries using Hematoxylin and Eosin and Verhoeff‐van Gieson stains showed noted thickening of intima‐media of donors 1 and 2 compared to donor 3, suggestive of atherosclerosis (Figure S3b). We next sought to compare H4K16 acetylation levels between iPSC‐derived VSMCs and CAVSMCs. HGPS VSMCs and CAVSMCs showed reduced H4K16 acetylation compared to control VSMCs. Moreover, H4K16 acetylation levels in CAVSMCs is correlated with progerin expression (Figure 6a). CAVSMC cultures contained comparable percentages of cells expressing VSMC markers as iPSC‐derived VSMCs (Figure 6b). We detected levels of progerin in the CAVSMCs that are higher than iPSC‐derived control VSMCs but lower than HGPS VSMCs, consistent with the progerin expression in coronary artery tissues of non‐HGPS individuals (Olive et al., 2010), the mRNA levels were validated by qPCR (Figure 6c). Similarly, nuclear deformation was detected in approximately 15% of CAVSMCs (Figure 6d), and γH2A.X immunofluorescence staining showed lower DNA damage levels in CAVSMCs than HGPS VSMCs but higher than control VSMCs (Figure 6e). The primary aged CAVSMCs and HGPS VSMCs exhibit elevated levels of FGF2 (Figure 6f), which is associated with atherosclerosis (Schulz et al., 2005). Taken together, our data demonstrate close association between features of HGPS and normative vascular aging and identify a potential role of H4K16 acetylation in vascular aging and disease.

## 4 DISCUSSION

In this study, we employed a tractable model of vascular aging using iPSC‐derived VSMCs from HGPS patients to follow the progression of cellular aging phenotypes and begin to tease apart the molecular underpinnings of these phenotypes. We have previously shown that reprogramming HGPS fibroblasts restores a normal epigenome and nuclear structure to HGPS iPSCs, which do not express Lamin A or progerin (Chen, Chang, et al., 2017). Upon differentiation into VSMCs, we observed the progression of progeria and vascular aging cellular phenotypes in a passage‐dependent manner. Furthermore, by passage 14, control iPSC‐derived VSMCs also demonstrated overlapping phenotypes including rare progerin^+^ cells, evidence of replication stress, DNA damage and nuclear deformation, albeit at a reduced frequency. At the transcriptome level, the aging HGPS VSMCs demonstrated marked upregulation of pathways associated with cardiovascular disease including extracellular matrix remodeling, MAPK and mTOR signaling, as did control iPSC‐derived VSMCs, although at a diminished level.

One of the hallmarks of atherosclerotic progression in the aging vasculature is increased oxidative stress in tandem with vascular inflammation and remodeling (Batty et al., 2022). HGPS cells have been reported to display altered redox homeostasis (Kubben et al., 2016; Richards et al., 2011). We demonstrated not only that HGPS VSMCs exhibit consistently elevated cellular ROS once progerin is expressed, but also that high levels of ROS induce direct DNA damage by oxidizing nucleotides to create DNA lesions. Consistent with this, RNA‐sequencing revealed dysregulation of oxidative stress response programs at both early and late passage, corroborating our findings that HGPS VSMCs exhibit perturbed redox homeostasis in vitro. Treating HGPS VSMCs via NAC treatment resulted in a profound decrease in ROS‐induced DNA damage. Thus, elevated ROS contributes significantly to DNA damage, and the inclusion of restoring redox balance would be beneficial to HGPS patients.

Increased ROS and the activation of premature senescence in VSMCs are major underlying factors for atherosclerotic plaque formation (Nakano‐Kurimoto et al., 2009; Wang et al., 2015). The elevated oxidative stress is expected to lead to increased replicative stress and DNA damage during DNA replication. To identify complexes differentially recruited to HGPS and control replication forks, we coupled aniPOND purification to mass spectrometry. We report an overall increase in NHEJ proteins, including DNA‐PKcs, Ku70, Ku80 and 53BP1 on replicating DNA in HGPS VSMCs. Upon formation of a DSB, Ku70/80 heterodimer and MRE11 compete to process the DSB (Ismail et al., 2015). 53BP1 and BRCA1 are competitive regulators of pathway choice (Callen et al., 2020). During S‐phase, HR repair is favored by epigenetic changes that inhibit 53BP1 assembly and promote BRCA1 assembly. When single‐ended DSBs arise at sites of replication stress, failure to displace the Ku70/80 heterodimer results in abortive HR repair and toxic end joining (Britton et al., 2020; Sharma et al., 2021). Thus, the increased recruitment of NHEJ proteins to replication forks may significantly contribute to increased levels of unrepaired DNA damage in HGPS VSMCs, genomic instability, and senescence.

Broad changes in the epigenetic landscape have been widely described in HGPS (Ashapkin et al., 2019). Histone posttranslational modifications are markedly dissimilar between HGPS and control cells, with the tendency to lose the binary partition of the genome into euchromatin and heterochromatin (Chojnowski et al., 2020; Goldman et al., 2004). However, the role of histone acetylation, including H4K16 acetylation, in HGPS and normative aging is unclear. For example, it has been reported that HGPS fibroblasts have increased levels of histone H4K16 acetylation (Mattioli et al., 2018). Consistent with this, Lamin A knockout mouse embryonic fibroblasts (MEFs) and HGPS human fibroblasts were reported to downregulate SIRT1 (Maynard et al., 2022), a reported histone H4K16 deacetylase (Vaquero et al., 2004). In contrast, the Zmpste24 mouse model of HGPS demonstrated an association of histone H4K16 hypoacetylation with premature senescence that could be reversed by MOF overexpression or by histone deacetylase inhibition (Krishnan et al., 2011). Additionally, decreased H4K16 acetylation and changes in histone acetylation have been linked to normative aging (Gonzalez‐Bermudez et al., 2022; Krishnan et al., 2011; Peleg et al., 2016). Here, we observed a loss of H4K16 acetylation in HGPS compared to control VSMCs, while the association of the histone acetyltransferase MOF with nascent DNA was reduced in HGPS VSMCs. Additionally, we found that the subunits of the NuRD complex, including MTA2, HDAC1 and HDAC2 are enriched at the HGPS replication forks. Consistent with reduced HAT recruitment and increased HDAC recruitment, kinetic analysis revealed decreased MOF HAT activity and increased H4K16 HDAC activity both contribute to the reduced levels of H4K16 acetylation. In HGPS VSMCs, the reduced levels of H4K16 acetylation were partially reversed by treatment with the HDAC inhibitor TSA. This contrasts with the reported loss of H4K16 acetylation (Maynard et al., 2022) and NuRD complex components reported in HGPS patient dermal fibroblasts (Pegoraro et al., 2009). Overall, these findings highlight the potential for tissue‐specific regulation of H4K16 acetylation levels and its impact on HGPS and normative aging.

Finally, our aniPOND‐MS results identified the RNA acetyltransferase NAT10 was increased in replicating DNA in HGPS VSMCs. This is consistent with several studies showing overexpression of NAT10 drives HGPS phenotypes including aberrant nuclear morphology (Balmus et al., 2018; Larrieu et al., 2014). In concert with the robustly altered histone methylation, taken together these data support abnormal histone acetylation dynamics in HGPS.

It is well established that progerin expression is associated with increased steady‐state DNA double‐strand breaks in HGPS patient cells (Constantinescu et al., 2010; Liu et al., 2006; Liu et al., 2008), in primary cells of normal human donors of advanced age (Ragnauth et al., 2010), and in cells that accumulate farnesylated prelamin A during normative aging or through related premature aging syndromes (di Masi et al., 2008; Krishnan et al., 2011; Liu et al., 2005; Liu et al., 2006; Musich & Zou, 2009). These breaks arise primarily during S‐phase (Chojnowski et al., 2020; Wheaton et al., 2017). After DNA replication, changes in epigenetic marks contribute to inhibiting the assembly of 53BP1 and NHEJ proteins while promoting the assembly of BRCA1 and HR proteins (Hsiao & Mizzen, 2013; Jacquet et al., 2016; Nakamura et al., 2019; Tang et al., 2013). Synthetically targeted DSBs also show a dependence on MOF and the acetylation of H4K16 to switch to the HR pathway (Horikoshi et al., 2019). Consistent with this, HDACs 1 and 2 have been implicated in deacetylating histone H4K16 and are required for NHEJ repair (Miller et al., 2010; Wu et al., 2017). As an additional layer of control, they are also involved in removing an inhibitory acetylation of 53BP1 itself, which requires HDAC2 (Guo et al., 2018). Using DNA DSB reporter constructs for DNA repair pathway choice integrated into fibroblast cell lines engineered to express progerin, progerin expression results in increased error‐prone end‐joining repair (Joudeh et al., 2023; Komari et al., 2020). We find that the HGPS VSMCs lose H4K16 acetylation through both the downregulation of MOF and its associated HAT activity and the upregulation of the opposing deacetylase activities. This results in both reduced steady‐state H4K16 acetylation and reduced lifetime of the acetylated state. The H4K16 acetylation‐dependent failure to displace 53BP1 has previously been implicated in the assembly of NHEJ proteins on collapsed replication forks upon disruption of the Fanconi Anemia pathway (Renaud et al., 2016).

Persistent (unrepaired) DNA damage is thought to contribute to senescence. There are other defects in the DNA damage response pathway and DNA repair that may result in the failure to repair DSBs. Reduced PCNA recruitment, inappropriate recruitment of XPA, and inefficient activation of DNA PKcs have all been reported in progerin‐expressing cell lines (Ghosh et al., 2015; Hilton et al., 2017). Since small numbers of large DSB foci, characteristic of breaks arising during the previous interphase (Harrigan et al., 2011; Lukas et al., 2011), are found in HGPS VSMCs, the initiation of the NHEJ pathway on breaks arising in newly replicated chromatin may result in the eventual failure of DSB repair and the persistence of DNA damage.

Our results may seem to be at odds with results in Zmpste24 mouse embryonic fibroblasts (Krishnan et al., 2011) and in vitro aged human dermal endothelial cells (Gonzalez‐Bermudez et al., 2022) where the reduction in histone H4K16 acetylation is reportedly associated with a reduction in 53BP1 binding. More precisely, a reduced rate of assembly of 53BP1 was reported. This is not likely specific to 53BP1, since the upstream factor, MDC1, and the opposing BRCA1 have also been found to have altered assembly in MOF null cells (Li et al., 2010). This highlights another potential feature of the depletion of H4K16 acetylation. The acetylation inhibits the association of the histone H4 N‐terminus with the nucleosome acidic patch. This promotes increased nucleosome‐nucleosome interactions and is expected to reduce DNA accessibility (Chen, Yang, et al., 2017; Zhang et al., 2017). Hence, the loss of this acetylation may also reduce the efficiency of these reactions and contribute to the persistence of unrepaired DNA damage, present as 53BP1 nuclear bodies in G1 (Spies et al., 2019; Watts et al., 2020).

The generation of DSBs in progerin‐expressing cells is reportedly biased towards late S‐phase when heterochromatin replicates. The loss of heterochromatin modifications associated with the promotion of HR such as H3K9me3 (Abu‐Zhayia et al., 2018; Alagoz et al., 2015) and H3K27me3 (Schep et al., 2021) in HGPS VSMCs at both early and late passages may promote the formation of unrepairable lesions and the activation of transposons to contribute to the profound transcriptomic differences observed at corresponding passages in HGPS cells (Della Valle et al., 2022; Soto‐Palma et al., 2022).

Aberrant regulation of histone‐modifying enzymes due to defective nuclear lamina has been correlated with lifespan in several aging models (Cesarini et al., 2015; Krishnan et al., 2011; Liu, Drozdov, et al., 2013). Moreover, Lamin A/C has been shown to be a target of acetylation by MOF and that MOF depletion leads to catastrophic genomic defects, including weakened nuclear lamina stability (Karoutas et al., 2019). We demonstrate here that the accumulation of progerin correlates with the loss of H4K16 acetylation and is associated with decreased association with Lamin A/C. Notably, MOF has been shown to contribute to oxidative stress resistance (Ikeda et al., 2017; Qiao et al., 2014). Therefore, impaired MOF activity could very well contribute to elevated oxidative stress in HGPS VSMCs. Consistent with this supposition, knockdown of MOF in control VSMCs led to increased levels of 53BP1 foci, recapitulating the increased levels of 53BP1 in HGPS VSMCs.

Recent studies demonstrated that histone acetylation plays an important role in regulating the aging of vascular cells (Ghosh, 2021; Lee et al., 2020; Sun et al., 2021), although H4K16 acetylation has not been previously studied. In particular, the acetylation status of histones has been linked to the expression of genes that control cellular senescence. Senescent cells are known to accumulate in aging tissues, and their presence has been linked to various age‐related diseases, including cardiovascular disease. Finally, we provide evidence of the presence of progerin in the patient‐derived CAVSMCs of non‐HGPS individuals, indicating to the infrequent use of the cryptic splice site in exon 11 of LMNA gene. Interestingly, in addition to progerin accumulation, abnormal nuclei, our results show a decrease in H4K16 acetylation in CAVSMCs similar to HGPS VSMCs. The similarity between the defects observed in CAVSMCs and HGPS VSMCs suggests underlying molecular mechanisms that contribute to HGPS pathology and age‐related cardiovascular diseases. The detection of atherosclerosis lesions in the aged organ donors is comparable with the work from Leslie Gordon's lab (Olive et al., 2010) which observed progerin production in the coronary artery vascular cells of non‐HGPS individuals. In patients with dilated cardiomyopathy, progerin transcript levels were elevated in heart vascular cells compared to control tissues (Messner et al., 2018), consistent with the progerin mRNA increase in our aged primary CAVSMCs. Additionally, other studies have observed accumulation of wild‐type prelamin A in human aortic VSMCs (Cao et al., 2011; Liu, Drozdov, et al., 2013; Ragnauth et al., 2010), further strengthening that the dysregulation of Lamin A may be used as a biomarker for VSMC aging and disease in the general population. Understanding the cross‐talk between histone acetylation and the regulation of Lamin A could lead to the development of new treatments for these conditions.

Overall, we elucidated a mechanism for impaired histone acetylation in HGPS iPSC‐derived VSMCs, which contributes to defective recruitment of DNA repair proteins. The close correlation between dysregulated histone acetylation and the onset of cardiovascular diseases has been established previously (Kwon et al., 2020). How defective histone acetylation contributes to the pathophysiology of vascular disease remains elusive. We established a paradigm for the role of nuclear lamina in regulating histone acetylation and genomic stability in the context of vascular aging, providing a platform to uncover potential drugs targeting epigenetic enzymes to treat cardiovascular diseases during normative aging.

## AUTHOR CONTRIBUTIONS

**Methodology:** Mzwanele Ngubo, Zhaoyi Chen, Amit Shrestha, Darin McDonald, Julien Yockell‐Lelièvre, Aurélie Laurent, Rana Karimpour; **Formal analysis:** Mzwanele Ngubo, Zhaoyi Chen, Amit Shrestha, Darin McDonald, Julien Yockell‐Lelièvre, Ojong Tabi Ojong Besong, Rana Karimpour; **Writing original draft:** Mzwanele Ngubo, Zhaoyi Chen, Amit Shrestha, F. Jeffrey Dilworth, Michael J. Hendzel, and William L. Stanford; **Conceptualization:** Eve C. Tsai, F. Jeffrey Dilworth, Michael J. Hendzel, William L. Stanford; **Funding acquisition:** Michael J. Hendzel, William L. Stanford, F. Jeffrey Dilworth.

## CONFLICT OF INTEREST STATEMENT

None declared.

## Supporting information


Figure S1.

Figure S2.

Figure S3.

Table S1.

Data S1.

## Data Availability

RNA‐seq data were deposited in the NCBI GEO with accession number GSE231761. The other data that support the findings of this study are available upon reasonable request.
