# Supplementary figures and images for: Progeria‐based vascular model identifies networks associated with cardiovascular aging and disease
Source: Aging Cell. 2024 Apr 4;23(7):e14150. doi: 10.1111/acel.14150 (PMC11258467; doi:10.1111/acel.14150)

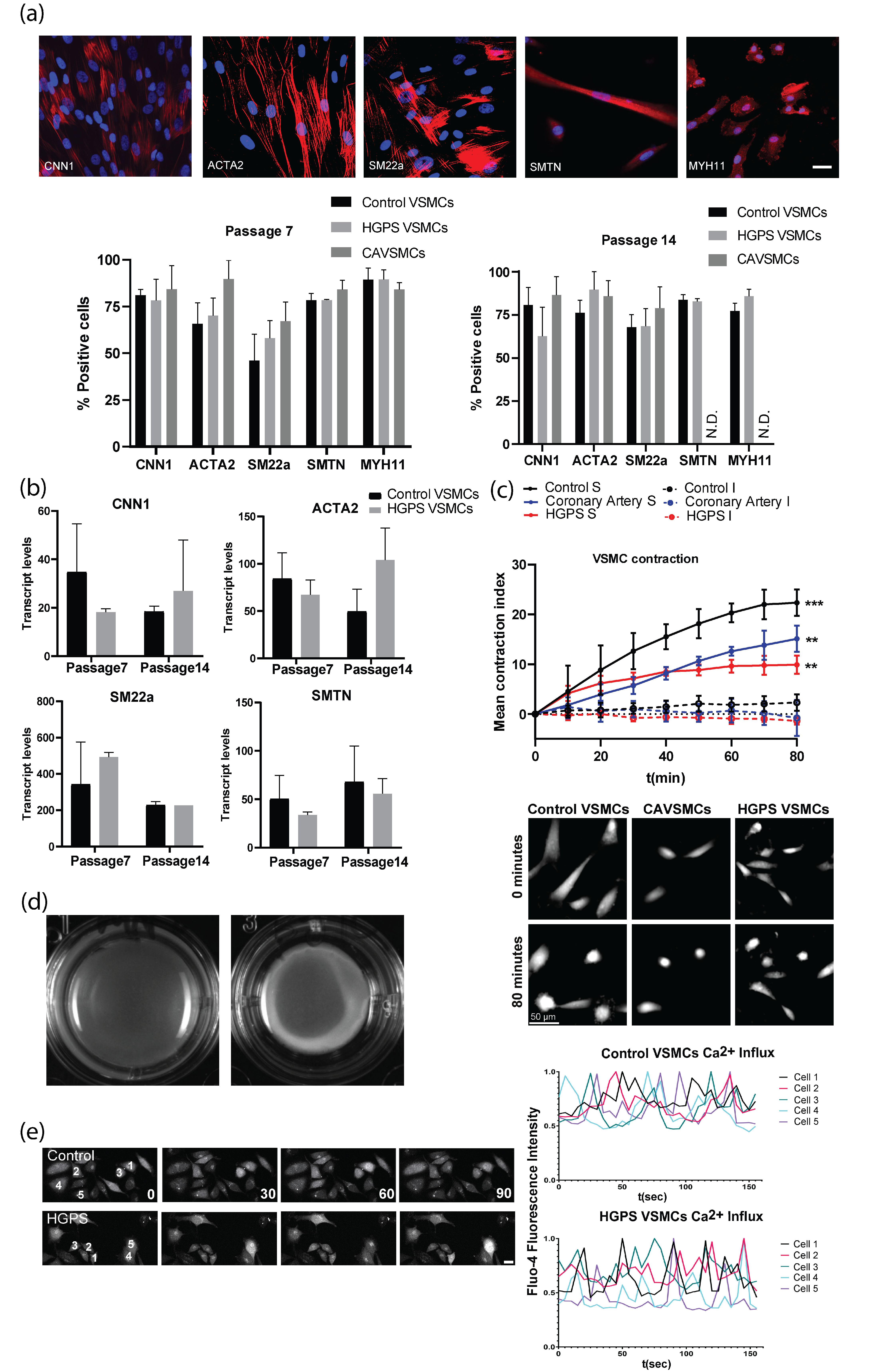

Supplement: Supplementary file 1 — Figure S1. [file ACEL-23-e14150-s001.tif]

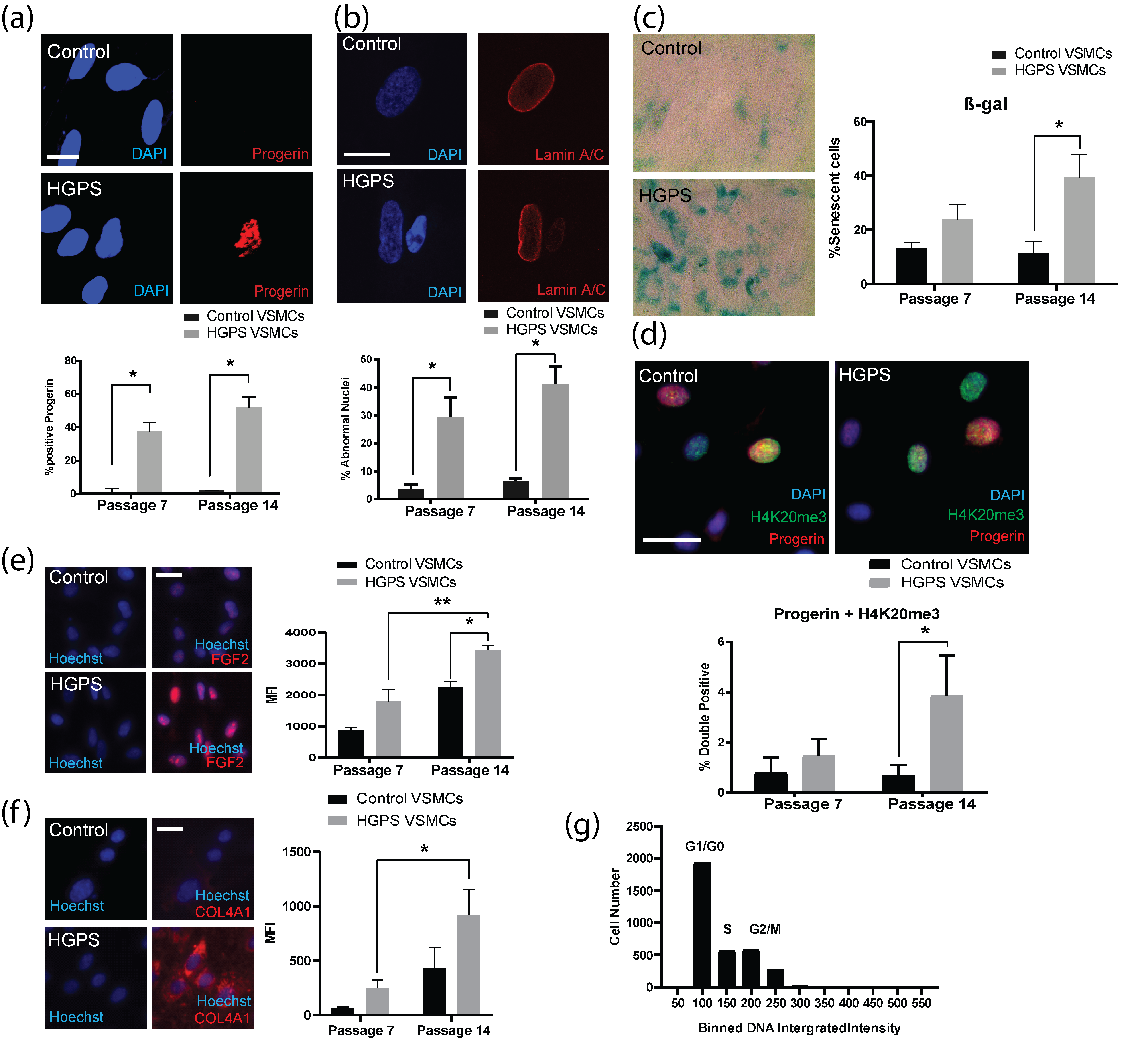

Supplement: Supplementary file 2 — Figure S2. [file ACEL-23-e14150-s004.tif]

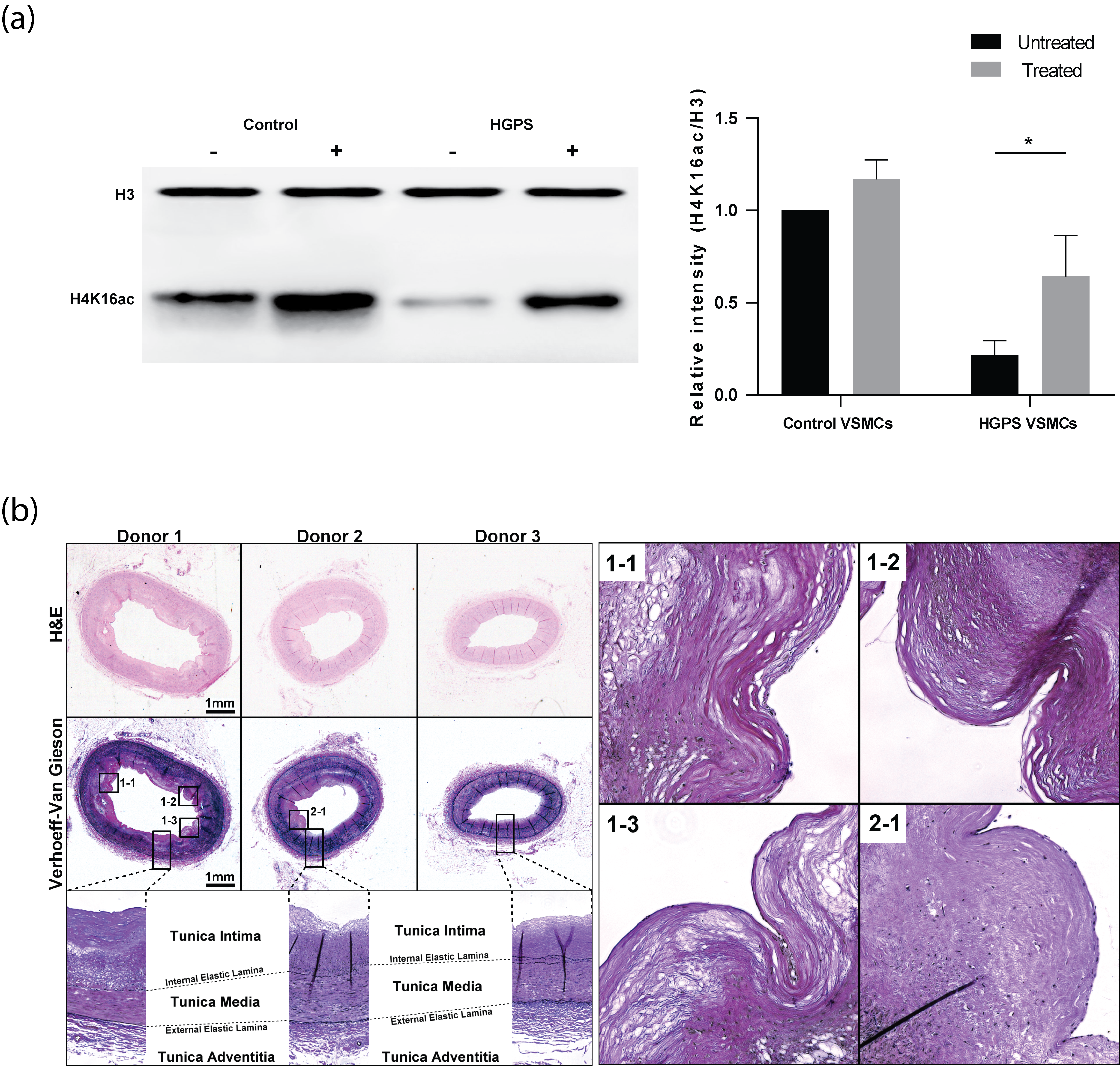

Supplement: Supplementary file 3 — Figure S3. [file ACEL-23-e14150-s002.tif]
