# Supplementary material for: Progeria‐based vascular model identifies networks associated with cardiovascular aging and disease
Source: Aging Cell. 2024 Apr 4;23(7):e14150. doi: 10.1111/acel.14150 (PMC11258467; doi:10.1111/acel.14150)
Supplement: Supplementary file 4 — Table S1. [file ACEL-23-e14150-s005.pdf]

P7 aniPOND DP583-d, Samples report created on 06/03/2021
Experiment: P7 aniPOND DP583-d
Peak List Generator: unknown
Version: unknown
Charge States Calculated: unknown
Deisotoped: unknown
Textual Annotation: unknown
Database Set: 1 Database
Database Name: the contaminants_custom database
Version: unknown
Taxonomy: Homo sapiens
Number of Proteins: 20453
Does database contain common contaminants?: unknown
Search Engine Set: 1 Search Engine
Search Engine: Mascot
Version: 2.5.0
Samples: All Samples
Fragment Tolerance: 0.60 Da (Monoisotopic)
Parent Tolerance: 10.0 PPM (Monoisotopic)
Fixed Modifications: +57 on C (Carbamidomethyl)
Variable Modifications: -17 on Peptide N-Terminal (Gln->pyro-Glu), +16 on M (Oxidation), +40 on Peptide N-Terminal (Pyro-carbamidomethyl), +42 on Peptide N-Terminal (Acetyl)
Database: the contaminants_custom database (selected for Homo sapiens, unknown version, 20453 entries)
Digestion Enzyme: Trypsin
Max Missed Cleavages: 2
Probability Model:

DP583-1 (F	Classifier d	Good (68%)	IonSc	IdentityScore m:56.7/s:2	Ion Score n	Identity Sc	Homology	Bad (32%)	IdentityScc	Ion Score m:25.0/s:0.0359	Identity Sc Homology [all charge states]
DP583-1 (F	Classifier d	Good (68%)	IonSc	IdentityScore m:56.7/s:2	Ion Score n	Identity Sc	Homology	Bad (32%)	IdentityScc	Ion Score m:25.0/s:0.0359	Identity Sc Homology [all charge states]
DP583-1 (F	Classifier d	Good (68%)	IonSc	IdentityScore m:56.7/s:2	Ion Score n	Identity Sc	Homology	Bad (32%)	IdentityScc	Ion Score m:25.0/s:0.0359	Identity Sc Homology [all charge states]
DP583-1 (F	Classifier d	Good (68%)	IonSc	IdentityScore m:56.7/s:2	Ion Score n	Identity Sc	Homology	Bad (32%)	IdentityScc	Ion Score m:25.0/s:0.0359	Identity Sc Homology [all charge states]
DP583-1 (F	Classifier d	Good (68%)	IonSc	IdentityScore m:56.7/s:2	Ion Score n	Identity Sc	Homology	Bad (32%)	IdentityScc	Ion Score m:25.0/s:0.0359	Identity Sc Homology [all charge states]
DP583-1 (F	Classifier d	Good (68%)	IonSc	IdentityScore m:56.7/s:2	Ion Score n	Identity Sc	Homology	Bad (32%)	IdentityScc	Ion Score m:25.0/s:0.0359	Identity Sc Homology [all charge states]
DP583-1 (F	Classifier d	Good (68%)	IonSc	IdentityScore m:56.7/s:2	Ion Score n	Identity Sc	Homology	Bad (32%)	IdentityScc	Ion Score m:25.0/s:0.0359	Identity Sc Homology [all charge states]
DP583-1 (F	Classifier d	Good (68%)	IonSc	IdentityScore m:56.7/s:2	Ion Score n	Identity Sc	Homology	Bad (32%)	IdentityScc	Ion Score m:25.0/s:0.0359	Identity Sc Homology [all charge states]
DP583-1 (F	Classifier d	Good (68%)	IonSc	IdentityScore m:56.7/s:2	Ion Score n	Identity Sc	Homology	Bad (32%)	IdentityScc	Ion Score m:25.0/s:0.0359	Identity Sc Homology [all charge states]
DP583-1 (F	Classifier d	Good (68%)	IonSc	IdentityScore m:56.7/s:2	Ion Score n	Identity Sc	Homology	Bad (32%)	IdentityScc	Ion Score m:25.0/s:0.0359	Identity Sc Homology [all charge states]
DP583-1 (F	Classifier d	Good (68%)	IonSc	IdentityScore m:56.7/s:2	Ion Score n	Identity Sc	Homology	Bad (32%)	IdentityScc	Ion Score m:25.0/s:0.0359	Identity Sc Homology [all charge states]
DP583-1 (F	Classifier d	Good (68%)	IonSc	IdentityScore m:56.7/s:2	Ion Score n	Identity Sc	Homology	Bad (32%)	IdentityScc	Ion Score m:25.0/s:0.0359	Identity Sc Homology [all charge states]
DP583-1 (F	Classifier d	Good (68%)	IonSc	IdentityScore m:56.7/s:2	Ion Score n	Identity Sc	Homology	Bad (32%)	IdentityScc	Ion Score m:25.0/s:0.0359	Identity Sc Homology [all charge states]
DP583-1 (F	Classifier d	Good (68%)	IonSc	IdentityScore m:56.7/s:2	Ion Score n	Identity Sc	Homology	Bad (32%)	IdentityScc	Ion Score m:25.0/s:0.0359	Identity Sc Homology [all charge states]
DP583-1 (F	Classifier d	Good (68%)	IonSc	IdentityScore m:56.7/s:2	Ion Score n	Identity Sc	Homology	Bad (32%)	IdentityScc	Ion Score m:25.0/s:0.0359	Identity Sc Homology [all charge states]
DP583-1 (F	Classifier d	Good (68%)	IonSc	IdentityScore m:56.7/s:2	Ion Score n	Identity Sc	Homology	Bad (32%)	IdentityScc	Ion Score m:25.0/s:0.0359	Identity Sc Homology [all charge states]
DP583-1 (F	Classifier d	Good (68%)	IonSc	IdentityScore m:56.7/s:2	Ion Score n	Identity Sc	Homology	Bad (32%)	IdentityScc	Ion Score m:25.0/s:0.0359	Identity Sc Homology [all charge states]
DP583-1 (F	Classifier d	Good (68%)	IonSc	IdentityScore m:56.7/s:2	Ion Score n	Identity Sc	Homology	Bad (32%)	IdentityScc	Ion Score m:25.0/s:0.0359	Identity Sc Homology [all charge states]
DP583-1 (F	Classifier d	Good (68%)	IonSc	IdentityScore m:56.7/s:2	Ion Score n	Identity Sc	Homology	Bad (32%)	IdentityScc	Ion Score m:25.0/s:0.0359	Identity Sc Homology [all charge states]
DP583-1 (F	Classifier d	Good (68%)	IonSc	IdentityScore m:56.7/s:2	Ion Score n	Identity Sc	Homology	Bad (32%)	IdentityScc	Ion Score m:25.0/s:0.0359	Identity Sc Homology [all charge states]

Scaffold: Version: Scaffold_5.0.1
Modification Metadata Set: 1541 modifications
Source: C:\Program Files\Scaffold 4\parameters\unimod.xml
Comment:
Protein Grouping Strategy: Experiment-wide grouping with protein cluster analysis
Peptide Thresholds: 95.0% minimum
Protein Thresholds: 99.0% minimum and 2 peptides minimum
Peptide FDR: 0.0% (Decoy)
Protein FDR: 0.0% (Decoy)
GO Annotation Source(s):
Pathway Annotation Source(s): Unknown
Alternate ID Source(s):

P7 aniPOND DP583-d												Total Spect control 0901C	Total Spect control BJ1C	Total Spect progeria 0031C	Total Spectrum Count progeria 1671Q
#	Visible?	Starred?	Identified Proteins	Accession Number	Alternate I	Molecular	Protein Gr	Fisher's Ex	Quantitativ	Quantitative Profile	Taxonomy				
1	TRUE	Empty	Histone H4 OS=Hc	H4_HUMAN		11 kDa		< 0.00010		Control low, Patient high	unknown	273	111	535	224
4.1	TRUE	Empty	Plectin OS=Homo	PLEC_HUMAN		532 kDa	TRUE	< 0.00010		Control low, Patient high	unknown	137	193	126	375
5	TRUE	Empty	Prelamin-A/C OS=	LMNA_HUMAN		74 kDa	TRUE	< 0.00010		Control high, Patient low	unknown	181	140	180	76
6	TRUE	Empty	Heterogeneous nu	HNRPU_HUMAN		91 kDa	TRUE	0.0082		[]	unknown	109	75	152	119
7	TRUE	Empty	DNA-dependent p	PRKDC_HUMAN		469 kDa		< 0.00010		Control low, Patient high	unknown	92	39	153	158
9	TRUE	Empty	Heterogeneous nu	HNRPC_HUMAN		34 kDa	TRUE	0.00019		Control low, Patient high	unknown	98	58	168	93
10	TRUE	Empty	Histone H3.1 OS=I	H31_HUMAN		15 kDa		0.51		[]	unknown	42	25	47	32
11	TRUE	Empty	ATP-dependent RI	DHX9_HUMAN		141 kDa		0.0091		[]	unknown	74	53	104	91
13	TRUE	Empty	Heterogeneous nu	ROA2_HUMAN		37 kDa		0.16		[]	unknown	76	65	94	91
14	TRUE	Empty	Heterogeneous nu	HNRPM_HUMAN		78 kDa		0.004		Control low, Patient high	unknown	62	43	94	77
3.1	TRUE	Empty	Histone H1.5 OS=I	H15_HUMAN		23 kDa	TRUE	0.068		[]	unknown	137	18	125	88
15	TRUE	Empty	Non-POU domain-	NONO_HUMAN		54 kDa	TRUE	0.0046		Control low, Patient high	unknown	64	46	85	92
17	TRUE	Empty	Pre-mRNA-proces	PRP8_HUMAN		274 kDa		0.029		[]	unknown	70	14	79	50
19	TRUE	Empty	Splicing factor, prc	SFPQ_HUMAN		76 kDa	TRUE	0.025		[]	unknown	72	39	83	83
21	TRUE	Empty	U5 small nuclear r	U520_HUMAN		245 kDa		0.032		[]	unknown	54	26	70	53
22.1	TRUE	Empty	Interleukin enhan	ILF3_HUMAN		95 kDa	TRUE	0.075		[]	unknown	55	34	72	56
23	TRUE	Empty	Annexin A2 OS=Hc	ANXA2_HUMAN		39 kDa		0.27		[]	unknown	46	74	57	72
25	TRUE	Empty	Heterochromatin	HP1B3_HUMAN		61 kDa		0.033		[]	unknown	55	61	65	40
26	TRUE	Empty	Heterogeneous nu	ROA3_HUMAN		40 kDa	TRUE	0.033		[]	unknown	36	16	50	35
27	TRUE	Empty	60S ribosomal pro	RL4_HUMAN		48 kDa		0.00085		Control high, Patient low	unknown	75	47	55	37
28	TRUE	Empty	Matrin-3 OS=Hom	MATR3_HUMAN		95 kDa		0.0069		[]	unknown	35	20	51	47
24.1	TRUE	Empty	DNA topoisomera	TOP1_HUMAN		91 kDa	TRUE	0.0026		Control low, Patient high	unknown	28	23	53	44
30	TRUE	Empty	Probable ATP-dep	DDX5_HUMAN		69 kDa	TRUE	< 0.00010		Control low, Patient high	unknown	44	42	90	95
31	TRUE	Empty	DNA topoisomera	TOP2B_HUMAN		183 kDa	TRUE	0.00017		Control low, Patient high	unknown	52	22	78	66
32	TRUE	Empty	Nuclear mitotic ap	NUMA1_HUMAN		238 kDa		0.27		[]	unknown	53	20	27	68
33	TRUE	Empty	60S ribosomal pro	RL7A_HUMAN		30 kDa		0.0096		[]	unknown	49	39	37	33
34	TRUE	Empty	Filamin-A OS=Hon	FLNA_HUMAN		281 kDa	TRUE	0.12		[]	unknown	34	53	27	57
35	TRUE	Empty	Tyrosine-protein k	BAZ1B_HUMAN		171 kDa		0.017		[]	unknown	41	6	53	29
36	TRUE	Empty	Poly [ADP-ribose]	PARP1_HUMAN		113 kDa		< 0.00010		Control low, Patient high	unknown	24	16	26	71
37	TRUE	Empty	Heterogeneous nu	HNRPR_HUMAN		71 kDa	TRUE	0.016		[]	unknown	58	55	103	69
39	TRUE	Empty	Heterogeneous nu	HNRPL_HUMAN		64 kDa	TRUE	0.042		[]	unknown	42	20	62	35
40	TRUE	Empty	Structural mainter	SMC1A_HUMAN		143 kDa		0.015		[]	unknown	35	8	45	32
41	TRUE	Empty	Heterogeneous nu	HNRL2_HUMAN		85 kDa		0.024		[]	unknown	32	15	48	32
42	TRUE	Empty	Splicing factor 3B	SF3B3_HUMAN		136 kDa		0.014		[]	unknown	28	17	47	33
43	TRUE	Empty	Splicing factor 3B	SF3B1_HUMAN		146 kDa		0.0011		Control low, Patient high	unknown	32	13	56	36
44	TRUE	Empty	40S ribosomal pro	RS3A_HUMAN		30 kDa		0.006		Control high, Patient low	unknown	35	34	26	24
45	TRUE	Empty	60S ribosomal pro	RL6_HUMAN		33 kDa		0.0021		Control high, Patient low	unknown	34	21	22	12
46	TRUE	Empty	Lamin-B1 OS=Hon	LMNB1_HUMAN		66 kDa	TRUE	< 0.00010		Control low, Patient high	unknown	28	7	41	47
16.1	TRUE	Empty	Heterogeneous nu	ROA1_HUMAN		39 kDa	TRUE	0.5		[]	unknown	49	63	79	51
49	TRUE	Empty	40S ribosomal pro	RS8_HUMAN		24 kDa		0.038		[]	unknown	30	29	28	20
47	TRUE	Empty	Nucleolar RNA hel	DDX21_HUMAN		87 kDa	TRUE	0.15		[]	unknown	32	19	42	31
48	TRUE	Empty	Heterogeneous nu	HNRPK_HUMAN		51 kDa		0.00011		Control low, Patient high	unknown	26	12	44	46
50	TRUE	Empty	Core histone macr	H2AY_HUMAN		40 kDa	TRUE	0.06		[]	unknown	48	40	54	26
51	TRUE	Empty	60S ribosomal pro	RL5_HUMAN		34 kDa		0.23		[]	unknown	27	19	31	14
52	TRUE	Empty	Guanine nucleotid	GBLP_HUMAN		35 kDa		0.44		[]	unknown	17	22	22	21
53	TRUE	Empty	Nucleophosmin O	NPM_HUMAN		33 kDa		< 0.00010		Control high, Patient low	unknown	36	39	22	12
54	TRUE	Empty	RNA-binding prote	RBM14_HUMAN		69 kDa		0.0033		Control low, Patient high	unknown	19	16	41	31
55	TRUE	Empty	60S ribosomal pro	RL7_HUMAN		29 kDa		0.44		[]	unknown	26	20	32	19

18.1	TRUE	Empty	RNA-binding moti	RBMX_HUMAN	42 kDa	TRUE	0.2	[]	unknown	66	47	80	68
29.1	TRUE	Empty	Myosin-9 OS=Hom	MYH9_HUMAN	227 kDa	TRUE	< 0.00010	Control high, Patient low	unknown	33	40	14	25
57	TRUE	Empty	60S ribosomal pro	RL13_HUMAN	24 kDa		0.069	[]	unknown	26	27	20	25
20.1	TRUE	Empty	SWI/SNF-related r	SMCA5_HUMAN	122 kDa	TRUE	0.00046	Control low, Patient high	unknown	42	19	73	47
60	TRUE	Empty	40S ribosomal pro	RS3_HUMAN	27 kDa		0.0043	Control high, Patient low	unknown	25	31	22	15
62	TRUE	Empty	Probable ATP-dep	DDX17_HUMAN	80 kDa	TRUE	< 0.00010	Control low, Patient high	unknown	30	34	59	73
63	TRUE	Empty	Thrombospondin-	TSP1_HUMAN	129 kDa		< 0.00010	Control high, Patient low	unknown	33	56	3	0
64	TRUE	Empty	Nucleolin OS=Hom	NUCL_HUMAN	77 kDa		0.12	[]	unknown	20	12	27	23
65	TRUE	Empty	Heterogeneous nu	HNRDL_HUMAN	46 kDa	TRUE	0.0021	Control low, Patient high	unknown	31	16	53	39
59.1	TRUE	Empty	116 kDa U5 small	U5S1_HUMAN	109 kDa	TRUE	0.0013	Control low, Patient high	unknown	25	5	39	29
67	TRUE	Empty	Heterogeneous nu	HNRPD_HUMAN	38 kDa	TRUE	0.0052	Control low, Patient high	unknown	26	17	48	34
66	TRUE	Empty	Caldesmon OS=Hc	CALD1_HUMAN	93 kDa		0.026	[]	unknown	34	10	31	1
69	TRUE	Empty	Heterogeneous nu	HNRPQ_HUMAN	70 kDa	TRUE	0.33	[]	unknown	55	49	76	54
70	TRUE	Empty	Structural mainter	SMC3_HUMAN	142 kDa		0.048	[]	unknown	22	7	26	25
72	TRUE	Empty	40S ribosomal pro	RS9_HUMAN	23 kDa		0.32	[]	unknown	13	15	18	20
73	TRUE	Empty	Staphylococcal nu	SND1_HUMAN	102 kDa		0.0015	Control high, Patient low	unknown	22	22	16	8
74	TRUE	Empty	Cytoskeleton-asso	CKAP4_HUMAN	66 kDa		0.036	[]	unknown	39	10	35	3
75	TRUE	Empty	ELAV-like protein	ELAV1_HUMAN	36 kDa		0.012	[]	unknown	17	9	33	20
38.1	TRUE	Empty	Chromodomain-he	CHD4_HUMAN	218 kDa	TRUE	0.2	[]	unknown	36	16	33	39
76	TRUE	Empty	DNA topoisomera	TOP2A_HUMAN	174 kDa	TRUE	0.22	[]	unknown	47	8	50	25
78	TRUE	Empty	Interleukin enhan	ILF2_HUMAN	43 kDa		0.08	[]	unknown	15	16	29	22
77	TRUE	Empty	Double-stranded I	DSRAD_HUMAN	136 kDa		0.11	[]	unknown	12	10	18	19
79	TRUE	Empty	Pre-mRNA-proces	PRP19_HUMAN	55 kDa		0.38	[]	unknown	19	7	21	13
80	TRUE	Empty	Thyroid hormone	TR150_HUMAN	109 kDa		0.14	[]	unknown	21	8	27	18
81	TRUE	Empty	Serine/arginine-ric	SRSF1_HUMAN	28 kDa	TRUE	< 0.00010	Control low, Patient high	unknown	7	15	32	31
82	TRUE	Empty	40S ribosomal pro	RS2_HUMAN	31 kDa		0.49	[]	unknown	16	23	22	25
83	TRUE	Empty	60S ribosomal pro	RL3_HUMAN	46 kDa		0.0096	[]	unknown	24	18	15	12
85	TRUE	Empty	Polypyrimidine tra	PTBP1_HUMAN	57 kDa	TRUE	0.15	[]	unknown	25	22	34	34
84	TRUE	Empty	Pre-mRNA-proces	PRP6_HUMAN	107 kDa		0.018	[]	unknown	11	5	20	16
87	TRUE	Empty	Collagen alpha-3(\	CO6A3_HUMAN	344 kDa		< 0.00010	Control high, Patient low	unknown	57	3	1	0
88	TRUE	Empty	Putative pre-mRN	DHX15_HUMAN	91 kDa	TRUE	0.035	[]	unknown	12	5	22	13
89	TRUE	Empty	Heterogeneous nu	ROAA_HUMAN	36 kDa	TRUE	0.073	[]	unknown	16	11	24	22
90	TRUE	Empty	KH domain-contai	KHDR1_HUMAN	48 kDa		0.089	[]	unknown	11	6	10	21
91	TRUE	Empty	Vigilin OS=Homo s	VIGLN_HUMAN	141 kDa		< 0.00010	Control high, Patient low	unknown	28	18	17	1
93	TRUE	Empty	60S ribosomal pro	RL15_HUMAN	24 kDa		0.019	[]	unknown	13	7	3	7
92	TRUE	Empty	Lamin-B2 OS=Hom	LMNB2_HUMAN	68 kDa	TRUE	0.37	[]	unknown	29	5	32	12
94	TRUE	Empty	Antigen KI-67 OS=	KI67_HUMAN	359 kDa		0.16	[]	unknown	19	0	20	11
58.1	TRUE	Empty	Transcription activ	SMCA4_HUMAN	185 kDa	TRUE	0.0032	Control low, Patient high	unknown	23	1	27	28
96	TRUE	Empty	Serine/arginine re	SRRM2_HUMAN	300 kDa		0.22	[]	unknown	17	19	19	15
97	TRUE	Empty	YLP motif-contain	YLPM1_HUMAN	220 kDa		0.24	[]	unknown	16	4	18	12
98	TRUE	Empty	40S ribosomal pro	RS18_HUMAN	18 kDa		0.24	[]	unknown	18	9	14	11
99	TRUE	Empty	Protein DEK OS=H	DEK_HUMAN	43 kDa		0.22	[]	unknown	11	11	19	14
101	TRUE	Empty	40S ribosomal pro	RS16_HUMAN	16 kDa		0.38	[]	unknown	17	9	13	14
100	TRUE	Empty	Heterogeneous nu	ROA0_HUMAN	31 kDa		0.35	[]	unknown	16	8	18	13
103	TRUE	Empty	Zinc finger proteir	ZN638_HUMAN	221 kDa		0.5	[]	unknown	19	4	13	15
104	TRUE	Empty	60S ribosomal pro	RL8_HUMAN	28 kDa		0.096	[]	unknown	8	13	8	7
107	TRUE	Empty	60S ribosomal pro	RL24_HUMAN	18 kDa		0.035	[]	unknown	17	14	11	10
105	TRUE	Empty	Probable ATP-dep	DDX23_HUMAN	96 kDa		0.16	[]	unknown	11	8	15	16
106	TRUE	Empty	RNA-binding prote	RBM25_HUMAN	100 kDa		0.064	[]	unknown	13	3	14	17
108	TRUE	Empty	60S ribosomal pro	RL18_HUMAN	22 kDa		0.51	[]	unknown	11	13	19	10
109	TRUE	Empty	Ribosomal L1 dom	RL1D1_HUMAN	55 kDa		0.5	[]	unknown	13	11	18	9
56.1	TRUE	Empty	40S ribosomal pro	RS4X_HUMAN	30 kDa	TRUE	0.0018	Control high, Patient low	unknown	31	21	18	13
110	TRUE	Empty	Serine/arginine-ric	SRSF7_HUMAN	27 kDa	TRUE	0.36	[]	unknown	17	13	17	14
112	TRUE	Empty	40S ribosomal pro	RS6_HUMAN	29 kDa		0.00031	Control high, Patient low	unknown	12	20	5	7
114	TRUE	Empty	Collagen alpha-1(I	CO1A1_HUMAN	139 kDa		< 0.00010	Control high, Patient low	unknown	23	21	3	0
117	TRUE	Empty	DBIRD complex su	ZN326_HUMAN	66 kDa		0.0041	Control low, Patient high	unknown	13	6	26	20
116	TRUE	Empty	Apoptotic chroma	ACINU_HUMAN	152 kDa		0.31	[]	unknown	11	4	10	12
121	TRUE	Empty	40S ribosomal pro	RS11_HUMAN	18 kDa		0.38	[]	unknown	11	7	8	10
122	TRUE	Empty	Serine/arginine-ric	SRSF3_HUMAN	19 kDa	TRUE	0.48	[]	unknown	13	14	12	21
118	TRUE	Empty	Bcl-2-associated tı	BCLF1_HUMAN	106 kDa		0.27	[]	unknown	13	3	11	13
119	TRUE	Empty	Protein polybromo	PB1_HUMAN	193 kDa		0.2	[]	unknown	7	4	10	9
120	TRUE	Empty	DNA-directed RNA	RPB1_HUMAN	217 kDa		0.0026	Control low, Patient high	unknown	6	3	15	15
115.1	TRUE	Empty	PC4 and SFRS1-int	PSIP1_HUMAN	60 kDa	TRUE	0.0015	Control low, Patient high	unknown	8	4	18	19
123	TRUE	Empty	Core histone macr	H2AW_HUMAN	40 kDa	TRUE	0.016	[]	unknown	13	18	32	27
124	TRUE	Empty	Structural mainter	SMHD1_HUMAN	226 kDa		0.5	[]	unknown	14	3	14	7
125	TRUE	Empty	X-ray repair cross-	XRCC6_HUMAN	70 kDa		0.00033	Control low, Patient high	unknown	6	0	12	17
3.2	TRUE	Empty	Histone H1.2 OS=H	H12_HUMAN	21 kDa	TRUE	0.047	[]	unknown	157	106	159	106
126	TRUE	Empty	Heterogeneous nu	HNRH3_HUMAN	37 kDa	TRUE	0.28	[]	unknown	8	9	12	13
111.1	TRUE	Empty	Sister chromatid c	PDS5B_HUMAN	165 kDa	TRUE	0.11	[]	unknown	9	1	8	12
128	TRUE	Empty	Small nuclear ribo	RSMB_HUMAN (+1)	25 kDa		0.34	[]	unknown	5	9	6	7
129	TRUE	Empty	Splicing factor 3B	SF3B2_HUMAN	100 kDa		0.36	[]	unknown	6	5	6	10
130	TRUE	Empty	60S ribosomal pro	RL13A_HUMAN	24 kDa		0.051	[]	unknown	9	13	10	4
131	TRUE	Empty	Cell division cycle	CDC5L_HUMAN	92 kDa		0.056	[]	unknown	13	4	17	16
133	TRUE	Empty	Transformer-2 prc	TRA2B_HUMAN	34 kDa		0.34	[]	unknown	6	4	8	7
132	TRUE	Empty	U2 snRNP-associa	SR140_HUMAN	118 kDa		0.0027	Control low, Patient high	unknown	7	1	13	15
134	TRUE	Empty	X-ray repair cross-	XRCC5_HUMAN	83 kDa		< 0.00010	Control low, Patient high	unknown	0	5	12	18
3.3	TRUE	Empty	Histone H1.4 OS=H	H14_HUMAN	22 kDa	TRUE	0.013	[]	unknown	167	108	158	107
136	TRUE	Empty	RuvB-like 2 OS=Hc	RUVB2_HUMAN	51 kDa		0.064	[]	unknown	10	6	16	15
137	TRUE	Empty	40S ribosomal pro	RS13_HUMAN	17 kDa		0.16	[]	unknown	7	7	7	3
138	TRUE	Empty	Serrate RNA effect	SRRT_HUMAN	101 kDa		0.006	Control low, Patient high	unknown	6	1	12	12
8.1	TRUE	Empty	Histone H2A type	H2A1C_HUMAN	14 kDa	TRUE	0.09	[]	unknown	281	116	274	148
141	TRUE	Empty	60S ribosomal pro	RL11_HUMAN	20 kDa		0.0093	[]	unknown	16	12	8	7
140	TRUE	Empty	60S ribosomal pro	RL10_HUMAN	25 kDa		0.53	[]	unknown	7	3	5	6
142	TRUE	Empty	40S ribosomal pro	RS5_HUMAN	23 kDa		0.36	[]	unknown	13	9	15	7
143	TRUE	Empty	High mobility grou	HMGA2_HUMAN	12 kDa		0.017	[]	unknown	18	13	14	5
144	TRUE	Empty	60S acidic ribosom	RLA0_HUMAN	34 kDa		0.018	[]	unknown	18	14	11	9
145	TRUE	Empty	Scaffold attachme	SAFB1_HUMAN	103 kDa	TRUE	0.001	Control low, Patient high	unknown	12	9	29	25
147	TRUE	Empty	RNA-binding prote	RBM39_HUMAN	59 kDa		0.019	[]	unknown	9	4	16	15
146	TRUE	Empty	Pinin OS=Homo sa	PININ_HUMAN	82 kDa	TRUE	0.092	[]	unknown	12	3	15	13
148	TRUE	Empty	60S ribosomal pro	RL10A_HUMAN	25 kDa		0.2	[]	unknown	16	8	10	11
149	TRUE	Empty	60S ribosomal pro	RL27_HUMAN	16 kDa		0.16	[]	unknown	8	7	7	4
150	TRUE	Empty	Ribosome-binding	RRBP1_HUMAN	152 kDa		< 0.00010	Control high, Patient low	unknown	17	15	4	0
151	TRUE	Empty	60S ribosomal pro	RL29_HUMAN	18 kDa		0.31	[]	unknown	11	9	8	11
139.1	TRUE	Empty	Spectrin beta chai	SPTB2_HUMAN	275 kDa	TRUE	0.094	[]	unknown	8	5	2	23
113.1	TRUE	Empty	Eukaryotic initiatic	IF4A3_HUMAN	47 kDa	TRUE	0.089	[]	unknown	10	7	13	18
152	TRUE	Empty	Activity-depender	ADNP_HUMAN	124 kDa		0.46	[]	unknown	15	4	11	13
153	TRUE	Empty	FACT complex sub	SP16H_HUMAN	120 kDa		< 0.00010	Control low, Patient high	unknown	5	0	9	30
154	TRUE	Empty	Coronin-1C OS=Hc	COR1C_HUMAN	53 kDa		0.073	[]	unknown	11	12	3	13
155	TRUE	Empty	Scaffold attachme	SAFB2_HUMAN	107 kDa	TRUE	0.0007	Control low, Patient high	unknown	9	9	25	25
156	TRUE	Empty	Serine/arginine-ric	SRSF6_HUMAN	40 kDa	TRUE	0.17	[]	unknown	6	11	12	16
157	TRUE	Empty	RNA-binding prote	RALY_HUMAN	32 kDa		0.012	[]	unknown	6	8	16	18
158	TRUE	Empty	Serine/arginine-ric	SRSF9_HUMAN	26 kDa	TRUE	0.38	[]	unknown	10	7	9	14
161	TRUE	Empty	Tumor suppressor	TP53B_HUMAN	214 kDa		0.00078	Control low, Patient high	unknown	4	2	14	13
160	TRUE	Empty	THO complex subu	THOC2_HUMAN	183 kDa		0.012	[]	unknown	6	2	12	12
159	TRUE	Empty	Protein-glutamine	TGM2_HUMAN	77 kDa		< 0.00010	Control high, Patient low	unknown	20	14	0	0
164	TRUE	Empty	60S ribosomal pro	RL12_HUMAN	18 kDa		0.079	[]	unknown	12	15	11	9
167	TRUE	Empty	Small nuclear ribo	SMD3_HUMAN	14 kDa		0.54	[]	unknown	6	9	10	8
165	TRUE	Empty	60S ribosomal pro	RL14_HUMAN	23 kDa		0.21	[]	unknown	9	7	8	5
163	TRUE	Empty	Protein ELYS OS=I	ELYS_HUMAN	253 kDa		0.43	[]	unknown	14	5	20	0

166	TRUE	Empty	WD40 repeat-con	SMU1_HUMAN	58 kDa		0.0042	Control low, Patient high	unknown	3	2	12	9
170	TRUE	Empty	60S ribosomal pro	RL27A_HUMAN	17 kDa		0.0039	Control high, Patient low	unknown	5	13	1	5
169	TRUE	Empty	Elongation factor	EF1A1_HUMAN (+1)	50 kDa		0.31	[]	unknown	3	12	5	17
171	TRUE	Empty	40S ribosomal pro	RS14_HUMAN	16 kDa		0.023	[]	unknown	7	10	4	4
172	TRUE	Empty	Serine/arginine-ri	SRS10_HUMAN	31 kDa		0.52	[]	unknown	9	4	10	6
3.4	TRUE	Empty	Histone H1.3 OS=I	H13_HUMAN	22 kDa	TRUE	0.011	[]	unknown	162	102	148	104
174	TRUE	Empty	60S ribosomal pro	RL23A_HUMAN	18 kDa		0.048	[]	unknown	11	10	8	5
173	TRUE	Empty	60S ribosomal pro	RL18A_HUMAN	21 kDa		0.53	[]	unknown	10	4	10	7
175	TRUE	Empty	Splicing factor 3A	SF3A1_HUMAN	89 kDa		0.036	[]	unknown	8	2	13	11
176	TRUE	Empty	Myb-binding prot	MBB1A_HUMAN	149 kDa		0.069	[]	unknown	14	7	10	4
179	TRUE	Empty	Zinc finger RNA-bi	ZFR_HUMAN	117 kDa		0.47	[]	unknown	10	5	9	7
177	TRUE	Empty	Putative oxidored	GLYR1_HUMAN	61 kDa		0.45	[]	unknown	14	3	14	4
178	TRUE	Empty	Putative ribosoma	NOP2_HUMAN	89 kDa		0.27	[]	unknown	9	8	10	5
183	TRUE	Empty	THO complex sub	THOC4_HUMAN	27 kDa		0.032	[]	unknown	6	5	13	13
20.2	TRUE	Empty	Probable global tr	SMCA1_HUMAN	123 kDa	TRUE	0.11	[]	unknown	34	14	52	20
181	TRUE	Empty	SWI/SNF complex	SMRC1_HUMAN	123 kDa	TRUE	0.079	[]	unknown	11	7	16	17
182	TRUE	Empty	U4/U6.U5 tri-snRI	SNUT1_HUMAN	90 kDa		0.043	[]	unknown	8	3	18	7
180	TRUE	Empty	RuvB-like 1 OS=Hc	RUVB1_HUMAN	50 kDa		0.074	[]	unknown	8	3	16	7
68.1	TRUE	Empty	Heat shock cogna	HSP7C_HUMAN	71 kDa	TRUE	0.09	[]	unknown	9	13	15	23
185	TRUE	Empty	Metastasis-associ	MTA2_HUMAN	75 kDa	TRUE	0.43	[]	unknown	10	6	11	10
187	TRUE	Empty	Nucleolar transcri	UBF1_HUMAN	89 kDa		0.31	[]	unknown	10	2	11	7
184	TRUE	Empty	Cleavage and poly	CPSF1_HUMAN	161 kDa		0.085	[]	unknown	5	5	14	7
186	TRUE	Empty	Nucleoprotein TPI	TPR_HUMAN	267 kDa		0.013	[]	unknown	4	3	17	5
189	TRUE	Empty	Nucleolar protein	NOP56_HUMAN	66 kDa		0.23	[]	unknown	5	8	16	5
29.2	TRUE	Empty	Myosin-10 OS=Ho	MYH10_HUMAN	229 kDa	TRUE	0.53	[]	unknown	9	17	5	25
188	TRUE	Empty	Filamin-B OS=Hon	FLNB_HUMAN	278 kDa	TRUE	0.13	[]	unknown	1	18	4	10
190	TRUE	Empty	Nuclease-sensitive	YBOX1_HUMAN	36 kDa		0.33	[]	unknown	12	4	6	9
195	TRUE	Empty	40S ribosomal pro	RSSA_HUMAN	33 kDa		0.34	[]	unknown	8	7	8	6
192	TRUE	Empty	Collagen alpha-2(I	CO1A2_HUMAN	129 kDa		0.00038	Control high, Patient low	unknown	10	14	7	0
197	TRUE	Empty	Zinc finger protein	ZN512_HUMAN	65 kDa		0.24	[]	unknown	6	5	11	7
196	TRUE	Empty	E3 ubiquitin-prote	TRIPC_HUMAN	220 kDa		0.34	[]	unknown	8	2	11	4
194	TRUE	Empty	40S ribosomal pro	RS20_HUMAN	13 kDa		0.49	[]	unknown	3	4	3	4
193	TRUE	Empty	Pogo transposable	POGZ_HUMAN	155 kDa		0.0037	Control low, Patient high	unknown	3	0	10	7
61.1	TRUE	Empty	Heterogeneous nu	HNRH1_HUMAN	49 kDa	TRUE	0.032	[]	unknown	23	13	34	29
200	TRUE	Empty	SAFB-like transcri	SLTM_HUMAN	117 kDa		0.54	[]	unknown	7	2	7	3
198	TRUE	Empty	60S ribosomal pro	RL17_HUMAN	21 kDa		0.051	[]	unknown	10	5	5	3
199	TRUE	Empty	60S ribosomal pro	RL26_HUMAN	17 kDa		0.54	[]	unknown	5	4	6	4
201	TRUE	Empty	Transformation/tr	TRRAP_HUMAN	438 kDa		0.034	[]	unknown	4	0	8	6
204	TRUE	Empty	Splicing factor 3A	SF3A3_HUMAN	59 kDa		0.037	[]	unknown	5	2	10	9
205	TRUE	Empty	SURP and G-patch	SUGP2_HUMAN	120 kDa		0.054	[]	unknown	1	2	6	5
202	TRUE	Empty	ATPase family AA	ATD3A_HUMAN	71 kDa		0.085	[]	unknown	5	5	8	13
203	TRUE	Empty	PH-interacting pro	PHIP_HUMAN	207 kDa	TRUE	0.16	[]	unknown	8	1	12	5
209	TRUE	Empty	U1 small nuclear r	RU17_HUMAN	52 kDa		0.22	[]	unknown	5	2	5	8
208	TRUE	Empty	Pre-mRNA-proces	PR40A_HUMAN	109 kDa		0.034	[]	unknown	3	1	8	6
213	TRUE	Empty	Protein Red OS=H	RED_HUMAN	66 kDa		0.0042	Control low, Patient high	unknown	4	1	8	13
214	TRUE	Empty	SWI/SNF complex	SMRC2_HUMAN	133 kDa	TRUE	0.024	[]	unknown	6	5	13	14
210	TRUE	Empty	DNA replication lic	MCM5_HUMAN	82 kDa		0.49	[]	unknown	7	3	12	1
215	TRUE	Empty	Zinc finger CCCH c	ZCH18_HUMAN	106 kDa		0.23	[]	unknown	3	2	3	7
212	TRUE	Empty	Nipped-B-like prot	NIPBL_HUMAN	316 kDa		0.004	Control low, Patient high	unknown	3	1	10	9
211	TRUE	Empty	Nestin OS=Homo	NEST_HUMAN	177 kDa		< 0.00010	Control low, Patient high	unknown	0	0	0	18
223	TRUE	Empty	40S ribosomal pro	RS26_HUMAN	13 kDa		0.44	[]	unknown	6	6	7	9
221	TRUE	Empty	60S ribosomal pro	RL19_HUMAN	23 kDa		0.075	[]	unknown	7	7	5	3
220	TRUE	Empty	Regulator of chror	RCC1_HUMAN	45 kDa		0.097	[]	unknown	5	4	13	6
217	TRUE	Empty	LIM domain only	LMO7_HUMAN	193 kDa		0.46	[]	unknown	6	3	5	4
218	TRUE	Empty	Proline-, glutamic	PELP1_HUMAN	120 kDa		0.0014	Control low, Patient high	unknown	3	0	6	13
219	TRUE	Empty	Paraspeckle comp	PSPC1_HUMAN	59 kDa		< 0.00010	Control low, Patient high	unknown	1	0	14	11
222	TRUE	Empty	U4/U6.U5 tri-snRI	SNUT2_HUMAN	65 kDa		0.15	[]	unknown	5	3	10	6
216	TRUE	Empty	Neuroblast differe	AHNK_HUMAN	629 kDa		0.55	[]	unknown	4	4	0	11
228	TRUE	Empty	Histone H2A.Z OS	H2AZ_HUMAN	14 kDa	TRUE	0.18	[]	unknown	119	84	131	129
127.1	TRUE	Empty	Lamina-associated	LAP2B_HUMAN	51 kDa	TRUE	0.38	[]	unknown	21	5	13	14
229	TRUE	Empty	Beta-catenin-like	CTBL1_HUMAN	65 kDa		0.24	[]	unknown	3	0	5	2
226	TRUE	Empty	60S ribosomal pro	RL21_HUMAN	19 kDa		0.21	[]	unknown	5	6	7	1
224	TRUE	Empty	Insulin-like growtl	IF2B1_HUMAN	63 kDa	TRUE	0.21	[]	unknown	10	5	10	2
227	TRUE	Empty	60S ribosomal pro	RL35_HUMAN	15 kDa		0.37	[]	unknown	4	6	7	2
225	TRUE	Empty	Basement membr	PGBM_HUMAN	469 kDa		< 0.00010	Control high, Patient low	unknown	2	20	0	0
95.1	TRUE	Empty	ATP-dependent RI	DDX3X_HUMAN	73 kDa	TRUE	< 0.00010	Control low, Patient high	unknown	3	2	21	32
231	TRUE	Empty	40S ribosomal pro	RS25_HUMAN	14 kDa		0.56	[]	unknown	4	6	6	6
230	TRUE	Empty	rRNA 2'-O-methyl	FBRL_HUMAN	34 kDa		0.051	[]	unknown	5	5	2	2
237	TRUE	Empty	40S ribosomal pro	RS23_HUMAN	16 kDa		0.35	[]	unknown	7	5	5	6
238	TRUE	Empty	40S ribosomal pro	RS29_HUMAN	7 kDa		0.21	[]	unknown	7	5	3	6
233	TRUE	Empty	DNA replication lic	MCM3_HUMAN	91 kDa		0.05	[]	unknown	3	1	9	4
236	TRUE	Empty	Protein RRP5 hom	RRP5_HUMAN	209 kDa		0.14	[]	unknown	4	5	5	0
235	TRUE	Empty	DNA-directed RNA	RPB2_HUMAN	134 kDa		0.072	[]	unknown	4	0	5	7
234	TRUE	Empty	60S ribosomal pro	RL28_HUMAN	16 kDa		0.058	[]	unknown	5	6	3	2
232	TRUE	Empty	Integrator comple	INT1_HUMAN	244 kDa		0.07	[]	unknown	6	1	9	8
244	TRUE	Empty	40S ribosomal pro	RS7_HUMAN	22 kDa		0.0029	Control high, Patient low	unknown	9	8	4	1
242	TRUE	Empty	40S ribosomal pro	RS15A_HUMAN	15 kDa		0.53	[]	unknown	4	3	5	4
241	TRUE	Empty	RNA-binding prote	RB12B_HUMAN	118 kDa		0.15	[]	unknown	3	1	8	2
239	TRUE	Empty	Probable ATP-dep	DDX46_HUMAN	117 kDa		0.33	[]	unknown	3	3	4	6
240	TRUE	Empty	Pleiotropic regula	PLRG1_HUMAN	57 kDa		0.08	[]	unknown	6	0	9	6
243	TRUE	Empty	40S ribosomal pro	RS24_HUMAN	15 kDa		0.57	[]	unknown	7	5	9	5
68.2	TRUE	Empty	78 kDa glucose-re	GRP78_HUMAN	72 kDa	TRUE	0.28	[]	unknown	9	4	5	6
135.1	TRUE	Empty	Histone-binding p	RBBP4_HUMAN	48 kDa	TRUE	0.55	[]	unknown	9	7	8	11
245	TRUE	Empty	ATP-dependent RI	DDX1_HUMAN	82 kDa		0.055	[]	unknown	8	8	5	4
250	TRUE	Empty	FACT complex sub	SSRP1_HUMAN	81 kDa		0.0052	Control low, Patient high	unknown	1	1	5	9
248	TRUE	Empty	Nuclear pore com	NU205_HUMAN	228 kDa		0.2	[]	unknown	5	1	10	2
246	TRUE	Empty	Guanine nucleotid	GNL3_HUMAN	62 kDa		0.051	[]	unknown	4	6	2	2
249	TRUE	Empty	Putative RNA-binc	RBM15_HUMAN	107 kDa		0.2	[]	unknown	4	0	5	4
247	TRUE	Empty	Nucleolar protein	NOP58_HUMAN	60 kDa		0.47	[]	unknown	3	6	11	1
255	TRUE	Empty	60S ribosomal pro	RL22_HUMAN	15 kDa		0.15	[]	unknown	6	6	5	3
259	TRUE	Empty	DNA damage-bind	DDB1_HUMAN	127 kDa		0.45	[]	unknown	1	2	3	2
257	TRUE	Empty	SNW domain-cont	SNW1_HUMAN	61 kDa		0.31	[]	unknown	3	2	6	3
252	TRUE	Empty	Chromatin target	CHTOP_HUMAN	26 kDa		0.28	[]	unknown	5	5	5	3
253	TRUE	Empty	Filamin-C OS=Hon	FLNC_HUMAN	291 kDa	TRUE	< 0.00010	Control high, Patient low	unknown	8	21	0	2
256	TRUE	Empty	tRNA-splicing liga	RTCB_HUMAN	55 kDa		0.58	[]	unknown	4	3	4	4
258	TRUE	Empty	Transcription elon	SPT6H_HUMAN	199 kDa		0.05	[]	unknown	4	0	8	5
254	TRUE	Empty	HEAT repeat-cont	HEAT1_HUMAN	242 kDa		0.49	[]	unknown	3	4	7	0
191.1	TRUE	Empty	E3 SUMO-protein	RBP2_HUMAN	358 kDa	TRUE	0.12	[]	unknown	7	4	20	1
206.1	TRUE	Empty	Metastasis-associ	MTA1_HUMAN	81 kDa	TRUE	0.36	[]	unknown	5	6	11	5
269	TRUE	Empty	Transformer-2 pro	TRA2A_HUMAN	33 kDa		0.045	[]	unknown	3	2	10	5
270	TRUE	Empty	Hornerin OS=Hom	HORN_HUMAN	282 kDa		0.28	[]	unknown	4	6	2	6
265	TRUE	Empty	DNA replication lic	MCM6_HUMAN	93 kDa		0.11	[]	unknown	5	1	7	7
267	TRUE	Empty	60S ribosomal pro	RL23_HUMAN	15 kDa		0.15	[]	unknown	5	7	5	3
266	TRUE	Empty	PHD finger protei	PHF14_HUMAN	100 kDa		0.38	[]	unknown	7	1	8	4
262	TRUE	Empty	Centromere prote	CENPV_HUMAN	30 kDa		0.21	[]	unknown	7	5	8	1
263	TRUE	Empty	Histone-lysine N-r	EHMT1_HUMAN	141 kDa		0.2	[]	unknown	2	0	5	1
268	TRUE	Empty	Phenylalanine--tR	SYFB_HUMAN	66 kDa		0.12	[]	unknown	5	2	1	2
264	TRUE	Empty	High mobility grou	HMGA1_HUMAN	12 kDa		0.013	[]	unknown	12	5	5	2
261	TRUE	Empty	Bromodomain adj	BAZ1A_HUMAN	179 kDa		0.05	[]	unknown	4	0	7	6

275	TRUE	Empty	Nucleolar GTP-bin	NOG1_HUMAN	74 kDa		0.098	[]	unknown	3	8	3	3
274	TRUE	Empty	ATP-dependent RI	DDX50_HUMAN	83 kDa	TRUE	0.019	[]	unknown	13	8	8	3
272	TRUE	Empty	Actin-like protein	ACL6A_HUMAN	47 kDa		0.47	[]	unknown	5	3	4	4
281	TRUE	Empty	General transcript	GTF2I_HUMAN	112 kDa		0.2	[]	unknown	0	4	2	7
280	TRUE	Empty	E3 ubiquitin-prote	UHRF1_HUMAN	90 kDa		0.4	[]	unknown	4	2	3	2
277	TRUE	Empty	Polymerase delta-	PDIP3_HUMAN	46 kDa		0.44	[]	unknown	7	4	8	3
273	TRUE	Empty	Chromodomain-he	CHD2_HUMAN	211 kDa	TRUE	0.091	[]	unknown	4	1	9	4
278	TRUE	Empty	Protein SON OS=H	SON_HUMAN	264 kDa		0.23	[]	unknown	2	3	3	7
276	TRUE	Empty	Proliferating cell n	PCNA_HUMAN	29 kDa		0.39	[]	unknown	4	3	5	1
279	TRUE	Empty	General transcript	TF3C1_HUMAN	239 kDa		0.014	[]	unknown	2	0	5	7
283	TRUE	Empty	pre-rRNA processi	SPB1_HUMAN	97 kDa		0.54	[]	unknown	3	6	8	2
282	TRUE	Empty	40S ribosomal pro	RS19_HUMAN	16 kDa		0.49	[]	unknown	2	5	5	2
288	TRUE	Empty	Small ubiquitin-rel	SUMO1_HUMAN	12 kDa		0.57	[]	unknown	2	0	2	1
285	TRUE	Empty	60S ribosomal pro	RL30_HUMAN	13 kDa		0.031	[]	unknown	7	8	5	2
287	TRUE	Empty	Peptidyl-prolyl cis-	PPIG_HUMAN	89 kDa		0.37	[]	unknown	4	0	5	2
284	TRUE	Empty	Insulin-like growth	IF2B3_HUMAN	64 kDa	TRUE	0.16	[]	unknown	12	5	7	6
286	TRUE	Empty	Enhancer of rudim	ERH_HUMAN	12 kDa		0.4	[]	unknown	2	3	3	5
296	TRUE	Empty	60S ribosomal pro	RL36A_HUMAN (+1)	12 kDa		0.28	[]	unknown	3	2	1	2
292	TRUE	Empty	Nucleolar and coil	NOLC1_HUMAN	74 kDa		0.23	[]	unknown	3	2	8	2
289	TRUE	Empty	Heterogeneous nu	HNRL1_HUMAN	96 kDa	TRUE	0.5	[]	unknown	5	1	4	2
290	TRUE	Empty	Gamma-interferon	IF16_HUMAN	88 kDa		0.023	[]	unknown	3	0	11	2
293	TRUE	Empty	Transcriptional re	P66A_HUMAN	68 kDa	TRUE	0.43	[]	unknown	7	4	9	6
295	TRUE	Empty	60S ribosomal pro	RL35A_HUMAN	13 kDa		0.29	[]	unknown	3	4	3	2
294	TRUE	Empty	THO complex subu	THOC1_HUMAN	76 kDa		0.0071	[]	unknown	0	0	3	5
291	TRUE	Empty	Midasin OS=Homo	MDN1_HUMAN	633 kDa		0.0071	[]	unknown	0	0	2	6
303	TRUE	Empty	Plasminogen activ	PAIRB_HUMAN	45 kDa		0.49	[]	unknown	6	1	3	4
304	TRUE	Empty	Ras GTPase-activa	G3BP2_HUMAN	54 kDa		0.6	[]	unknown	5	1	2	5
299	TRUE	Empty	Serine/threonine-	PRP4B_HUMAN	117 kDa		0.31	[]	unknown	5	0	5	4
300	TRUE	Empty	60S ribosomal pro	RL36_HUMAN	12 kDa		0.38	[]	unknown	3	5	3	4
297	TRUE	Empty	Transcriptional re	ATRX_HUMAN	283 kDa		0.26	[]	unknown	3	0	1	0
298	TRUE	Empty	Leucine-rich repea	LRC59_HUMAN	35 kDa		0.29	[]	unknown	3	5	2	4
302	TRUE	Empty	40S ribosomal pro	RS30_HUMAN	7 kDa		0.5	[]	unknown	4	2	4	2
301	TRUE	Empty	Spectrin alpha cha	SPTN1_HUMAN	285 kDa		0.014	[]	unknown	0	2	0	12
308	TRUE	Empty	U4/U6 small nucle	PRP4_HUMAN	58 kDa		0.2	[]	unknown	3	1	6	3
312	TRUE	Empty	RNA-binding prote	RNPS1_HUMAN	34 kDa		0.5	[]	unknown	3	2	5	2
102.1	TRUE	Empty	ADP/ATP transloc	ADT3_HUMAN	33 kDa	TRUE	0.01	[]	unknown	7	6	17	16
305	TRUE	Empty	Caprin-1 OS=Hom	CAPR1_HUMAN	78 kDa		0.15	[]	unknown	2	2	4	6
311	TRUE	Empty	Transcription inte	TIF1B_HUMAN	89 kDa		0.1	[]	unknown	3	1	6	5
306	TRUE	Empty	DNA ligase 3 OS=H	DNLI3_HUMAN	113 kDa		0.37	[]	unknown	8	1	4	4
309	TRUE	Empty	Double-strand-bre	RAD21_HUMAN	72 kDa		0.17	[]	unknown	3	0	6	2
310	TRUE	Empty	Helicase SRCAP OS	SRCAP_HUMAN	344 kDa		0.58	[]	unknown	3	0	3	1
307	TRUE	Empty	Insulin-like growth	IF2B2_HUMAN	66 kDa	TRUE	0.12	[]	unknown	12	3	9	1
314	TRUE	Empty	DNA (cytosine-5)-	DNMT1_HUMAN	183 kDa		0.0068	[]	unknown	1	0	5	6
320	TRUE	Empty	Phosphate carrier	MPCP_HUMAN	40 kDa		0.58	[]	unknown	2	1	1	2
316	TRUE	Empty	Unconventional m	MYO1C_HUMAN	122 kDa	TRUE	0.037	[]	unknown	11	1	2	3
317	TRUE	Empty	U4/U6 small nucle	PRP31_HUMAN	55 kDa		0.0071	[]	unknown	0	0	4	4
313	TRUE	Empty	Chromodomain-he	CHD1_HUMAN	197 kDa	TRUE	0.0011	Control low, Patient high	unknown	0	0	7	4
315	TRUE	Empty	Histone H1.0 OS=H	H10_HUMAN	21 kDa		0.00043	Control high, Patient low	unknown	6	4	0	0
318	TRUE	Empty	U5 small nuclear r	SNR40_HUMAN	39 kDa		0.34	[]	unknown	2	1	3	3
319	TRUE	Empty	Small subunit proc	UTP20_HUMAN	318 kDa		0.041	[]	unknown	4	2	1	0
325	TRUE	Empty	Mediator of DNA	MDC1_HUMAN	227 kDa		0.13	[]	unknown	7	1	3	1
323	TRUE	Empty	Probable ATP-dep	DDX27_HUMAN	90 kDa		0.051	[]	unknown	2	8	3	1
324	TRUE	Empty	Histone H1x OS=H	H1X_HUMAN	22 kDa		0.025	[]	unknown	5	5	2	1
332	TRUE	Empty	Nuclear receptor	NCOA5_HUMAN	66 kDa		0.17	[]	unknown	2	3	1	1
331	TRUE	Empty	5'-3' exoribonucle	XRN2_HUMAN	109 kDa		0.6	[]	unknown	3	2	3	3
322	TRUE	Empty	Parafibromin OS=H	CDC73_HUMAN	61 kDa		0.2	[]	unknown	3	1	5	4
326	TRUE	Empty	N-acetyltransferas	NAT10_HUMAN	116 kDa		0.4	[]	unknown	2	4	3	2
330	TRUE	Empty	Transcription elon	SPT5H_HUMAN	121 kDa		0.57	[]	unknown	2	0	3	0
328	TRUE	Empty	Superkiller viralici	SK2L2_HUMAN	118 kDa		0.13	[]	unknown	2	0	3	4
329	TRUE	Empty	Bifunctional gluta	SYEP_HUMAN	171 kDa		0.55	[]	unknown	0	4	0	4
327	TRUE	Empty	40S ribosomal pro	RS12_HUMAN	15 kDa		0.19	[]	unknown	3	4	1	3
321	TRUE	Empty	Bromodomain adj	BAZ2A_HUMAN	211 kDa		0.02	[]	unknown	1	0	4	5
102.2	TRUE	Empty	ADP/ATP transloc	ADT2_HUMAN	33 kDa	TRUE	0.0012	Control low, Patient high	unknown	4	4	14	16
341	TRUE	Empty	Drebrin OS=Homo	DREB_HUMAN	71 kDa		0.51	[]	unknown	2	4	2	6
333	TRUE	Empty	Chromobox protei	CBX3_HUMAN	21 kDa	TRUE	0.17	[]	unknown	4	1	6	5
337	TRUE	Empty	Fragile X mental r	FXR1_HUMAN	70 kDa	TRUE	0.53	[]	unknown	7	0	3	6
338	TRUE	Empty	DNA replication lic	MCM4_HUMAN	97 kDa		0.5	[]	unknown	5	1	5	1
339	TRUE	Empty	Symplekin OS=Ho	SYMPK_HUMAN	141 kDa		0.033	[]	unknown	1	0	5	3
334	TRUE	Empty	Collagen alpha-1(	CO6A1_HUMAN	109 kDa		0.0002	Control high, Patient low	unknown	10	1	0	0
340	TRUE	Empty	DNA replication lic	MCM7_HUMAN	81 kDa		0.4	[]	unknown	3	2	2	2
336	TRUE	Empty	Eukaryotic transla	EIF3A_HUMAN	167 kDa		0.37	[]	unknown	0	1	1	2
335	TRUE	Empty	H/ACA ribonucleo	DKC1_HUMAN	58 kDa		0.057	[]	unknown	3	4	2	0
3.5	TRUE	Empty	Histone H1t OS=H	H1T_HUMAN	22 kDa	TRUE	0.47	[]	unknown	29	26	28	27
71.1	TRUE	Empty	Polyadenylate-bin	PABP1_HUMAN	71 kDa	TRUE	0.46	[]	unknown	20	16	20	20
342	TRUE	Empty	Importin subunit b	IMB1_HUMAN	97 kDa		0.38	[]	unknown	6	2	5	2
343	TRUE	Empty	Nuclear pore com	NUP93_HUMAN	93 kDa		0.28	[]	unknown	2	2	8	0
351	TRUE	Empty	Bromodomain-con	BRD1_HUMAN	120 kDa		0.13	[]	unknown	2	0	4	3
345	TRUE	Empty	Zinc finger protein	REQU_HUMAN	44 kDa		0.054	[]	unknown	3	0	5	6
347	TRUE	Empty	40S ribosomal pro	RS17L_HUMAN (+1)	16 kDa		0.39	[]	unknown	2	5	5	1
350	TRUE	Empty	SWI/SNF-related r	SMCE1_HUMAN	47 kDa		0.58	[]	unknown	3	0	3	1
349	TRUE	Empty	Cleavage and poly	CPSF2_HUMAN	88 kDa		0.42	[]	unknown	2	0	2	2
346	TRUE	Empty	Replication factor	RFC4_HUMAN	40 kDa		0.6	[]	unknown	2	3	2	4
348	TRUE	Empty	Transducin beta-li	TBL2_HUMAN	50 kDa		0.057	[]	unknown	4	3	2	0
344	TRUE	Empty	Poly(U)-binding-sp	PUF60_HUMAN	60 kDa		0.4	[]	unknown	5	0	5	3
86.1	TRUE	Empty	Spliceosome RNA	DX39B_HUMAN	49 kDa	TRUE	0.52	[]	unknown	9	13	15	10
356	TRUE	Empty	TAR DNA-binding	TADBP_HUMAN	45 kDa		0.28	[]	unknown	2	2	5	3
38.2	TRUE	Empty	Chromodomain-he	CHD3_HUMAN	227 kDa	TRUE	0.0018	Control low, Patient high	unknown	8	0	13	14
354	TRUE	Empty	Telomere-associat	RIF1_HUMAN	274 kDa		0.6	[]	unknown	4	0	3	2
353	TRUE	Empty	NHP2-like protein	NH2L1_HUMAN	14 kDa		0.55	[]	unknown	3	1	3	1
357	TRUE	Empty	Protein FAM208A	F208A_HUMAN	189 kDa		0.45	[]	unknown	3	0	1	4
355	TRUE	Empty	Splicing factor 3B	SF3B6_HUMAN	15 kDa		0.056	[]	unknown	0	1	4	3
352	TRUE	Empty	Lysine-specific his	KDM1A_HUMAN	93 kDa		0.6	[]	unknown	4	1	2	4
168.1	TRUE	Empty	Histone deacetyla	HDAC2_HUMAN	55 kDa	TRUE	0.44	[]	unknown	6	6	7	9
366	TRUE	Empty	60S ribosomal pro	RL32_HUMAN	16 kDa		0.4	[]	unknown	5	1	4	1
363	TRUE	Empty	60S ribosomal pro	RL31_HUMAN	14 kDa		0.5	[]	unknown	2	4	1	5
359	TRUE	Empty	Transcriptional re	CTCF_HUMAN	83 kDa		0.37	[]	unknown	4	0	6	1
371	TRUE	Empty	Protein kinase C-b	PKCB1_HUMAN	132 kDa		0.34	[]	unknown	3	0	3	3
368	TRUE	Empty	Microtubule-assoc	MAP4_HUMAN	121 kDa		0.0055	Control high, Patient low	unknown	6	3	0	1
369	TRUE	Empty	ATP-dependent RI	DDX54_HUMAN	99 kDa		0.18	[]	unknown	3	3	1	2
367	TRUE	Empty	Zinc finger CCCH	ZC11A_HUMAN	89 kDa		0.17	[]	unknown	3	0	3	5
370	TRUE	Empty	WD repeat-contai	WDR5_HUMAN	37 kDa		0.57	[]	unknown	1	1	2	1
365	TRUE	Empty	WD repeat-contai	WDR36_HUMAN	105 kDa		0.29	[]	unknown	4	3	5	0
362	TRUE	Empty	Nucleolar complex	NOC2L_HUMAN	85 kDa		0.42	[]	unknown	1	1	4	0
361	TRUE	Empty	DNA replication lic	MCM2_HUMAN	102 kDa		0.3	[]	unknown	2	0	2	3
364	TRUE	Empty	Protein transport	S61A1_HUMAN	52 kDa		0.021	[]	unknown	0	5	0	0
360	TRUE	Empty	ATP-dependent RI	DDX24_HUMAN	96 kDa		0.3	[]	unknown	2	0	5	0
251.1	TRUE	Empty	RNA-binding prote	FUS_HUMAN	53 kDa	TRUE	0.036	[]	unknown	1	1	5	5
381	TRUE	Empty	Non-histone chro	HMGN1_HUMAN	11 kDa		0.17	[]	unknown	5	0	1	1
377	TRUE	Empty	Cohesin subunit S	STAG2_HUMAN	141 kDa	TRUE	0.2	[]	unknown	2	0	5	1

374	TRUE	Empty	Mitochondrial inn	IMMT_HUMAN	84 kDa		0.2	[]	unknown	1	1	1	5
376	TRUE	Empty	General transcript	TF3C3_HUMAN	101 kDa		0.092	[]	unknown	1	0	1	5
378	TRUE	Empty	UAP56-interacting	UIF_HUMAN	36 kDa		0.42	[]	unknown	1	3	2	1
379	TRUE	Empty	Small nuclear ribo	RUXE_HUMAN	11 kDa		0.6	[]	unknown	4	2	5	2
375	TRUE	Empty	Nuclear pore com	NU160_HUMAN	162 kDa		0.15	[]	unknown	1	0	5	0
372	TRUE	Empty	Apoptosis inhibito	API5_HUMAN	59 kDa		0.056	[]	unknown	1	0	2	5
373	TRUE	Empty	Eukaryotic transla	EIF3L_HUMAN	67 kDa		0.42	[]	unknown	2	0	1	3
380	TRUE	Empty	SWI/SNF-related r	SNF5_HUMAN	44 kDa		0.045	[]	unknown	0	0	2	3
393	TRUE	Empty	DNA repair protei	XPC_HUMAN	106 kDa		0.34	[]	unknown	3	0	3	3
207.1	TRUE	Empty	Alpha-actinin-1 OS	ACTN1_HUMAN	103 kDa	TRUE	< 0.00010	Control high, Patient low	unknown	4	15	0	1
389	TRUE	Empty	Peptidyl-prolyl cis	PPIB_HUMAN	24 kDa		0.42	[]	unknown	1	1	1	3
390	TRUE	Empty	Pre-mRNA-splicing	PR38A_HUMAN	37 kDa		0.3	[]	unknown	1	1	3	2
387	TRUE	Empty	Pre-mRNA-splicing	RBM22_HUMAN	47 kDa		0.092	[]	unknown	1	0	4	2
8.2	TRUE	Empty	Histone H2A type	H2A2B_HUMAN	14 kDa	TRUE	0.18	[]	unknown	121	79	136	126
394	TRUE	Empty	Small nuclear ribo	SMD1_HUMAN	13 kDa		0.45	[]	unknown	1	2	2	3
388	TRUE	Empty	Zinc finger MYM-t	ZMYM4_HUMAN	173 kDa	TRUE	0.033	[]	unknown	1	0	6	2
384	TRUE	Empty	Coiled-coil domair	CCD86_HUMAN	40 kDa		0.42	[]	unknown	3	1	3	0
386	TRUE	Empty	Nuclear pore com	NU107_HUMAN	106 kDa		0.58	[]	unknown	1	2	3	0
383	TRUE	Empty	Cell cycle and apo	CCAR2_HUMAN	103 kDa		0.2	[]	unknown	2	0	3	3
391	TRUE	Empty	E1A-binding prote	EP400_HUMAN	343 kDa		0.42	[]	unknown	2	0	2	2
392	TRUE	Empty	U2 small nuclear r	RU2A_HUMAN	28 kDa		0.084	[]	unknown	0	0	3	1
395	TRUE	Empty	Polymerase I and	PTRF_HUMAN	43 kDa		0.21	[]	unknown	0	2	0	0
382	TRUE	Empty	Mitotic checkpoin	BUB3_HUMAN	37 kDa		0.045	[]	unknown	0	0	3	2
385	TRUE	Empty	Chromosome aligr	CHAP1_HUMAN	89 kDa		0.084	[]	unknown	0	0	1	3
407	TRUE	Empty	Paired amphipath	SIN3A_HUMAN	145 kDa		0.34	[]	unknown	2	1	4	2
408	TRUE	Empty	Splicing factor U2	U2AF2_HUMAN	54 kDa		0.02	[]	unknown	0	1	4	5
162.1	TRUE	Empty	Serine/threonine-	PP1A_HUMAN	38 kDa	TRUE	0.5	[]	unknown	6	6	6	7
416	TRUE	Empty	Serine/arginine-ri	SRSF5_HUMAN	31 kDa	TRUE	0.2	[]	unknown	1	5	4	8
417	TRUE	Empty	mRNA turnover pr	MRT4_HUMAN	28 kDa		0.57	[]	unknown	0	2	2	1
411	TRUE	Empty	ATP-dependent RI	DDX18_HUMAN	75 kDa		0.27	[]	unknown	2	2	2	0
410	TRUE	Empty	Histone-lysine N-r	NSD2_HUMAN	152 kDa		0.42	[]	unknown	4	0	3	0
403	TRUE	Empty	Small nuclear ribo	SMD2_HUMAN	14 kDa		0.42	[]	unknown	1	3	0	3
400	TRUE	Empty	Protein phosphata	MYPT1_HUMAN	115 kDa		0.0096	[]	unknown	2	4	0	0
414	TRUE	Empty	Polyadenylate-bin	PABP2_HUMAN	33 kDa		0.3	[]	unknown	2	0	3	2
413	TRUE	Empty	Transcription fact	TFAM_HUMAN	29 kDa		0.57	[]	unknown	1	1	1	2
162.2	TRUE	Empty	Serine/threonine-	PP1B_HUMAN	37 kDa	TRUE	0.53	[]	unknown	4	6	6	5
412	TRUE	Empty	Remodeling and s	RSF1_HUMAN	164 kDa		0.084	[]	unknown	0	0	2	2
406	TRUE	Empty	Ras GTPase-activa	G3BP1_HUMAN	52 kDa		0.42	[]	unknown	2	0	2	2
418	TRUE	Empty	Probable rRNA-pr	EBP2_HUMAN	35 kDa		0.63	[]	unknown	0	2	2	0
402	TRUE	Empty	40S ribosomal pro	RS21_HUMAN	9 kDa		0.17	[]	unknown	3	2	2	0
399	TRUE	Empty	Ribosomal biogen	LAS1L_HUMAN	83 kDa		0.3	[]	unknown	0	2	1	4
398	TRUE	Empty	Eukaryotic transla	IF2G_HUMAN	51 kDa		0.58	[]	unknown	1	2	0	3
415	TRUE	Empty	60S ribosomal pro	RL9_HUMAN	22 kDa		0.27	[]	unknown	2	2	2	0
409	TRUE	Empty	Integrator comple	INT3_HUMAN	118 kDa		0.44	[]	unknown	2	0	1	0
405	TRUE	Empty	Zinc finger protei	ZN384_HUMAN	63 kDa		0.025	[]	unknown	0	0	6	0
401	TRUE	Empty	Pescadillo homolo	PESC_HUMAN	68 kDa		0.17	[]	unknown	4	1	2	0
404	TRUE	Empty	Pre-mRNA-splicing	SYF1_HUMAN	100 kDa		0.013	[]	unknown	0	0	5	2
397	TRUE	Empty	ATP-citrate syntha	ACLY_HUMAN	121 kDa		0.013	[]	unknown	0	0	0	7
71.2	TRUE	Empty	Polyadenylate-bin	PABP4_HUMAN	71 kDa	TRUE	0.4	[]	unknown	8	7	9	6
426	TRUE	Empty	Zinc finger MYM-t	ZMYM3_HUMAN	152 kDa	TRUE	0.056	[]	unknown	1	0	4	3
425	TRUE	Empty	U4/U6 small nucle	PRPF3_HUMAN	78 kDa		0.24	[]	unknown	1	0	4	0
2.1	TRUE	Empty	Histone H2B type	H2B1A_HUMAN	14 kDa	TRUE	0.24	[]	unknown	76	38	52	78
427	TRUE	Empty	Cohesin subunit S	STAG1_HUMAN	144 kDa	TRUE	0.43	[]	unknown	3	0	2	0
428	TRUE	Empty	60S ribosomal pro	RL38_HUMAN	8 kDa		0.43	[]	unknown	2	1	1	1
420	TRUE	Empty	Pre-mRNA 3'-end-	FIP1_HUMAN	67 kDa		0.088	[]	unknown	2	0	2	6
422	TRUE	Empty	Phenylalanine--tR	SYFA_HUMAN	58 kDa		0.55	[]	unknown	3	1	4	0
423	TRUE	Empty	Cleavage and poly	CPSF7_HUMAN	52 kDa		0.033	[]	unknown	1	0	3	5
424	TRUE	Empty	Ribosome biogene	RRS1_HUMAN	41 kDa		0.58	[]	unknown	2	1	3	0
419	TRUE	Empty	Copine-3 OS=Hom	CPNE3_HUMAN	60 kDa		0.084	[]	unknown	0	0	1	3
421	TRUE	Empty	Pumilio domain-co	K0020_HUMAN	74 kDa		0.37	[]	unknown	1	0	3	0
12.1	TRUE	Empty	Ubiquitin-40S ribo	RS27A_HUMAN	18 kDa	TRUE	0.46	[]	unknown	81	48	93	55
2.2	TRUE	Empty	Histone H2B type	H2B1J_HUMAN	14 kDa	TRUE	0.31	[]	unknown	145	69	157	106
433	TRUE	Empty	Fragile X mental r	FXR2_HUMAN	74 kDa	TRUE	0.4	[]	unknown	5	0	2	2
441	TRUE	Empty	THO complex sub	THOC3_HUMAN	39 kDa		0.3	[]	unknown	1	1	2	3
442	TRUE	Empty	Zinc finger protei	ZN516_HUMAN	124 kDa		0.025	[]	unknown	0	0	2	4
439	TRUE	Empty	WD repeat-contai	WDR18_HUMAN	47 kDa		0.42	[]	unknown	0	2	1	3
438	TRUE	Empty	Poly(rC)-binding p	PCBP2_HUMAN	39 kDa	TRUE	0.42	[]	unknown	3	3	5	4
444	TRUE	Empty	Nuclear pore com	NU133_HUMAN	129 kDa		0.57	[]	unknown	1	1	3	0
431	TRUE	Empty	AT-rich interactive	ARI1A_HUMAN	242 kDa	TRUE	0.14	[]	unknown	4	0	1	0
440	TRUE	Empty	Eukaryotic transla	EIF3B_HUMAN	92 kDa		0.57	[]	unknown	2	0	1	2
429	TRUE	Empty	Cleavage stimulati	CSTF3_HUMAN	83 kDa		0.42	[]	unknown	1	1	1	3
436	TRUE	Empty	RRP12-like protei	RRP12_HUMAN	144 kDa		0.42	[]	unknown	2	0	4	0
443	TRUE	Empty	Cleavage stimulati	CSTF2_HUMAN	61 kDa		0.56	[]	unknown	1	0	0	2
430	TRUE	Empty	Putative ATP-depe	DHX30_HUMAN	134 kDa		0.056	[]	unknown	1	0	1	6
437	TRUE	Empty	Nesprin-2 OS=Hor	SYNE2_HUMAN	796 kDa		0.56	[]	unknown	0	1	0	2
434	TRUE	Empty	Importin subunit a	IMA1_HUMAN	58 kDa		0.084	[]	unknown	0	0	2	2
435	TRUE	Empty	RNA polymerase I	PAF1_HUMAN	60 kDa		0.42	[]	unknown	2	0	2	2
432	TRUE	Empty	Zinc finger CCCH d	ZC3H4_HUMAN	140 kDa		0.045	[]	unknown	0	0	0	5
461	TRUE	Empty	Calcium homeosta	CHERP_HUMAN	104 kDa		0.056	[]	unknown	1	0	3	4
458	TRUE	Empty	Centrosomal prot	CE170_HUMAN	175 kDa		0.58	[]	unknown	1	2	0	3
460	TRUE	Empty	Kinesin-like protei	KI20A_HUMAN	100 kDa		0.63	[]	unknown	2	0	0	2
457	TRUE	Empty	Microtubule-assoc	MAP1B_HUMAN	271 kDa	TRUE	0.092	[]	unknown	0	1	0	6
450	TRUE	Empty	60S ribosomal pro	RL7L_HUMAN	29 kDa		0.17	[]	unknown	1	4	2	0
454	TRUE	Empty	THO complex sub	THOC6_HUMAN	38 kDa		0.42	[]	unknown	1	1	2	2
449	TRUE	Empty	Ran GTPase-activa	RAGP1_HUMAN	64 kDa		0.17	[]	unknown	4	1	1	1
452	TRUE	Empty	Zinc finger CCCH d	ZC3HE_HUMAN	83 kDa		0.013	[]	unknown	0	0	1	6
455	TRUE	Empty	Nucleosome-remo	BPTF_HUMAN	338 kDa		0.045	[]	unknown	0	0	1	4
453	TRUE	Empty	Splicing factor 45	SPF45_HUMAN	45 kDa		0.42	[]	unknown	0	2	2	2
459	TRUE	Empty	U1 small nuclear r	SNRPA_HUMAN	31 kDa		0.37	[]	unknown	0	1	0	3
451	TRUE	Empty	Isoleucine--tRNA l	SYIC_HUMAN	145 kDa		0.3	[]	unknown	0	2	0	5
447	TRUE	Empty	Ribosome biogene	BOP1_HUMAN	84 kDa		0.44	[]	unknown	0	2	1	0
448	TRUE	Empty	Luc7-like protein 3	LC7L3_HUMAN	51 kDa		0.045	[]	unknown	0	0	2	3
456	TRUE	Empty	Procollagen C-end	PCOC1_HUMAN	48 kDa		0.098	[]	unknown	3	0	0	0
260.1	TRUE	Empty	Bromodomain-cor	BRD3_HUMAN	80 kDa	TRUE	0.42	[]	unknown	4	2	6	3
486	TRUE	Empty	Major centromere	CENPB_HUMAN	65 kDa		0.42	[]	unknown	2	0	4	0
445.1	TRUE	Empty	60S ribosomal pro	RL37A_HUMAN	10 kDa	TRUE	0.077	[]	unknown	2	3	1	0
479	TRUE	Empty	Pre-mRNA-splicing	SPF27_HUMAN	26 kDa		0.42	[]	unknown	1	1	2	2
58.2	TRUE	Empty	Probable global tr	SMCA2_HUMAN	181 kDa	TRUE	0.051	[]	unknown	7	0	12	9
487	TRUE	Empty	Poly(rC)-binding p	PCBP1_HUMAN	37 kDa	TRUE	0.6	[]	unknown	3	3	3	4
477	TRUE	Empty	Constitutive coact	F120A_HUMAN	122 kDa		0.58	[]	unknown	3	0	1	2
465	TRUE	Empty	Nucleoporin NUP5	NUP53_HUMAN	35 kDa		0.58	[]	unknown	2	1	4	0
488	TRUE	Empty	RNA-binding prote	RBM8A_HUMAN	20 kDa		0.63	[]	unknown	2	0	1	1
481	TRUE	Empty	40S ribosomal pro	RS15_HUMAN	17 kDa		0.44	[]	unknown	2	0	1	0
485	TRUE	Empty	Protein Wiz OS=H	WIZ_HUMAN	179 kDa		0.42	[]	unknown	2	0	2	2
484	TRUE	Empty	Crooked neck-like	CRNL1_HUMAN	100 kDa		0.56	[]	unknown	1	0	2	0
478	TRUE	Empty	Host cell factor 1	HCFC1_HUMAN	209 kDa		0.16	[]	unknown	0	0	0	3
467	TRUE	Empty	Probable ATP-dep	DDX6_HUMAN	54 kDa		0.025	[]	unknown	0	0	2	4
471	TRUE	Empty	DNA repair protei	XRCC1_HUMAN	69 kDa		0.37	[]	unknown	1	0	0	3
489	TRUE	Empty	Probable ATP-dep	YTDC2_HUMAN	160 kDa		0.21	[]	unknown	2	0	0	0

464	TRUE	Empty	Chromatin assemb	CAF1B_HUMAN	61 kDa		0.17	[]	unknown	2	3	2	0
476	TRUE	Empty	MKI67 FHA domai	MK67I_HUMAN	34 kDa		0.27	[]	unknown	2	2	2	0
470	TRUE	Empty	Double-stranded	STAU1_HUMAN	63 kDa		0.37	[]	unknown	1	0	2	1
473	TRUE	Empty	Coiled-coil domair	CC71L_HUMAN	26 kDa		0.24	[]	unknown	1	0	3	1
483	TRUE	Empty	Nuclear pore com	NU153_HUMAN	154 kDa		0.37	[]	unknown	0	1	3	0
472	TRUE	Empty	RNA-binding prote	RBM28_HUMAN	86 kDa		0.43	[]	unknown	1	2	2	0
480	TRUE	Empty	U3 small nucleolar	U3IP2_HUMAN	52 kDa		0.44	[]	unknown	0	2	1	0
469	TRUE	Empty	Dolichyl-diphosph	RPN1_HUMAN	69 kDa		0.084	[]	unknown	0	0	3	1
475	TRUE	Empty	Ribosomal RNA pr	RRP1_HUMAN	53 kDa		0.63	[]	unknown	0	2	2	0
474	TRUE	Empty	SWI/SNF-related r	SMRD2_HUMAN	59 kDa	TRUE	0.37	[]	unknown	1	0	0	3
466	TRUE	Empty	mRNA export facto	RAE1L_HUMAN	41 kDa		0.57	[]	unknown	2	0	3	0
482	TRUE	Empty	Cleavage and poly	CPSF3_HUMAN	77 kDa		0.29	[]	unknown	0	0	2	0
468	TRUE	Empty	Nucleoporin NUP:	NU188_HUMAN	196 kDa		0.16	[]	unknown	0	0	3	0
271.1	TRUE	Empty	Moesin OS=Homo	MOES_HUMAN	68 kDa	TRUE	0.081	[]	unknown	5	4	1	3
56.2	TRUE	Empty	40S ribosomal pro	RS4Y1_HUMAN	29 kDa	TRUE	0.092	[]	unknown	12	7	6	7
504	TRUE	Empty	Procollagen-lysine	PLOD1_HUMAN	84 kDa		0.0096	[]	unknown	3	3	0	0
506	TRUE	Empty	Serpin H1 OS=Hom	SERPH_HUMAN	46 kDa		0.27	[]	unknown	4	0	2	0
507	TRUE	Empty	Transducin beta-li	TBL3_HUMAN	89 kDa		0.42	[]	unknown	2	0	3	1
503	TRUE	Empty	Nuclear cap-bindi	NCBP1_HUMAN	92 kDa		0.24	[]	unknown	1	0	2	2
501	TRUE	Empty	Elongation factor	EF1G_HUMAN	50 kDa		0.098	[]	unknown	0	3	0	0
499	TRUE	Empty	Serine/threonine-	PGAM5_HUMAN	32 kDa		0.37	[]	unknown	0	1	1	2
505	TRUE	Empty	SAP domain-conta	SARNP_HUMAN	24 kDa		0.63	[]	unknown	0	2	1	1
509	TRUE	Empty	Cullin-4A OS=Hom	CUL4A_HUMAN	88 kDa		0.63	[]	unknown	2	0	2	0
502	TRUE	Empty	Lysine-specific der	KDM5B_HUMAN	176 kDa		0.37	[]	unknown	1	0	3	0
494	TRUE	Empty	KRR1 small subuni	KRR1_HUMAN	44 kDa		0.045	[]	unknown	0	0	4	1
498	TRUE	Empty	Cell division cycle	CCAR1_HUMAN	133 kDa		0.16	[]	unknown	0	0	0	3
508	TRUE	Empty	Splicing factor U2	U2AF1_HUMAN	28 kDa		0.045	[]	unknown	0	0	2	3
500	TRUE	Empty	Ribosomal RNA pr	RRP1B_HUMAN	84 kDa		0.098	[]	unknown	2	1	0	0
492	TRUE	Empty	Calponin-3 OS=Ho	CNN3_HUMAN	36 kDa		0.37	[]	unknown	0	1	0	3
491	TRUE	Empty	Actin-related prot	ARPC4_HUMAN	20 kDa		0.084	[]	unknown	0	0	2	2
496	TRUE	Empty	Lysine--tRNA ligas	SYK_HUMAN	68 kDa		0.29	[]	unknown	0	0	0	2
497	TRUE	Empty	Nucleolar protein	NOL6_HUMAN	128 kDa		0.21	[]	unknown	2	0	0	0
493	TRUE	Empty	Collagen alpha-2(	CO6A2_HUMAN	109 kDa		0.098	[]	unknown	3	0	0	0
495	TRUE	Empty	DNA-(apurinic or a	APEX1_HUMAN	36 kDa		0.29	[]	unknown	0	0	0	2
490	TRUE	Empty	Arginine--tRNA lig	SYRC_HUMAN	75 kDa		0.16	[]	unknown	0	0	0	3
18.2	TRUE	Empty	RNA binding motif	RMXL1_HUMAN	42 kDa	TRUE	0.37	[]	unknown	32	32	42	37
260.2	TRUE	Empty	Bromodomain-cor	BRD4_HUMAN	152 kDa	TRUE	0.51	[]	unknown	4	2	4	4
518	TRUE	Empty	Signal recognition	SRP14_HUMAN	15 kDa		0.43	[]	unknown	2	1	1	1
520	TRUE	Empty	Methyl-CpG-bindi	MECP2_HUMAN	52 kDa		0.43	[]	unknown	2	1	2	0
517	TRUE	Empty	Replication factor	RFC1_HUMAN	128 kDa		0.63	[]	unknown	2	0	1	1
251.2	TRUE	Empty	TATA-binding prot	RBP56_HUMAN	62 kDa	TRUE	0.013	[]	unknown	0	0	5	5
462.1	TRUE	Empty	ATP-dependent RI	DHX36_HUMAN	115 kDa	TRUE	0.24	[]	unknown	1	0	2	2
521	TRUE	Empty	Replication factor	RFC5_HUMAN	38 kDa		0.37	[]	unknown	1	0	2	1
513	TRUE	Empty	RNA 3'-terminal p	RCL1_HUMAN	41 kDa		0.57	[]	unknown	1	1	3	0
446.1	TRUE	Empty	Tubulin alpha-1A	TBA1A_HUMAN (+3)	50 kDa	TRUE	0.045	[]	unknown	0	0	1	4
522	TRUE	Empty	Serine/arginine-ri	SRSF2_HUMAN	25 kDa		0.084	[]	unknown	0	0	0	4
516	TRUE	Empty	Clathrin heavy cha	CLH1_HUMAN	192 kDa		0.37	[]	unknown	0	1	0	3
511	TRUE	Empty	Nuclear pore com	NU155_HUMAN	155 kDa		0.24	[]	unknown	1	0	4	0
525	TRUE	Empty	Cell division cycle-	CDA7L_HUMAN	52 kDa		0.16	[]	unknown	0	0	2	1
523	TRUE	Empty	WD repeat-contai	WDR82_HUMAN	35 kDa		0.44	[]	unknown	0	2	0	1
463.1	TRUE	Empty	Chromodomain-h	CHD9_HUMAN	326 kDa	TRUE	0.045	[]	unknown	0	0	4	2
519	TRUE	Empty	Nuclear pore com	NUP88_HUMAN	84 kDa		0.16	[]	unknown	0	0	3	0
514	TRUE	Empty	Pre-mRNA-splicing	FL2D_HUMAN	44 kDa		0.63	[]	unknown	2	0	2	0
524	TRUE	Empty	Ribosome product	RPF2_HUMAN	36 kDa		0.21	[]	unknown	0	2	0	0
515	TRUE	Empty	Coilin OS=Homo s	COIL_HUMAN	63 kDa		0.29	[]	unknown	0	0	2	0
512	TRUE	Empty	Methyl-CpG-bindi	MBD2_HUMAN	43 kDa		0.16	[]	unknown	0	0	3	0
2.3	TRUE	Empty	Histone H2B type	H2B3B_HUMAN	14 kDa	TRUE	0.13	[]	unknown	113	58	122	103
358.1	TRUE	Empty	Small ubiquitin-rel	SUMO2_HUMAN	11 kDa	TRUE	0.07	[]	unknown	4	4	3	0
396.1	TRUE	Empty	40S ribosomal pro	RS27_HUMAN	9 kDa	TRUE	0.42	[]	unknown	2	2	1	2
162.3	TRUE	Empty	Serine/threonine-	PP1G_HUMAN	37 kDa	TRUE	0.54	[]	unknown	5	4	4	6
548	TRUE	Empty	Polypyrimidine tra	PTBP3_HUMAN	60 kDa	TRUE	0.0011	Control low, Patient high	unknown	0	0	7	11
535	TRUE	Empty	DNA methyltransf	DMAP1_HUMAN	53 kDa		0.63	[]	unknown	2	0	1	1
536	TRUE	Empty	Protein FAM98A	FA98A_HUMAN	55 kDa		0.63	[]	unknown	1	1	2	0
546	TRUE	Empty	Zinc finger protein	ZN687_HUMAN	130 kDa		0.63	[]	unknown	2	0	2	0
545	TRUE	Empty	Glutamate dehydr	DHE3_HUMAN (+1)	61 kDa		0.37	[]	unknown	1	0	1	2
544	TRUE	Empty	Transcription fact	AP2C_HUMAN	49 kDa		0.084	[]	unknown	0	0	4	0
543	TRUE	Empty	COUP transcriptio	COT2_HUMAN	46 kDa		0.045	[]	unknown	3	1	0	0
539	TRUE	Empty	Zinc finger homeo	ZFHX3_HUMAN	404 kDa	TRUE	0.16	[]	unknown	0	0	3	0
534	TRUE	Empty	ATPase family AA	ATAD2_HUMAN	159 kDa		0.045	[]	unknown	4	0	0	0
533	TRUE	Empty	Heterogeneous nu	HNRLL_HUMAN	60 kDa	TRUE	0.56	[]	unknown	1	0	2	0
542	TRUE	Empty	Nucleolar comple	NOC4L_HUMAN	58 kDa		0.56	[]	unknown	1	0	2	0
527	TRUE	Empty	Emerin OS=Homo	EMD_HUMAN	29 kDa		0.045	[]	unknown	3	1	0	0
540	TRUE	Empty	Actin-related prot	ARP2_HUMAN	45 kDa		0.16	[]	unknown	0	0	1	2
528	TRUE	Empty	WW domain-bindi	WBP11_HUMAN	70 kDa		0.084	[]	unknown	0	0	0	4
529	TRUE	Empty	Protein MAK16 ho	MAK16_HUMAN	35 kDa		0.29	[]	unknown	0	0	2	0
537	TRUE	Empty	E3 ubiquitin-prote	RING2_HUMAN	38 kDa	TRUE	0.29	[]	unknown	0	0	2	0
538	TRUE	Empty	Leucine--tRNA liga	SYLC_HUMAN	134 kDa		0.29	[]	unknown	0	0	0	2
547	TRUE	Empty	General transcript	TF3C4_HUMAN	92 kDa		0.29	[]	unknown	0	0	0	2
532	TRUE	Empty	SPATS2-like protei	SPS2L_HUMAN	62 kDa		0.21	[]	unknown	2	0	0	0
541	TRUE	Empty	Matrix-remodeling	MXRA5_HUMAN	312 kDa		0.29	[]	unknown	0	0	2	0
530	TRUE	Empty	Putative RNA-bind	LC7L2_HUMAN	47 kDa		0.29	[]	unknown	0	0	0	2
526	TRUE	Empty	Cysteine-rich secr	CRLD1_HUMAN	57 kDa		0.16	[]	unknown	0	0	3	0
531	TRUE	Empty	PDZ and LIM dom	PDLI4_HUMAN	35 kDa		0.21	[]	unknown	0	2	0	0
2.4	TRUE	Empty	Histone H2B type	H2B1D_HUMAN	14 kDa	TRUE	0.041	[]	unknown	184	84	225	136
2.5	TRUE	Empty	Histone H2B type	H2B1O_HUMAN	14 kDa	TRUE	0.28	[]	unknown	145	70	158	108
12.2	TRUE	Empty	Ubiquitin-60S ribo	RL40_HUMAN	15 kDa	TRUE	0.44	[]	unknown	80	48	91	55
61.2	TRUE	Empty	Heterogeneous nu	HNRH2_HUMAN	49 kDa	TRUE	0.026	[]	unknown	9	7	18	15
86.2	TRUE	Empty	ATP-dependent RI	DX39A_HUMAN	49 kDa	TRUE	0.15	[]	unknown	4	10	13	10
95.2	TRUE	Empty	ATP-dependent RI	DDX3Y_HUMAN	73 kDa	TRUE	< 0.00010	Control low, Patient high	unknown	3	1	15	19
135.2	TRUE	Empty	Histone-binding p	RBBP7_HUMAN	48 kDa	TRUE	0.53	[]	unknown	6	1	3	6
68.3	TRUE	Empty	Heat shock-relate	HSP72_HUMAN	70 kDa	TRUE	0.015	[]	unknown	6	0	6	11
569	TRUE	Empty	Transcriptional re	P66B_HUMAN	65 kDa	TRUE	0.63	[]	unknown	4	0	0	2
206.2	TRUE	Empty	Metastasis-associ	MTA3_HUMAN	68 kDa	TRUE	0.088	[]	unknown	0	2	6	4
29.3	TRUE	Empty	Myosin-11 OS=Ho	MYH11_HUMAN	227 kDa	TRUE	0.4	[]	unknown	6	9	4	0
554	TRUE	Empty	REST corepressor	RCOR1_HUMAN	53 kDa	TRUE	0.16	[]	unknown	0	0	1	2
565	TRUE	Empty	Lysozyme C OS=H	LYSC_HUMAN	17 kDa		0.098	[]	unknown	2	1	0	0
560	TRUE	Empty	Trifunctional enzy	ECHB_HUMAN	51 kDa		0.56	[]	unknown	0	1	2	0
557	TRUE	Empty	Integrator comple	INT6_HUMAN	100 kDa		0.16	[]	unknown	0	0	2	1
562	TRUE	Empty	Nucleolar GTP-bin	NOG2_HUMAN	84 kDa		0.56	[]	unknown	1	0	2	0
510.1	TRUE	Empty	SWI/SNF-related r	SMRD1_HUMAN	58 kDa	TRUE	0.084	[]	unknown	0	0	1	3
567	TRUE	Empty	Alpha-internexin	AINX_HUMAN	55 kDa	TRUE	< 0.00010	Control high, Patient low	unknown	0	95	0	29
568	TRUE	Empty	Glutamine--fructo	GFPT1_HUMAN	79 kDa		0.21	[]	unknown	2	0	0	0
561	TRUE	Empty	Eukaryotic transla	IF6_HUMAN	27 kDa		0.29	[]	unknown	0	0	2	0
555	TRUE	Empty	Nucleoporin p54	NUP54_HUMAN	55 kDa		0.29	[]	unknown	0	0	2	0
553	TRUE	Empty	Cold-inducible RN	CIRBP_HUMAN	19 kDa		0.29	[]	unknown	0	0	0	2
563	TRUE	Empty	Kinectin OS=Homo	KTN1_HUMAN	156 kDa		0.29	[]	unknown	0	0	0	2
559	TRUE	Empty	Glyceraldehyde-3-	G3P_HUMAN	36 kDa		0.21	[]	unknown	0	2	0	0
566	TRUE	Empty	Nuclear respirator	NRF1_HUMAN	54 kDa		0.29	[]	unknown	0	0	0	2
558	TRUE	Empty	Glycylpeptide N-te	NMT1_HUMAN	57 kDa		0.29	[]	unknown	0	0	0	2

549	TRUE	Empty	Calponin-1 OS=Ho	CNN1_HUMAN	33 kDa		0.098	[]	unknown	0	3	0	0
550	TRUE	Empty	Exosome complex	EXOS6_HUMAN	28 kDa		0.098	[]	unknown	0	3	0	0
551	TRUE	Empty	Gremlin-1 OS=Hor	GREM1_HUMAN	21 kDa		0.21	[]	unknown	2	0	0	0
552	TRUE	Empty	Fructose-bisphosp	ALDOA_HUMAN	39 kDa		0.21	[]	unknown	0	2	0	0
556	TRUE	Empty	General transcript	TF3C5_HUMAN	60 kDa		0.29	[]	unknown	0	0	0	2
564	TRUE	Empty	Bromodomain-cor	BRD2_HUMAN	88 kDa	TRUE	0.16	[]	unknown	0	0	0	3
29.4	TRUE	Empty	Myosin-14 OS=Ho	MYH14_HUMAN	228 kDa	TRUE	0.29	[]	unknown	5	0	4	0
59.2	TRUE	Empty	Elongation factor :	EF2_HUMAN	95 kDa	TRUE	0.46	[]	unknown	1	0	0	0
12.3	TRUE	Empty	Polyubiquitin-B OS	UBB_HUMAN (+1)	26 kDa	TRUE	0.49	[]	unknown	79	47	91	55
8.3	TRUE	Empty	Histone H2A.J OS=	H2AJ_HUMAN	14 kDa	TRUE	0.2	[]	unknown	291	118	286	165
3.6	TRUE	Empty	Histone H1.1 OS=I	H11_HUMAN	22 kDa	TRUE	< 0.00010	Control high, Patient low	unknown	44	44	35	34
207.2	TRUE	Empty	Alpha-actinin-4 OS	ACTN4_HUMAN	105 kDa	TRUE	< 0.00010	Control high, Patient low	unknown	3	13	0	1
61.3	TRUE	Empty	Heterogeneous nu	HNRPF_HUMAN	46 kDa	TRUE	0.38	[]	unknown	7	3	10	5
111.2	TRUE	Empty	Sister chromatid c	PDS5A_HUMAN	151 kDa	TRUE	0.56	[]	unknown	2	0	2	0
113.2	TRUE	Empty	Eukaryotic initiatic	IF4A1_HUMAN	46 kDa	TRUE	0.27	[]	unknown	0	4	0	2
68.4	TRUE	Empty	Heat shock 70 kDa	HSP71_HUMAN	70 kDa	TRUE	0.63	[]	unknown	0	3	4	0
396.2	TRUE	Empty	40S ribosomal pro	RS27L_HUMAN	9 kDa	TRUE	0.43	[]	unknown	2	2	1	1
271.2	TRUE	Empty	Ezrin OS=Homo sa	EZRI_HUMAN	69 kDa	TRUE	0.56	[]	unknown	2	0	1	3
18.3	TRUE	Empty	RNA-binding moti	RMXL2_HUMAN	43 kDa	TRUE	0.0011	Control low, Patient high	unknown	0	0	17	0
570	TRUE	Empty	Hemoglobin subui	HBA_HUMAN	15 kDa		0.21	[]	unknown	0	2	0	0
571	TRUE	Empty	Survival motor ne	SMN_HUMAN	32 kDa		0.29	[]	unknown	0	0	0	2
572	TRUE	Empty	Transcription initi	TAF6_HUMAN	73 kDa		0.29	[]	unknown	0	0	0	2
573	TRUE	Empty	Filaggrin-2 OS=Ho	FILA2_HUMAN	248 kDa		0.21	[]	unknown	0	2	0	0
574	TRUE	Empty	Dynein light chain	DYL1_HUMAN (+1)	10 kDa		0.29	[]	unknown	0	0	0	2
139.2	TRUE	Empty	Spectrin beta chai	SPTN2_HUMAN	271 kDa	TRUE	0.54	[]	unknown	0	0	0	2
38.3	TRUE	Empty	Chromodomain-he	CHD5_HUMAN	223 kDa	TRUE	1	[]	unknown	0	0	16	0
8.4	TRUE	Empty	Histone H2A type	H2A2A_HUMAN	14 kDa	TRUE	0.19	[]	unknown	291	118	286	164
2.6	TRUE	Empty	Histone H2B type	H2B1L_HUMAN	14 kDa	TRUE	0.02	[]	unknown	184	84	225	136
127.2	TRUE	Empty	Lamina-associatec	LAP2A_HUMAN	75 kDa	TRUE	0.43	[]	unknown	12	4	8	8
168.2	TRUE	Empty	Histone deacetyla	HDAC1_HUMAN	55 kDa	TRUE	0.53	[]	unknown	4	4	6	8
71.3	TRUE	Empty	Polyadenylate-bin	PABP3_HUMAN	70 kDa	TRUE	0.22	[]	unknown	6	3	5	11
8.5	TRUE	Empty	Histone H2AX OS=	H2AX_HUMAN	15 kDa	TRUE	0.13	[]	unknown	208	90	200	128
16.2	TRUE	Empty	Heterogeneous nu	RA1L2_HUMAN	34 kDa	TRUE	0.18	[]	unknown	20	33	58	14
510.2	TRUE	Empty	SWI/SNF-related r	SMRD3_HUMAN	55 kDa	TRUE	0.71	[]	unknown	1	0	0	1
191.2	TRUE	Empty	RanBP2-like and G	RGPD4_HUMAN	197 kDa	TRUE	0.025	[]	unknown	0	0	7	0
113.3	TRUE	Empty	Eukaryotic initiatic	IF4A2_HUMAN	46 kDa	TRUE	0.27	[]	unknown	0	4	2	0
446.2	TRUE	Empty	Tubulin alpha-8 ch	TBA8_HUMAN	50 kDa	TRUE	0.54	[]	unknown	0	0	1	0
71.4	TRUE	Empty	Polyadenylate-bin	PAP1L_HUMAN	68 kDa	TRUE	0.045	[]	unknown	0	7	0	0
115.2	TRUE	Empty	Hepatoma-derivec	HDGR2_HUMAN	74 kDa	TRUE	1	[]	unknown	0	0	0	1
68.5	TRUE	Empty	Heat shock 70 kDa	HS71L_HUMAN	70 kDa	TRUE	0.46	[]	unknown	0	4	0	0
71.5	TRUE	Empty	Polyadenylate-bin	PABP5_HUMAN	43 kDa	TRUE	1	[]	unknown	0	0	0	2
207.3	TRUE	Empty	Alpha-actinin-2 OS	ACTN2_HUMAN	104 kDa	TRUE	1	[]	unknown	2	0	0	0
260.3	TRUE	Empty	Bromodomain tes	BRDT_HUMAN	108 kDa	TRUE	1	[]	unknown	0	0	0	0
2.7	TRUE	Empty	Putative histone H	H2B2C_HUMAN	21 kDa	TRUE	1	[]	unknown	0	0	0	0
24.2	TRUE	Empty	DNA topoisomera	TOP1M_HUMAN	70 kDa	TRUE	1	[]	unknown	0	0	0	6
95.3	TRUE	Empty	Probable ATP-dep	DDX4_HUMAN	79 kDa	TRUE	1	[]	unknown	0	0	1	0
445.2	TRUE	Empty	Putative 60S ribos	RL37L_HUMAN	11 kDa	TRUE	0.46	[]	unknown	0	1	0	0
4.2	TRUE	Empty	Epiplakin OS=Hom	EPIPL_HUMAN	556 kDa	TRUE	1	[]	unknown	0	0	2	0
191.3	TRUE	Empty	RANBP2-like and G	RGPD5_HUMAN (+1)	199 kDa	TRUE	1	[]	unknown	0	1	0	0
463.2	TRUE	Empty	Chromodomain-he	CHD7_HUMAN	336 kDa	TRUE	1	[]	unknown	0	0	0	0
358.2	TRUE	Empty	Small ubiquitin-rel	SUMO4_HUMAN	11 kDa	TRUE	1	[]	unknown	0	0	0	0
22.2	TRUE	Empty	Spermatid perinuc	STRBP_HUMAN	74 kDa	TRUE	1	[]	unknown	0	0	0	0
462.2	TRUE	Empty	Putative ATP-depe	DHX57_HUMAN	156 kDa	TRUE	1	[]	unknown	0	0	0	0
139.3	TRUE	Empty	Spectrin beta chai	SPTN4_HUMAN	289 kDa	TRUE	1	[]	unknown	0	0	0	0

END OF FILE

P14 aniPOND DP592

#	Visible?	Starred?	Identified I	Accession I	Alternate I	Molecular	Protein Gr	Fisher's Exa	Quantitativ	Quantitativ	Taxonomy	Total Spect control 0901C	Total Spect control BJ1C	Total Spect control BJ1D	Total Spect progeria 0031C	Total Spect progeria 1671J	Total Spectrum Count progeria 1671Q
1.1	TRUE	Empty	Vimentin C	VIME_HUMAN		54 kDa	TRUE	< 0.00010		control low	unknown	195	248	249	257	264	173
2.1	TRUE	Empty	Plectin OS=	PLEC_HUMAN		532 kDa	TRUE	< 0.00010		control low	unknown	55	188	101	152	329	306
3	TRUE	Empty	Histone H4	H4_HUMAN		11 kDa		0.036		[]	unknown	139	88	129	229	86	317
7	TRUE	Empty	Prelamin-A	LMNA_HUMAN		74 kDa	TRUE	0.017		[]	unknown	89	57	75	133	79	75
9	TRUE	Empty	Heterogen	HNRPU_HUMAN		91 kDa	TRUE	< 0.00010		control low	unknown	23	19	37	77	13	118
11	TRUE	Empty	Heterogen	HNRPC_HUMAN		34 kDa		0.027		[]	unknown	19	32	41	53	26	107
12	TRUE	Empty	Heterogen	ROA1_HUMAN		39 kDa	TRUE	0.42		[]	unknown	25	33	32	48	20	69
13	TRUE	Empty	Annexin A2	ANXA2_HUMAN		39 kDa		0.19		[]	unknown	36	26	35	43	38	91
14	TRUE	Empty	DNA-deper	PRKDC_HUMAN		469 kDa		0.0017		control low	unknown	8	24	24	15	14	111
15	TRUE	Empty	Heterogen	ROA2_HUMAN		37 kDa		0.27		[]	unknown	31	28	35	46	26	63
16	TRUE	Empty	Histone H1	H15_HUMAN		23 kDa	TRUE	0.00018		control low	unknown	56	34	49	66	23	53
18	TRUE	Empty	Heterogen	HNRPM_HUMAN		78 kDa		0.0014		control low	unknown	11	15	28	69	7	61
20	TRUE	Empty	Heterochro	HP1B3_HUMAN		61 kDa		0.51		[]	unknown	32	10	27	55	15	38
21	TRUE	Empty	60S ribosoı	RL4_HUMAN		48 kDa		0.13		[]	unknown	17	25	34	53	20	27
23	TRUE	Empty	Cytoskelet	CKAP4_HUMAN		66 kDa	TRUE	< 0.00010		control hig	unknown	23	33	48	45	11	2
24	TRUE	Empty	60S ribosoı	RL7A_HUMAN		30 kDa		0.027		[]	unknown	16	17	33	30	19	25
25	TRUE	Empty	Interleukin	ILF3_HUMAN		95 kDa	TRUE	0.017		[]	unknown	15	12	19	45	11	50
26	TRUE	Empty	Pre-mRNA-	PRP8_HUMAN		274 kDa		0.00064		control low	unknown	2	14	9	36	3	42
27	TRUE	Empty	Splicing fac	SFPQ_HUMAN		76 kDa	TRUE	0.046		[]	unknown	20	14	27	54	21	51
28	TRUE	Empty	Heterogen	ROA3_HUMAN		40 kDa	TRUE	0.3		[]	unknown	8	15	16	20	7	27
29	TRUE	Empty	Non-POU c	NONO_HUMAN		54 kDa	TRUE	0.018		[]	unknown	14	10	21	49	13	42
30	TRUE	Empty	ATP-depen	DHX9_HUMAN		141 kDa		0.00061		control low	unknown	13	3	21	20	7	80
32	TRUE	Empty	40S ribosoı	RS3_HUMAN		27 kDa		0.00034		control hig	unknown	20	18	33	29	16	16
33	TRUE	Empty	U5 small nı	U520_HUMAN		245 kDa		0.0012		control low	unknown	4	13	14	36	15	40
35	TRUE	Empty	60S ribosoı	RL13_HUMAN		24 kDa		0.0061		control hig	unknown	20	19	30	27	21	22
36	TRUE	Empty	Serine/argi	SRRM2_HUMAN		300 kDa		0.0035		control low	unknown	8	12	15	29	13	52
37	TRUE	Empty	Probable A	DDX5_HUMAN		69 kDa	TRUE	< 0.00010		control low	unknown	9	19	22	54	8	83
38	TRUE	Empty	40S ribosoı	RS3A_HUMAN		30 kDa		0.026		[]	unknown	14	8	26	22	12	16
40	TRUE	Empty	60S ribosoı	RL7_HUMAN		29 kDa		0.00073		control hig	unknown	11	15	22	15	10	12
42	TRUE	Empty	60S ribosoı	RL8_HUMAN		28 kDa		0.0027		control hig	unknown	10	17	21	18	11	12
41	TRUE	Empty	Heterogen	HNRPK_HUMAN		51 kDa		0.0004		control low	unknown	6	8	11	23	5	55
43	TRUE	Empty	Matrin-3 O	MATR3_HUMAN		95 kDa		0.0047		control low	unknown	5	9	11	32	6	34
45	TRUE	Empty	40S ribosoı	RS9_HUMAN		23 kDa		0.2		[]	unknown	8	11	13	14	11	15
44	TRUE	Empty	Nucleolin (	NUCL_HUMAN		77 kDa		< 0.00010		control low	unknown	1	1	7	15	5	67
46	TRUE	Empty	Desmoplak	DESP_HUMAN		332 kDa		0.49		[]	unknown	8	8	16	27	21	1
47	TRUE	Empty	Nuclear mi	NUMA1_HUMAN		238 kDa		< 0.00010		control low	unknown	1	6	5	32	3	50
49	TRUE	Empty	Hornerin C	HORN_HUMAN		282 kDa		0.0031		control hig	unknown	14	16	9	20	8	3
50	TRUE	Empty	60S ribosoı	RL5_HUMAN		34 kDa		0.53		[]	unknown	6	9	15	22	9	16
51	TRUE	Empty	Structural ı	SMC1A_HUMAN		143 kDa		< 0.00010		control low	unknown	0	7	6	33	0	39
53	TRUE	Empty	40S ribosoı	RS8_HUMAN		24 kDa		0.063		[]	unknown	10	11	19	17	10	17
54	TRUE	Empty	Caldesmor	CALD1_HUMAN		93 kDa		< 0.00010		control hig	unknown	6	6	36	16	10	3
55	TRUE	Empty	Core histor	H2AY_HUMAN		40 kDa	TRUE	0.46		[]	unknown	17	18	16	39	13	31
57	TRUE	Empty	Collagen al	CO6A3_HUMAN		344 kDa		0.5		[]	unknown	11	1	16	0	43	0
58	TRUE	Empty	40S ribosoı	RS16_HUMAN		16 kDa		0.088		[]	unknown	11	9	23	16	12	22
59	TRUE	Empty	Vigilin OS=	VIGLN_HUMAN		141 kDa		0.047		[]	unknown	5	9	21	30	5	1
60	TRUE	Empty	60S ribosoı	RL6_HUMAN		33 kDa		0.51		[]	unknown	6	10	10	18	9	13
52.1	TRUE	Empty	Ribosome-	RRBP1_HUMAN		152 kDa	TRUE	0.11		[]	unknown	11	12	24	28	22	7
61	TRUE	Empty	Heterogen	HNRPL_HUMAN		64 kDa	TRUE	0.094		[]	unknown	6	6	9	23	3	22
62	TRUE	Empty	40S ribosoı	RS14_HUMAN		16 kDa		0.0035		control hig	unknown	9	15	19	18	9	9
65	TRUE	Empty	Heterogen	HNRPR_HUMAN		71 kDa	TRUE	0.0028		control low	unknown	11	19	25	60	10	65
19.1	TRUE	Empty	SWI/SNF-re	SMCA5_HUMAN		122 kDa	TRUE	< 0.00010		control low	unknown	6	4	12	39	3	81
66	TRUE	Empty	DNA topois	TOP2B_HUMAN		183 kDa	TRUE	< 0.00010		control low	unknown	4	12	12	45	2	56
68	TRUE	Empty	Lamin-B1 C	LMNB1_HUMAN		66 kDa	TRUE	< 0.00010		control low	unknown	5	0	3	27	5	42
69	TRUE	Empty	Staphyloco	SND1_HUMAN		102 kDa		0.24		[]	unknown	5	8	18	25	5	10
70	TRUE	Empty	Splicing fac	SF3B3_HUMAN		136 kDa		0.00025		control low	unknown	2	5	9	21	1	42
71	TRUE	Empty	60S ribosoı	RL18_HUMAN		22 kDa		0.059		[]	unknown	8	12	21	18	8	19
73	TRUE	Empty	DNA topois	TOP2A_HUMAN		174 kDa	TRUE	< 0.00010		control low	unknown	0	7	18	64	1	36
72	TRUE	Empty	40S ribosoı	RS18_HUMAN		18 kDa		0.066		[]	unknown	10	9	14	12	10	13
74	TRUE	Empty	Pinin OS=H	PININ_HUMAN		82 kDa	TRUE	< 0.00010		control low	unknown	3	2	6	21	4	38
67.1	TRUE	Empty	60S ribosoı	RL3_HUMAN		46 kDa	TRUE	0.094		[]	unknown	12	10	17	25	11	9
63.1	TRUE	Empty	Filamin-A (	FLNA_HUMAN		281 kDa	TRUE	0.021		[]	unknown	2	26	16	17	15	12
75	TRUE	Empty	RNA-bindir	RALY_HUMAN		32 kDa		0.44		[]	unknown	8	10	13	23	5	18
76	TRUE	Empty	Interleukin	ILF2_HUMAN		43 kDa		0.14		[]	unknown	4	6	12	9	2	36
78	TRUE	Empty	Splicing fac	SF3B1_HUMAN		146 kDa		0.00012		control low	unknown	2	5	6	17	7	35
77	TRUE	Empty	Heterogen	HNRL2_HUMAN		85 kDa		< 0.00010		control low	unknown	5	0	7	19	2	41
39.1	TRUE	Empty	Myosin-9 (	MYH9_HUMAN		227 kDa	TRUE	0.15		[]	unknown	2	14	15	11	22	4
82	TRUE	Empty	40S ribosoı	RS2_HUMAN		31 kDa		0.027		[]	unknown	12	9	19	13	13	14
81	TRUE	Empty	Tyrosine-p	BAZ1B_HUMAN		171 kDa		< 0.00010		control low	unknown	0	4	4	23	1	28
83	TRUE	Empty	Nucleopho	NPM_HUMAN		33 kDa		0.3		[]	unknown	7	13	13	18	10	17
31.1	TRUE	Empty	Chromodo	CHD4_HUMAN		218 kDa	TRUE	< 0.00010		control low	unknown	2	8	10	30	1	49
84	TRUE	Empty	60S ribosoı	RL13A_HUMAN		24 kDa		0.017		[]	unknown	7	7	17	13	9	5
85	TRUE	Empty	Elongation	EF1A1_HUMAN (+1)		50 kDa		0.015		[]	unknown	11	10	21	10	12	18
86	TRUE	Empty	Serine/argi	SRSF1_HUMAN		28 kDa	TRUE	0.016		[]	unknown	2	8	14	16	12	36
87	TRUE	Empty	Heat shock	HSP7C_HUMAN		71 kDa	TRUE	0.00083		control low	unknown	2	4	15	11	10	50
88	TRUE	Empty	RNA-bindir	RBM14_HUMAN		69 kDa		0.001		control low	unknown	4	2	8	18	4	32
90	TRUE	Empty	60S ribosoı	RL24_HUMAN		18 kDa		0.03		[]	unknown	4	12	19	20	8	6
92	TRUE	Empty	40S ribosoı	RS11_HUMAN		18 kDa		0.011		control hig	unknown	6	6	15	13	3	5
91	TRUE	Empty	Poly [ADP-ı	PARP1_HUMAN		113 kDa		< 0.00010		control low	unknown	1	3	5	16	0	35
96	TRUE	Empty	40S ribosoı	RS6_HUMAN		29 kDa		0.015		[]	unknown	7	5	15	10	6	6
95	TRUE	Empty	Nucleolar I	DDX21_HUMAN		87 kDa	TRUE	< 0.00010		control low	unknown	1	1	4	30	2	30
17.1	TRUE	Empty	RNA-bindir	RBMX_HUMAN		42 kDa	TRUE	0.45		[]	unknown	31	17	17	46	10	50
97	TRUE	Empty	60S ribosoı	RL11_HUMAN		20 kDa		0.033		[]	unknown	4	10	15	17	5	5
99	TRUE	Empty	60S ribosoı	RL15_HUMAN		24 kDa		0.2		[]	unknown	5	5	9	8	9	5
100	TRUE	Empty	Heterogen	ROAA_HUMAN		36 kDa	TRUE	0.019		[]	unknown	5	7	12	19	7	35
98	TRUE	Empty	KH domain	KHDR1_HUMAN		48 kDa		0.26		[]	unknown	5	4	4	10	2	15
102	TRUE	Empty	60S ribosoı	RL29_HUMAN		18 kDa		0.072		[]	unknown	4	6	11	9	6	5
101	TRUE	Empty	ELAV-like p	ELAV1_HUMAN		36 kDa		0.0028		control low	unknown	4	4	5	15	5	28
79.1	TRUE	Empty	Polypyrimi	PTBP1_HUMAN		57 kDa	TRUE	0.15		[]	unknown	10	10	13	23	8	35
103	TRUE	Empty	116 kDa U5	U5S1_HUMAN		109 kDa	TRUE	0.0039		control low	unknown	1	6	7	21	5	23
104	TRUE	Empty	Splicing fac	SF3B2_HUMAN		100 kDa		0.0059		control low	unknown	1	2	4	10	3	18
105	TRUE	Empty	60S ribosoı	RL10A_HUMAN		25 kDa		0.011		control hig	unknown	12	7	14	10	7	11

106	TRUE	Empty	60S riboso	RL23A_HUMAN	18 kDa		0.078	[]	unknown	10	3	14	12	7	9
107	TRUE	Empty	40S riboso	RS13_HUMAN	17 kDa		0.18	[]	unknown	4	3	8	6	3	7
48.1	TRUE	Empty	40S riboso	RS4X_HUMAN	30 kDa	TRUE	0.12	[]	unknown	6	6	17	14	7	12
108	TRUE	Empty	Tumor sup	TP53B_HUMAN	214 kDa		< 0.00010	control low	unknown	0	4	0	10	0	41
110	TRUE	Empty	60S riboso	RL10_HUMAN	25 kDa		0.54	[]	unknown	2	1	8	10	2	6
109	TRUE	Empty	Protein DE	DEK_HUMAN	43 kDa		0.0074	control low	unknown	2	4	6	5	1	36
111	TRUE	Empty	Thyroid ho	TR150_HUMAN	109 kDa		0.0007	control low	unknown	2	2	5	12	3	28
64.1	TRUE	Empty	Transcripti	SMCA4_HUMAN	185 kDa	TRUE	< 0.00010	control low	unknown	0	3	0	17	0	53
22.1	TRUE	Empty	Histone H2	H2AJ_HUMAN	14 kDa	TRUE	0.21	[]	unknown	78	63	88	136	43	157
112	TRUE	Empty	Double-str	DSRAD_HUMAN	136 kDa		0.00016	control low	unknown	0	1	3	16	0	17
80.1	TRUE	Empty	DNA topois	TOP1_HUMAN	91 kDa	TRUE	0.06	[]	unknown	6	2	9	1	5	37
113	TRUE	Empty	60S riboso	RL14_HUMAN	23 kDa		0.21	[]	unknown	7	4	10	11	6	8
114	TRUE	Empty	Collagen al	CO1A1_HUMAN	139 kDa	TRUE	< 0.00010	control hig	unknown	8	9	31	3	2	0
115	TRUE	Empty	Structural	SMC3_HUMAN	142 kDa		< 0.00010	control low	unknown	0	0	4	18	1	26
117	TRUE	Empty	Serine/argi	SRSF7_HUMAN	27 kDa	TRUE	0.5	[]	unknown	4	9	8	12	4	16
116	TRUE	Empty	RuvB-like 2	RUVB2_HUMAN	51 kDa		0.00027	control low	unknown	2	4	3	19	2	25
119	TRUE	Empty	Heterogen	HNRDL_HUMAN	46 kDa	TRUE	0.00027	control low	unknown	8	7	8	21	5	54
118	TRUE	Empty	Guanine nu	GBLP_HUMAN	35 kDa		0.029	[]	unknown	14	2	5	12	2	3
120	TRUE	Empty	Lamin-B2 (	LMNB2_HUMAN	68 kDa	TRUE	0.013	control low	unknown	3	4	6	14	12	16
121	TRUE	Empty	High mobil	HMGA2_HUMAN	12 kDa		0.0024	control hig	unknown	11	12	13	11	10	6
122	TRUE	Empty	Heterogen	HNRH3_HUMAN	37 kDa		0.092	[]	unknown	5	4	4	12	6	15
123	TRUE	Empty	Ribosomal	RL1D1_HUMAN	55 kDa		0.0095	control low	unknown	0	4	2	17	1	9
124	TRUE	Empty	Serine/argi	SRSF3_HUMAN	19 kDa	TRUE	0.27	[]	unknown	6	8	8	12	3	13
126	TRUE	Empty	60S riboso	RL17_HUMAN	21 kDa		0.19	[]	unknown	2	4	11	11	3	5
125	TRUE	Empty	Pre-mRNA-	PRP6_HUMAN	107 kDa		0.12	[]	unknown	1	2	6	8	2	14
127	TRUE	Empty	60S riboso	RL27_HUMAN	16 kDa		0.0056	control hig	unknown	8	6	8	7	5	2
128	TRUE	Empty	Putative pr	DHX15_HUMAN	91 kDa	TRUE	< 0.00010	control low	unknown	0	1	2	16	1	22
129	TRUE	Empty	Activity-de	ADNP_HUMAN	124 kDa		< 0.00010	control low	unknown	0	0	3	10	0	27
8.1	TRUE	Empty	Histone H1	H12_HUMAN	21 kDa	TRUE	0.00042	control low	unknown	70	43	60	82	34	75
132	TRUE	Empty	60S riboso	RL12_HUMAN	18 kDa		0.19	[]	unknown	6	9	10	12	5	13
131	TRUE	Empty	Probable A	DDX17_HUMAN	80 kDa	TRUE	0.0004	control low	unknown	6	10	14	29	9	56
130	TRUE	Empty	Bcl-2-assoc	BCLF1_HUMAN	106 kDa		0.18	[]	unknown	3	1	4	9	2	9
133	TRUE	Empty	40S riboso	RS15A_HUMAN	15 kDa		0.0054	control hig	unknown	8	6	10	9	4	3
134	TRUE	Empty	FACT comp	SP16H_HUMAN	120 kDa		< 0.00010	control low	unknown	0	1	2	2	1	34
135	TRUE	Empty	Eukaryotic	IF4A3_HUMAN	47 kDa	TRUE	0.05	[]	unknown	4	3	5	17	1	16
136	TRUE	Empty	X-ray repai	XRCC5_HUMAN	83 kDa		< 0.00010	control low	unknown	0	0	1	6	0	25
139	TRUE	Empty	Serine/argi	SRSF6_HUMAN	40 kDa	TRUE	0.014	[]	unknown	4	5	7	21	9	18
138	TRUE	Empty	RuvB-like 1	RUVB1_HUMAN	50 kDa		0.0076	control low	unknown	2	3	4	15	3	17
137	TRUE	Empty	Proliferatir	PCNA_HUMAN	29 kDa		0.37	[]	unknown	1	2	8	8	0	6
141	TRUE	Empty	Small nucle	RSMB_HUMAN	25 kDa		0.025	[]	unknown	1	1	2	6	0	13
140	TRUE	Empty	40S riboso	RS5_HUMAN	23 kDa		0.046	[]	unknown	3	8	8	9	4	3
142	TRUE	Empty	60S riboso	RL23_HUMAN	15 kDa		0.16	[]	unknown	5	6	6	13	3	2
143	TRUE	Empty	40S riboso	RS20_HUMAN	13 kDa		0.25	[]	unknown	2	7	7	10	3	6
144	TRUE	Empty	Heterogen	HNRPD_HUMAN	38 kDa	TRUE	0.05	[]	unknown	6	11	12	20	7	40
145	TRUE	Empty	60S riboso	RL27A_HUMAN	17 kDa		0.015	[]	unknown	4	7	8	5	5	3
146	TRUE	Empty	Coronin-1C	COR1C_HUMAN	53 kDa	TRUE	0.029	[]	unknown	2	2	5	18	8	4
147	TRUE	Empty	Protein pol	PB1_HUMAN	193 kDa		0.00063	control low	unknown	0	1	1	6	0	17
150	TRUE	Empty	40S riboso	RS25_HUMAN	14 kDa		0.15	[]	unknown	5	3	6	3	5	6
148	TRUE	Empty	Apoptotic	ACINU_HUMAN	152 kDa		0.0022	control low	unknown	0	1	3	9	1	16
149	TRUE	Empty	DNA-direct	RPB1_HUMAN	217 kDa		0.0048	control low	unknown	0	2	1	9	1	11
151	TRUE	Empty	X-ray repai	XRCC6_HUMAN	70 kDa		< 0.00010	control low	unknown	0	0	1	5	0	24
153	TRUE	Empty	Heterogen	HNRPQ_HUMAN	70 kDa	TRUE	0.27	[]	unknown	11	15	25	39	12	40
154	TRUE	Empty	RNA-bindir	RBM25_HUMAN	100 kDa		< 0.00010	control low	unknown	0	0	2	13	3	19
156	TRUE	Empty	60S riboso	RL28_HUMAN	16 kDa		0.02	[]	unknown	6	8	7	10	2	4
155	TRUE	Empty	DNA replic	MCM3_HUMAN	91 kDa		< 0.00010	control low	unknown	0	0	1	7	0	25
8.2	TRUE	Empty	Histone H1	H14_HUMAN	22 kDa	TRUE	0.00045	control low	unknown	69	45	61	85	31	78
22.2	TRUE	Empty	Histone H2	H2A1C_HUMAN	14 kDa	TRUE	0.29	[]	unknown	74	62	85	134	43	152
158	TRUE	Empty	Junction pl	PLAK_HUMAN	82 kDa		0.21	[]	unknown	1	4	5	14	8	1
157	TRUE	Empty	Antigen KI-	KI67_HUMAN	359 kDa		< 0.00010	control low	unknown	0	0	0	18	0	4
164	TRUE	Empty	60S riboso	RL19_HUMAN	23 kDa		0.0032	control hig	unknown	5	4	9	4	2	3
165	TRUE	Empty	60S riboso	RL22_HUMAN	15 kDa		0.18	[]	unknown	6	8	7	7	7	10
163	TRUE	Empty	60S riboso	RL18A_HUMAN	21 kDa		0.26	[]	unknown	4	6	8	15	4	3
161	TRUE	Empty	ATP-depen	DDX3X_HUMAN	73 kDa	TRUE	< 0.00010	control low	unknown	0	1	2	9	0	31
162	TRUE	Empty	Metastasis	MTA2_HUMAN	75 kDa	TRUE	< 0.00010	control low	unknown	0	1	5	12	0	29
166	TRUE	Empty	40S riboso	RS17L_HUMAN (+1)	16 kDa		0.0034	control hig	unknown	5	6	10	5	6	1
167	TRUE	Empty	Probable A	DDX23_HUMAN	96 kDa		0.0029	control low	unknown	3	0	2	12	2	14
168	TRUE	Empty	Basement	PGBM_HUMAN	469 kDa		0.036	[]	unknown	1	13	3	8	5	0
171	TRUE	Empty	Protein-glu	TGM2_HUMAN	77 kDa	TRUE	0.57	[]	unknown	1	4	7	17	2	0
169	TRUE	Empty	Pre-mRNA-	PRP19_HUMAN	55 kDa		0.49	[]	unknown	1	4	6	10	0	6
172	TRUE	Empty	Beta-caten	CTBL1_HUMAN	65 kDa		0.032	[]	unknown	0	0	0	1	0	6
170	TRUE	Empty	40S riboso	RS24_HUMAN	15 kDa		0.18	[]	unknown	4	6	5	9	3	4
173	TRUE	Empty	Nestin OS=	NEST_HUMAN	177 kDa		0.39	[]	unknown	0	13	0	0	0	24
174	TRUE	Empty	PC4 and SF	PSIP1_HUMAN	60 kDa	TRUE	0.00074	control low	unknown	1	1	2	5	0	24
182	TRUE	Empty	40S riboso	RS23_HUMAN	16 kDa		0.45	[]	unknown	6	1	7	7	3	10
180	TRUE	Empty	40S riboso	RSSA_HUMAN	33 kDa		0.39	[]	unknown	3	3	7	8	7	9
175	TRUE	Empty	Cell divisio	CDC5L_HUMAN	92 kDa		0.075	[]	unknown	2	2	5	12	0	14
181	TRUE	Empty	SWI/SNF c	SMRC2_HUMAN	133 kDa	TRUE	< 0.00010	control low	unknown	1	1	1	10	1	28
178	TRUE	Empty	60S riboso	RL21_HUMAN	19 kDa		0.15	[]	unknown	3	4	7	7	4	3
179	TRUE	Empty	60S riboso	RL35A_HUMAN	13 kDa		0.31	[]	unknown	3	4	4	5	4	4
176	TRUE	Empty	High mobil	HMGA1_HUMAN	12 kDa		0.55	[]	unknown	3	6	6	11	3	10
177	TRUE	Empty	DNA replic	MCM5_HUMAN	82 kDa		< 0.00010	control low	unknown	0	0	0	6	0	23
56.1	TRUE	Empty	Polyadenyl	PABP1_HUMAN	71 kDa	TRUE	0.27	[]	unknown	3	6	17	19	7	23
183	TRUE	Empty	Heterogen	HNRH1_HUMAN	49 kDa	TRUE	0.093	[]	unknown	1	1	5	8	1	12
185	TRUE	Empty	40S riboso	RS19_HUMAN	16 kDa		0.13	[]	unknown	6	4	6	6	6	4
186	TRUE	Empty	Transcripti	TFAM_HUMAN	29 kDa		0.18	[]	unknown	2	1	5	0	3	17
184	TRUE	Empty	Sister chro	PDS5B_HUMAN	165 kDa	TRUE	0.0043	control low	unknown	0	1	0	7	0	8
187	TRUE	Empty	DNA replic	MCM6_HUMAN	93 kDa		0.00012	control low	unknown	0	0	1	6	0	17
192	TRUE	Empty	60S riboso	RL36L_HUMAN	12 kDa		0.43	[]	unknown	3	1	3	5	1	3
190	TRUE	Empty	Transform	TRA2B_HUMAN	34 kDa		0.048	[]	unknown	2	0	0	2	0	10
188	TRUE	Empty	DNA replic	MCM2_HUMAN	102 kDa		< 0.00010	control low	unknown	0	0	1	6	0	29
189	TRUE	Empty	Structural	SMHD1_HUMAN	226 kDa		0.00019	control low	unknown	0	1	0	11	0	11
191	TRUE	Empty	Zinc finger	ZFR_HUMAN	117 kDa		< 0.00010	control low	unknown	0	0	0	6	0	20
196	TRUE	Empty	Lysozyme (	LYSC_HUMAN	17 kDa		0.37	[]	unknown	4	2	5	3	6	5
195	TRUE	Empty	60S riboso	RL31_HUMAN	14 kDa		0.027	[]	unknown	3	4	7	4	1	4

193	TRUE	Empty	Myb-bindi	MBB1A_HUMAN	149 kDa		0.073	[]	unknown	3	3	1	13	1	8
194	TRUE	Empty	DNA replic	MCM7_HUMAN	81 kDa		0.00012	control low	unknown	1	0	1	5	0	22
199	TRUE	Empty	Spectrin be	SPTB2_HUMAN	275 kDa		0.067	[]	unknown	2	0	3	1	5	12
198	TRUE	Empty	Collagen al	CO6A1_HUMAN	109 kDa		0.23	[]	unknown	4	0	8	0	13	0
197	TRUE	Empty	ATPase fan	ATAD2_HUMAN	159 kDa	TRUE	< 0.00010	control low	unknown	0	0	0	7	0	13
200	TRUE	Empty	Transcripti	TIF1B_HUMAN	89 kDa		< 0.00010	control low	unknown	0	0	0	7	0	27
201	TRUE	Empty	Insulin-like	IF2B3_HUMAN	64 kDa	TRUE	0.23	[]	unknown	7	6	7	12	4	8
203	TRUE	Empty	60S ribosoı	RL36_HUMAN	12 kDa		0.15	[]	unknown	3	3	8	9	4	1
205	TRUE	Empty	Serine/argi	SRS10_HUMAN	31 kDa		0.12	[]	unknown	1	0	4	4	1	11
202	TRUE	Empty	RNA-bindir	RBM39_HUMAN	59 kDa		0.00048	control low	unknown	1	1	1	6	1	20
204	TRUE	Empty	40S ribosoı	RS26_HUMAN	13 kDa		0.4	[]	unknown	4	2	4	7	2	4
206	TRUE	Empty	Nuclease-s	YBOX1_HUMAN	36 kDa	TRUE	0.4	[]	unknown	2	5	8	6	2	19
209	TRUE	Empty	60S ribosoı	RL9_HUMAN	22 kDa		0.29	[]	unknown	4	4	6	6	7	4
208	TRUE	Empty	Chromatin	CHTOP_HUMAN	26 kDa		0.18	[]	unknown	3	7	5	7	4	5
210	TRUE	Empty	Serine/argi	SRSF9_HUMAN	26 kDa	TRUE	0.26	[]	unknown	6	0	2	9	3	6
213	TRUE	Empty	Filaggrin-2	FILA2_HUMAN	248 kDa		0.11	[]	unknown	7	6	2	8	4	2
214	TRUE	Empty	Regulator (	RCC1_HUMAN	45 kDa		0.071	[]	unknown	2	1	3	6	2	12
216	TRUE	Empty	U1 small nı	RU17_HUMAN	52 kDa		0.041	[]	unknown	0	1	2	2	1	12
217	TRUE	Empty	Splicing fac	SF3A3_HUMAN	59 kDa		0.021	[]	unknown	1	1	1	7	0	10
218	TRUE	Empty	U4/U6.U5	SNUT1_HUMAN	90 kDa		0.0016	control low	unknown	1	0	3	10	1	16
212	TRUE	Empty	Bromodom	BAZ1A_HUMAN	179 kDa		0.00063	control low	unknown	0	0	0	2	0	13
215	TRUE	Empty	60S ribosoı	RL30_HUMAN	13 kDa		0.033	[]	unknown	6	5	8	4	4	7
220	TRUE	Empty	Plasminog	PAIRB_HUMAN	45 kDa		0.44	[]	unknown	1	2	2	6	1	3
222	TRUE	Empty	Small nucle	SMD3_HUMAN	14 kDa		0.5	[]	unknown	3	4	2	4	1	8
221	TRUE	Empty	60S ribosoı	RL35_HUMAN	15 kDa		0.08	[]	unknown	2	3	4	3	0	3
225	TRUE	Empty	Desmoglei	DSG1_HUMAN	114 kDa	TRUE	0.57	[]	unknown	3	5	1	7	6	1
227	TRUE	Empty	Zinc finger	ZCH18_HUMAN	106 kDa		0.11	[]	unknown	1	1	1	5	1	6
223	TRUE	Empty	Neuroblast	AHNK_HUMAN	629 kDa		0.016	[]	unknown	0	0	2	0	4	10
224	TRUE	Empty	Probable A	DDX46_HUMAN	117 kDa		0.0014	control low	unknown	0	0	2	5	0	16
226	TRUE	Empty	THO compl	THOC2_HUMAN	183 kDa		0.01	control low	unknown	0	1	0	5	0	8
229	TRUE	Empty	Collagen al	CO1A2_HUMAN	129 kDa		< 0.00010	control hig	unknown	1	6	9	0	1	0
230	TRUE	Empty	Histone-lys	EHMT1_HUMAN	141 kDa		0.036	[]	unknown	0	0	1	6	0	4
232	TRUE	Empty	Heterogen	ROA0_HUMAN	31 kDa		0.12	[]	unknown	3	1	2	5	2	11
234	TRUE	Empty	FACT comp	SSRP1_HUMAN	81 kDa		0.0018	control low	unknown	0	0	1	1	0	16
231	TRUE	Empty	Pogo trans	POGZ_HUMAN	155 kDa		0.0032	control low	unknown	0	2	0	6	0	13
233	TRUE	Empty	Serrate RN	SRRT_HUMAN	101 kDa		0.00014	control low	unknown	0	0	0	4	0	14
235	TRUE	Empty	Transforma	TRRAP_HUMAN	438 kDa		0.00023	control low	unknown	0	0	0	4	0	13
239	TRUE	Empty	U2 snRNP-	SR140_HUMAN	118 kDa		0.034	[]	unknown	0	2	2	6	1	11
240	TRUE	Empty	DBIRD com	ZN326_HUMAN	66 kDa		0.007	control low	unknown	0	0	3	7	0	13
238	TRUE	Empty	40S ribosoı	RS12_HUMAN	15 kDa		0.25	[]	unknown	3	3	5	6	4	2
236	TRUE	Empty	rRNA 2'-O-	FBRL_HUMAN	34 kDa		0.44	[]	unknown	2	1	2	7	2	1
211.1	TRUE	Empty	Host cell fa	HCFC1_HUMAN	209 kDa	TRUE	0.0011	control low	unknown	0	0	1	3	0	15
237	TRUE	Empty	Replicatior	RFC4_HUMAN	40 kDa		0.31	[]	unknown	0	1	7	8	0	9
34.1	TRUE	Empty	Thrombos	TSP1_HUMAN	129 kDa	TRUE	0.0055	control low	unknown	22	19	26	34	33	0
249	TRUE	Empty	Pre-mRNA	PR40A_HUMAN	109 kDa		0.016	[]	unknown	0	0	1	3	1	8
248	TRUE	Empty	Putative ril	NOP2_HUMAN	89 kDa		0.0011	control low	unknown	1	0	2	12	2	11
251	TRUE	Empty	Spectrin al	SPTN1_HUMAN	285 kDa		0.00023	control low	unknown	0	0	0	0	6	11
250	TRUE	Empty	Splicing fac	SF3A1_HUMAN	89 kDa		0.01	control low	unknown	0	0	3	4	1	14
243	TRUE	Empty	Centromer	CENPV_HUMAN	30 kDa		0.29	[]	unknown	2	2	5	5	2	3
242	TRUE	Empty	Actin-like p	ACL6A_HUMAN	47 kDa		0.0091	control low	unknown	0	1	3	8	0	14
244	TRUE	Empty	Collagen al	CO6A2_HUMAN	109 kDa		0.51	[]	unknown	3	0	4	0	10	0
245	TRUE	Empty	DNA (cytos	DNMT1_HUMAN	183 kDa		0.00014	control low	unknown	0	0	0	5	0	13
246	TRUE	Empty	78 kDa gluc	GRP78_HUMAN	72 kDa	TRUE	0.24	[]	unknown	0	1	9	8	2	14
252	TRUE	Empty	YLP motif-c	YLPM1_HUMAN	220 kDa		0.19	[]	unknown	0	1	2	5	0	5
247	TRUE	Empty	Nucleolar (	NOG1_HUMAN	74 kDa		0.00014	control low	unknown	0	0	0	10	0	8
254	TRUE	Empty	Lipocalin-1	LCN1_HUMAN	19 kDa		0.57	[]	unknown	2	1	6	2	11	1
263	TRUE	Empty	60S ribosoı	RL34_HUMAN	13 kDa		0.14	[]	unknown	3	2	2	3	2	0
19.2	TRUE	Empty	Probable g	SMCA1_HUMAN	123 kDa	TRUE	0.0065	control low	unknown	6	3	11	23	3	34
259	TRUE	Empty	40S ribosoı	RS7_HUMAN	22 kDa		0.46	[]	unknown	2	4	4	6	2	6
253	TRUE	Empty	Core histor	H2AW_HUMAN	40 kDa	TRUE	0.32	[]	unknown	5	6	7	16	5	14
255	TRUE	Empty	Metastasis	MTA1_HUMAN	81 kDa	TRUE	0.00018	control low	unknown	0	1	2	7	1	18
257	TRUE	Empty	60S ribosoı	RL32_HUMAN	16 kDa		0.5	[]	unknown	3	1	5	7	2	4
262	TRUE	Empty	Protein Wi	WIZ_HUMAN	179 kDa		0.021	[]	unknown	0	0	3	5	0	12
260	TRUE	Empty	SWI/SNF cı	SMRC1_HUMAN	123 kDa	TRUE	< 0.00010	control low	unknown	0	0	2	10	0	28
258	TRUE	Empty	DNA-direct	RPB2_HUMAN	134 kDa		0.0018	control low	unknown	0	1	0	4	0	13
256	TRUE	Empty	Double-str	RAD21_HUMAN	72 kDa		< 0.00010	control low	unknown	0	0	0	7	0	12
261	TRUE	Empty	Helicase SI	SRCAP_HUMAN	344 kDa	TRUE	0.0045	control low	unknown	0	0	0	2	0	9
265	TRUE	Empty	Serum albu	ALBU_HUMAN (+1)	69 kDa	TRUE	0.00026	control hig	unknown	8	7	15	4	12	2
269	TRUE	Empty	Suprabasin	SBSN_HUMAN	61 kDa		0.43	[]	unknown	2	2	3	5	4	0
268	TRUE	Empty	Small nucle	SMD2_HUMAN	14 kDa		0.46	[]	unknown	1	1	5	3	3	7
267	TRUE	Empty	Nucleolar p	NOP56_HUMAN	66 kDa		< 0.00010	control low	unknown	1	0	0	20	2	2
266	TRUE	Empty	Cleavage a	CPSF1_HUMAN	161 kDa		0.00063	control low	unknown	0	0	0	5	0	10
272	TRUE	Empty	Protein Re	RED_HUMAN	66 kDa		< 0.00010	control low	unknown	0	0	0	10	0	10
273	TRUE	Empty	Peptidyl-pı	PPIB_HUMAN	24 kDa		0.15	[]	unknown	1	2	3	2	1	1
271	TRUE	Empty	AT-rich inte	ARI1A_HUMAN	242 kDa	TRUE	0.00014	control low	unknown	0	0	0	2	0	16
276	TRUE	Empty	Transcripti	P66A_HUMAN	68 kDa	TRUE	0.025	[]	unknown	2	0	5	5	2	19
277	TRUE	Empty	E3 ubiquiti	UHRF1_HUMAN	90 kDa	TRUE	< 0.00010	control low	unknown	0	0	0	3	0	18
275	TRUE	Empty	Integrator	INT1_HUMAN	244 kDa		< 0.00010	control low	unknown	0	0	0	6	0	14
283	TRUE	Empty	Desmocolli	DSC1_HUMAN	100 kDa		0.51	[]	unknown	1	2	0	3	3	0
279	TRUE	Empty	Chromatin	CAF1B_HUMAN	61 kDa		0.046	[]	unknown	0	1	3	5	0	12
281	TRUE	Empty	NHP2-like	NH2L1_HUMAN	14 kDa		0.19	[]	unknown	3	3	2	2	3	2
280	TRUE	Empty	Death-indu	DIDO1_HUMAN	244 kDa		0.001	control low	unknown	0	0	0	5	0	9
282	TRUE	Empty	WD40 repe	SMU1_HUMAN	58 kDa		0.068	[]	unknown	0	1	1	5	0	6
286	TRUE	Empty	Scaffold at	SAFB2_HUMAN	107 kDa	TRUE	0.21	[]	unknown	1	2	6	7	0	14
284	TRUE	Empty	N-acetyltra	NAT10_HUMAN	116 kDa		0.056	[]	unknown	1	1	1	7	2	5
285	TRUE	Empty	RNA-bindir	RNPS1_HUMAN	34 kDa		0.39	[]	unknown	1	1	2	4	0	5
287	TRUE	Empty	Nucleoprot	TPR_HUMAN	267 kDa		0.00038	control low	unknown	0	0	0	1	0	15
6.1	TRUE	Empty	Histone H3	H31_HUMAN (+1)	15 kDa	TRUE	0.00019	control hig	unknown	26	18	27	26	6	27
291	TRUE	Empty	Arginase-1	ARGI1_HUMAN	35 kDa		0.14	[]	unknown	2	2	3	3	2	0
289	TRUE	Empty	40S ribosoı	RS29_HUMAN	7 kDa		0.15	[]	unknown	1	6	4	7	1	2
288	TRUE	Empty	H/ACA ribc	DKC1_HUMAN	58 kDa		0.024	[]	unknown	0	1	0	8	1	2
290	TRUE	Empty	General tra	TF3C1_HUMAN	239 kDa		0.00038	control low	unknown	0	0	0	2	0	14
8.3	TRUE	Empty	Histone H1	H13_HUMAN	22 kDa	TRUE	0.0016	control low	unknown	64	40	56	79	30	73
5.1	TRUE	Empty	Beta-actin-	ACTBL_HUMAN	42 kDa	TRUE	< 0.00010	control hig	unknown	22	33	34	34	22	21

300	TRUE	Empty	Histone H1	H1X_HUMAN	22 kDa		0.35	[]	unknown	2	3	2	4	2	2
298	TRUE	Empty	LIM domai	LMO7_HUMAN	193 kDa		0.25	[]	unknown	0	0	2	1	4	2
301	TRUE	Empty	Ribosome	BRX1_HUMAN	41 kDa		0.37	[]	unknown	0	0	0	0	0	2
299	TRUE	Empty	Microtubu	MAP4_HUMAN	121 kDa		0.007	control hig	unknown	1	0	6	1	0	0
297	TRUE	Empty	Eukaryotic	EIF3A_HUMAN	167 kDa		0.5	[]	unknown	0	0	1	1	0	2
296	TRUE	Empty	Zinc finger	ZC11A_HUMAN	89 kDa		0.00023	control low	unknown	0	0	0	6	0	11
295	TRUE	Empty	Replicatior	RFA1_HUMAN	68 kDa		0.097	[]	unknown	0	0	2	3	0	7
309	TRUE	Empty	Histone-bi	RBBP4_HUMAN	48 kDa	TRUE	0.034	[]	unknown	1	1	2	8	1	9
302	TRUE	Empty	Caprin-1 O	CAPR1_HUMAN	78 kDa		0.084	[]	unknown	2	0	2	4	3	8
303	TRUE	Empty	Mitochond	IMMT_HUMAN	84 kDa		0.44	[]	unknown	2	1	2	2	7	1
306	TRUE	Empty	tRNA-splici	RTCB_HUMAN	55 kDa		0.57	[]	unknown	1	0	3	2	1	4
310	TRUE	Empty	40S ribosoi	RS30_HUMAN	7 kDa		0.0072	control hig	unknown	4	4	3	1	2	1
304	TRUE	Empty	Leucine-ric	LRC59_HUMAN	35 kDa		0.08	[]	unknown	3	1	5	1	2	3
308	TRUE	Empty	Enhancer c	ERH_HUMAN	12 kDa		0.57	[]	unknown	1	2	1	3	1	3
307	TRUE	Empty	E3 ubiquiti	TRIPC_HUMAN	220 kDa		0.012	control low	unknown	0	0	0	2	0	7
305	TRUE	Empty	Nucleolar	NOP58_HUMAN	60 kDa		0.00014	control low	unknown	0	0	0	18	0	0
317	TRUE	Empty	Protein S1(	S10A8_HUMAN	11 kDa		0.076	[]	unknown	4	3	3	2	5	0
316	TRUE	Empty	Ras GTPas(	G3BP2_HUMAN	54 kDa	TRUE	0.25	[]	unknown	1	1	1	4	3	2
313	TRUE	Empty	Pescadillo	PESC_HUMAN	68 kDa		0.0028	control low	unknown	1	0	0	8	3	5
312	TRUE	Empty	Filamin-B (	FLNB_HUMAN	278 kDa	TRUE	0.19	[]	unknown	0	8	0	3	0	4
311	TRUE	Empty	ATP-depen	DDX1_HUMAN	82 kDa		0.36	[]	unknown	0	0	5	4	0	7
315	TRUE	Empty	Paired amp	SIN3A_HUMAN	145 kDa		0.016	[]	unknown	0	1	0	2	0	10
314	TRUE	Empty	Remodelin	RSF1_HUMAN	164 kDa		0.0045	control low	unknown	0	0	0	2	0	9
5.2	TRUE	Empty	POTE anky	POTEF_HUMAN	121 kDa	TRUE	0.00034	control hig	unknown	21	41	36	30	33	28
320	TRUE	Empty	40S ribosoi	RS10_HUMAN	19 kDa		0.16	[]	unknown	1	3	1	1	1	1
319	TRUE	Empty	Insulin-like	IF2B1_HUMAN	63 kDa	TRUE	0.21	[]	unknown	4	4	5	10	2	2
322	TRUE	Empty	Small nucle	SMD1_HUMAN	13 kDa		0.52	[]	unknown	1	2	2	2	1	6
321	TRUE	Empty	SWI/SNF-r	SMCE1_HUMAN	47 kDa		0.0017	control low	unknown	0	0	0	5	0	8
323	TRUE	Empty	Zinc finger	ZN638_HUMAN	221 kDa		0.08	[]	unknown	1	0	0	3	0	5
159.1	TRUE	Empty	Lamina-ass	LAP2B_HUMAN	51 kDa	TRUE	0.00049	control low	unknown	1	2	3	14	2	20
329	TRUE	Empty	THO compl	THOC4_HUMAN	27 kDa		0.56	[]	unknown	1	3	2	2	2	5
327	TRUE	Empty	Scaffold at	SAFB1_HUMAN	103 kDa	TRUE	0.014	[]	unknown	1	2	4	11	0	17
330	TRUE	Empty	SAFB-like t	SLTM_HUMAN	117 kDa		0.62	[]	unknown	3	0	0	3	0	2
328	TRUE	Empty	Transcripti	SPT6H_HUMAN	199 kDa		0.0073	control low	unknown	0	0	0	3	0	7
325	TRUE	Empty	General tra	GTF2I_HUMAN	112 kDa		0.024	[]	unknown	0	0	1	1	0	10
326	TRUE	Empty	Nipped-B-l	NIPBL_HUMAN	316 kDa		0.0045	control low	unknown	0	0	0	2	0	9
293.1	TRUE	Empty	Chromobo	CBX3_HUMAN	21 kDa	TRUE	0.19	[]	unknown	1	2	2	6	1	7
334	TRUE	Empty	U4/U6.U5	SNUT2_HUMAN	65 kDa		0.31	[]	unknown	1	2	1	5	0	5
336	TRUE	Empty	Transform	TRA2A_HUMAN	33 kDa		0.016	[]	unknown	1	0	0	2	0	10
207.1	TRUE	Empty	Moesin OS	MOES_HUMAN	68 kDa	TRUE	0.17	[]	unknown	1	1	7	1	4	3
335	TRUE	Empty	Transducin	TBL2_HUMAN	50 kDa		0.08	[]	unknown	3	0	6	3	2	1
333	TRUE	Empty	60S ribosoi	RL38_HUMAN	8 kDa		0.48	[]	unknown	2	3	1	7	0	1
332	TRUE	Empty	Probable A	DDX27_HUMAN	90 kDa		0.0027	control low	unknown	0	0	0	9	1	2
331	TRUE	Empty	AT-rich int	ARI1B_HUMAN	236 kDa	TRUE	0.052	[]	unknown	0	0	0	0	0	6
8.4	TRUE	Empty	Histone H1	H11_HUMAN	22 kDa	TRUE	0.1	[]	unknown	26	17	22	44	14	24
343	TRUE	Empty	Filaggrin O	FILA_HUMAN	435 kDa		0.016	[]	unknown	5	1	0	0	1	0
348	TRUE	Empty	Transcripti	P66B_HUMAN	65 kDa	TRUE	0.046	[]	unknown	1	0	3	2	1	15
352	TRUE	Empty	Targeting	TPX2_HUMAN	86 kDa		0.36	[]	unknown	0	0	1	3	0	1
350	TRUE	Empty	SURP and	SUGP2_HUMAN	120 kDa		0.64	[]	unknown	0	1	1	1	0	2
338	TRUE	Empty	Apoptosis i	API5_HUMAN	59 kDa		0.57	[]	unknown	0	1	1	2	0	2
351	TRUE	Empty	High mobil	HMGN4_HUMAN	10 kDa		0.15	[]	unknown	3	1	2	3	1	0
349	TRUE	Empty	Nucleolar t	UBF1_HUMAN	89 kDa		0.12	[]	unknown	0	0	1	4	0	3
344	TRUE	Empty	Ras GTPas(	G3BP1_HUMAN	52 kDa	TRUE	0.14	[]	unknown	0	2	0	3	1	5
341	TRUE	Empty	DNA dama	DDB1_HUMAN	127 kDa		0.02	[]	unknown	0	0	0	3	0	5
340	TRUE	Empty	Parafibrom	CDC73_HUMAN	61 kDa		0.032	[]	unknown	0	0	0	1	0	6
342	TRUE	Empty	Histone-lys	EHMT2_HUMAN	132 kDa		0.052	[]	unknown	0	0	0	3	0	3
345	TRUE	Empty	Gamma-int	IF16_HUMAN	88 kDa		0.012	control low	unknown	0	0	0	0	0	9
339	TRUE	Empty	Cell cycle a	CCAR2_HUMAN	103 kDa		0.0045	control low	unknown	0	0	0	3	1	7
346	TRUE	Empty	Lysine-spe	KDM1A_HUMAN	93 kDa		0.001	control low	unknown	0	0	0	4	0	10
347	TRUE	Empty	Midasin OS	MDN1_HUMAN	633 kDa		0.086	[]	unknown	0	0	0	0	0	5
294.1	TRUE	Empty	Fibrillin-1 (	FBN1_HUMAN	312 kDa	TRUE	< 0.00010	control hig	unknown	0	16	0	0	0	0
89.1	TRUE	Empty	Spliceosor	DX39B_HUMAN	49 kDa	TRUE	0.2	[]	unknown	7	5	9	12	6	25
360	TRUE	Empty	Guanine nu	GNL3_HUMAN	62 kDa		0.12	[]	unknown	1	0	0	4	0	3
357	TRUE	Empty	Protein SO	SON_HUMAN	264 kDa		0.054	[]	unknown	0	0	1	3	0	6
356	TRUE	Empty	SAP domai	SARNP_HUMAN	24 kDa		0.57	[]	unknown	2	1	1	1	2	4
362	TRUE	Empty	Unconvent	MYO1C_HUMAN	122 kDa	TRUE	0.41	[]	unknown	2	0	1	2	2	3
361	TRUE	Empty	RNA-bindir	RB12B_HUMAN	118 kDa		0.036	[]	unknown	1	0	0	3	0	7
358	TRUE	Empty	2,4-dienoy	DECR_HUMAN	36 kDa		0.24	[]	unknown	2	1	2	2	2	0
354	TRUE	Empty	DNA replic	MCM4_HUMAN	97 kDa		0.0073	control low	unknown	0	0	0	2	0	8
359	TRUE	Empty	Phenylalan	SYFB_HUMAN	66 kDa		0.57	[]	unknown	0	1	1	0	3	1
355	TRUE	Empty	Poly(U)-bir	PUF60_HUMAN	60 kDa		0.02	[]	unknown	0	0	0	0	0	8
369	TRUE	Empty	Dermcidin	DCD_HUMAN	11 kDa		0.35	[]	unknown	4	1	2	1	6	1
56.2	TRUE	Empty	Polyadenyl	PABP4_HUMAN	71 kDa	TRUE	0.57	[]	unknown	1	2	7	9	2	6
371	TRUE	Empty	Coronin-1E	COR1B_HUMAN	54 kDa		0.25	[]	unknown	0	1	1	2	1	4
241.1	TRUE	Empty	ATPase fan	ATD3A_HUMAN	71 kDa	TRUE	0.53	[]	unknown	2	4	4	3	3	9
364	TRUE	Empty	Proline-, gl	PELP1_HUMAN	120 kDa		0.01	control low	unknown	0	1	0	5	0	8
370	TRUE	Empty	40S ribosoi	RS15_HUMAN	17 kDa		0.047	[]	unknown	2	2	3	1	1	1
368	TRUE	Empty	UAP56-inte	UIF_HUMAN	36 kDa		0.62	[]	unknown	0	2	1	1	1	3
363	TRUE	Empty	Nuclear re	NCOA5_HUMAN	66 kDa		0.62	[]	unknown	1	1	1	5	0	0
367	TRUE	Empty	Superkiller	SK2L2_HUMAN	118 kDa		0.02	[]	unknown	0	0	0	5	0	3
365	TRUE	Empty	U4/U6 sma	PRP31_HUMAN	55 kDa		0.054	[]	unknown	0	0	1	4	0	5
366	TRUE	Empty	Replicatior	RFC5_HUMAN	38 kDa		0.32	[]	unknown	0	1	2	3	0	5
10.1	TRUE	Empty	Ubiquitin-4	RS27A_HUMAN	18 kDa	TRUE	0.0066	control low	unknown	36	40	48	66	28	49
152.1	TRUE	Empty	Histone de	HDAC2_HUMAN	55 kDa	TRUE	0.025	[]	unknown	1	2	4	11	0	15
378	TRUE	Empty	Polyadenyl	PABP2_HUMAN	33 kDa		0.52	[]	unknown	1	3	1	1	0	8
160.1	TRUE	Empty	ADP/ATP tı	ADT2_HUMAN	33 kDa	TRUE	0.57	[]	unknown	2	5	5	6	4	9
376	TRUE	Empty	Coiled-coil	CCD86_HUMAN	40 kDa		0.032	[]	unknown	0	0	0	1	1	5
373	TRUE	Empty	Polymeras	PTRF_HUMAN	43 kDa		0.59	[]	unknown	1	0	3	0	1	5
375	TRUE	Empty	Protein tra	S61A1_HUMAN	52 kDa		0.047	[]	unknown	3	1	3	0	2	1
377	TRUE	Empty	YTH domai	YTDC1_HUMAN	85 kDa		0.23	[]	unknown	0	0	0	2	0	1
374	TRUE	Empty	Putative Rl	RBM15_HUMAN	107 kDa	TRUE	0.052	[]	unknown	0	0	0	1	0	5
372	TRUE	Empty	Bromodon	BRD1_HUMAN	120 kDa		0.02	[]	unknown	0	0	0	3	0	5
292.1	TRUE	Empty	K22E_HUM	K22E_HUMAN-DECOY	?	TRUE	0.47	[]	unknown	1	1	2	3	3	2
393	TRUE	Empty	TREMBL:Q	Q3SX09	?		0.15	[]	unknown	2	4	0	4	0	0

394	TRUE	Empty	Eukaryotic	IF4A1_HUMAN	46 kDa	TRUE	0.19	[]	unknown	0	2	1	5	0	6
395	TRUE	Empty	U2 small n	RU2A_HUMAN	28 kDa		0.12	[]	unknown	0	0	1	1	0	6
392	TRUE	Empty	Elongation	EF1G_HUMAN	50 kDa		0.49	[]	unknown	1	1	2	1	2	2
384	TRUE	Empty	PH-interac	PHIP_HUMAN	207 kDa	TRUE	0.17	[]	unknown	0	1	0	3	0	3
386	TRUE	Empty	Replication	RFC2_HUMAN	39 kDa		0.51	[]	unknown	0	0	3	3	0	3
390	TRUE	Empty	AT-rich int	ARID2_HUMAN	197 kDa		0.012	control low	unknown	0	0	0	1	0	8
381	TRUE	Empty	Actin filam	AFAP1_HUMAN	81 kDa		0.23	[]	unknown	0	0	0	0	0	3
388	TRUE	Empty	SNW doma	SNW1_HUMAN	61 kDa		0.17	[]	unknown	0	1	0	3	0	3
385	TRUE	Empty	Paraspeckl	PSPC1_HUMAN	59 kDa		0.0027	control low	unknown	0	0	0	2	0	10
389	TRUE	Empty	Symplekin	SYMPK_HUMAN	141 kDa		0.14	[]	unknown	0	0	0	0	0	4
387	TRUE	Empty	Replication	RFC3_HUMAN	41 kDa		0.51	[]	unknown	0	0	3	2	0	4
391	TRUE	Empty	Protein FA	F208A_HUMAN	189 kDa		0.086	[]	unknown	0	0	0	3	0	2
382	TRUE	Empty	Nucleosom	BPTF_HUMAN	338 kDa		0.052	[]	unknown	0	0	0	0	0	6
383	TRUE	Empty	Collagen al	COCA1_HUMAN	333 kDa		0.0002	control hig	unknown	0	9	0	0	0	0
5.3	TRUE	Empty	Actin, cyto	ACTB_BOVIN (+1)	42 kDa	TRUE	< 0.00010	control hig	unknown	80	122	117	112	93	71
404	TRUE	Empty	U4/U6 sma	PRPF3_HUMAN	78 kDa		0.14	[]	unknown	1	0	1	2	2	5
402	TRUE	Empty	Serpin H1	SERPH_HUMAN	46 kDa		0.0047	control hig	unknown	2	1	6	1	1	0
406	TRUE	Empty	60S ribosor	RL37A_HUMAN	10 kDa		0.16	[]	unknown	2	2	1	2	1	0
403	TRUE	Empty	Zinc finger	ZC3HE_HUMAN	83 kDa		0.02	[]	unknown	0	0	0	3	0	5
318.1	TRUE	Empty	Copine-3 C	CPNE3_HUMAN	60 kDa	TRUE	0.036	[]	unknown	0	0	1	3	1	6
405	TRUE	Empty	Serpin B3	SPB3_HUMAN	45 kDa		0.14	[]	unknown	0	0	0	1	3	0
401	TRUE	Empty	Telomere-a	RIF1_HUMAN	274 kDa		0.052	[]	unknown	0	0	0	1	0	5
399	TRUE	Empty	ATP-depen	DDX50_HUMAN	83 kDa	TRUE	0.001	control low	unknown	0	0	0	8	0	6
398	TRUE	Empty	Cell divisio	CDA7L_HUMAN	52 kDa		0.02	[]	unknown	0	0	0	1	0	7
397	TRUE	Empty	Chromobo	CBX5_HUMAN	22 kDa		0.55	[]	unknown	0	2	1	3	0	1
400	TRUE	Empty	E1A-bindin	EP400_HUMAN	343 kDa		0.14	[]	unknown	0	0	0	1	0	3
396	TRUE	Empty	Bromodom	BAZ2A_HUMAN	211 kDa		0.02	[]	unknown	0	0	0	0	0	8
419	TRUE	Empty	Insulin-like	IF2B2_HUMAN	66 kDa	TRUE	0.12	[]	unknown	2	4	4	6	1	1
264.1	TRUE	Empty	Poly(rC)-bi	PCBP1_HUMAN	37 kDa	TRUE	0.0072	control low	unknown	0	0	2	1	2	14
416	TRUE	Empty	SWI/SNF-r	SNF5_HUMAN	44 kDa		0.052	[]	unknown	0	0	0	1	0	5
228.1	TRUE	Empty	Serine/thr	PP1A_HUMAN	38 kDa	TRUE	0.36	[]	unknown	2	0	3	2	2	7
417	TRUE	Empty	Gelsolin O	GELS_HUMAN	86 kDa		0.17	[]	unknown	0	0	1	1	3	2
418	TRUE	Empty	Proliferatic	PA2G4_HUMAN	44 kDa		0.64	[]	unknown	1	0	1	2	0	1
410	TRUE	Empty	Protein RR	RRP5_HUMAN	209 kDa		0.12	[]	unknown	0	1	0	5	0	2
415	TRUE	Empty	Serpin B12	SPB12_HUMAN	46 kDa		0.02	[]	unknown	0	0	0	4	4	0
353.1	TRUE	Empty	RNA-bindir	FUS_HUMAN	53 kDa	TRUE	0.012	control low	unknown	0	0	0	0	1	8
324.1	TRUE	Empty	Alpha-actir	ACTN1_HUMAN	103 kDa	TRUE	0.17	[]	unknown	0	0	1	3	3	0
412	TRUE	Empty	DNA ligase	DNLI3_HUMAN	113 kDa		0.052	[]	unknown	0	0	0	3	0	3
413	TRUE	Empty	WD repeat	WDR5_HUMAN	37 kDa		0.57	[]	unknown	1	0	1	0	0	4
414	TRUE	Empty	Zinc finger	ZMYM2_HUMAN	155 kDa		0.02	[]	unknown	0	0	0	0	0	8
408	TRUE	Empty	ATP-depen	DDX54_HUMAN	99 kDa		0.032	[]	unknown	0	0	0	4	1	2
411	TRUE	Empty	General tra	TF3C3_HUMAN	101 kDa		0.0073	control low	unknown	0	0	0	1	0	9
409	TRUE	Empty	Putative ox	GLYR1_HUMAN	61 kDa		0.0045	control low	unknown	0	0	0	6	0	5
94.1	TRUE	Empty	60S ribosor	RL26_HUMAN	17 kDa	TRUE	0.00076	control hig	unknown	11	7	11	10	6	1
440	TRUE	Empty	C-terminal	CTBP1_HUMAN	48 kDa	TRUE	0.19	[]	unknown	0	0	2	2	0	6
439	TRUE	Empty	Sister chro	PDS5A_HUMAN	151 kDa	TRUE	0.36	[]	unknown	0	0	1	1	0	3
442	TRUE	Empty	Protein EL	ELYS_HUMAN	253 kDa		0.64	[]	unknown	2	0	0	2	0	1
434	TRUE	Empty	SUN doma	SUN1_HUMAN	90 kDa		0.12	[]	unknown	0	1	0	4	0	3
437	TRUE	Empty	Mediator c	MDC1_HUMAN	227 kDa		0.032	[]	unknown	0	0	0	1	0	6
431	TRUE	Empty	Constitutiv	F120A_HUMAN	122 kDa		0.086	[]	unknown	0	0	0	1	0	4
424	TRUE	Empty	Eukaryotic	EIF3B_HUMAN	92 kDa		0.41	[]	unknown	0	0	3	4	0	3
422	TRUE	Empty	Bromodom	BRD2_HUMAN	88 kDa	TRUE	0.0045	control low	unknown	0	0	0	2	0	9
428	TRUE	Empty	General tra	TF3C5_HUMAN	60 kDa		0.0073	control low	unknown	0	0	0	1	0	9
430	TRUE	Empty	Probable r	EBP2_HUMAN	35 kDa		0.08	[]	unknown	0	0	1	3	2	3
436	TRUE	Empty	Cysteine a	CSRP2_HUMAN	21 kDa		0.085	[]	unknown	1	2	2	0	2	0
441	TRUE	Empty	Cohesin su	STAG2_HUMAN	141 kDa	TRUE	0.23	[]	unknown	0	0	0	1	0	2
433	TRUE	Empty	Transcripti	SPT5H_HUMAN	121 kDa		0.032	[]	unknown	0	0	0	1	0	6
435	TRUE	Empty	Zinc finger	ZMYM3_HUMAN	152 kDa	TRUE	0.0073	control low	unknown	0	0	0	2	0	8
380.1	TRUE	Empty	Tropomyo	TPM4_HUMAN	29 kDa	TRUE	0.55	[]	unknown	1	0	2	0	4	0
432	TRUE	Empty	Nucleolar	NOP16_HUMAN	21 kDa		0.23	[]	unknown	0	0	0	0	1	2
425	TRUE	Empty	Heterogen	HNRL1_HUMAN	96 kDa	TRUE	0.14	[]	unknown	0	0	0	3	0	1
426	TRUE	Empty	SWI/SNF-r	SMRD2_HUMAN	59 kDa	TRUE	0.36	[]	unknown	1	0	0	1	0	3
423	TRUE	Empty	Elongation	EF1D_HUMAN	31 kDa		0.3	[]	unknown	0	0	3	0	1	1
438	TRUE	Empty	Eukaryotic	EIF3L_HUMAN	67 kDa		0.36	[]	unknown	0	0	1	3	0	1
429	TRUE	Empty	THO compl	THOC1_HUMAN	76 kDa		0.012	control low	unknown	0	0	0	4	0	5
427	TRUE	Empty	Splicing fac	SPF45_HUMAN	45 kDa		0.012	control low	unknown	0	0	0	3	0	6
421	TRUE	Empty	Annexin A	ANXA6_HUMAN	76 kDa		0.052	[]	unknown	0	0	0	0	0	6
228.2	TRUE	Empty	Serine/thr	PP1B_HUMAN	37 kDa	TRUE	0.44	[]	unknown	2	1	2	2	2	6
451	TRUE	Empty	U4/U6 sma	PRP4_HUMAN	58 kDa		0.12	[]	unknown	0	0	1	4	0	3
457	TRUE	Empty	TAR DNA-b	TADBP_HUMAN	45 kDa		0.49	[]	unknown	1	0	3	1	0	4
453	TRUE	Empty	Zinc finger	REQU_HUMAN	44 kDa		0.08	[]	unknown	0	0	1	1	0	7
461	TRUE	Empty	Chromodo	CHD2_HUMAN	211 kDa	TRUE	0.25	[]	unknown	0	0	1	0	1	4
31.2	TRUE	Empty	Chromodo	CHD3_HUMAN	227 kDa	TRUE	0.0033	control low	unknown	0	1	2	9	0	14
447	TRUE	Empty	Eukaryotic	IF2A_HUMAN	36 kDa		0.51	[]	unknown	0	1	2	0	1	5
443	TRUE	Empty	Actin-relat	ARPC4_HUMAN	20 kDa		0.45	[]	unknown	0	0	2	1	1	3
450	TRUE	Empty	PHD finger	PHF14_HUMAN	100 kDa		0.02	[]	unknown	0	0	0	1	0	7
455	TRUE	Empty	SPATS2-lik	SPS2L_HUMAN	62 kDa		0.052	[]	unknown	0	0	0	5	0	1
460	TRUE	Empty	Bifunctiona	SYEP_HUMAN	171 kDa		0.66	[]	unknown	0	1	0	0	0	2
454	TRUE	Empty	SWI/SNF-r	SMRD1_HUMAN	58 kDa	TRUE	0.08	[]	unknown	0	0	1	0	0	8
459	TRUE	Empty	Cytoskelet	CKAP5_HUMAN	226 kDa		0.14	[]	unknown	0	0	0	3	0	1
449	TRUE	Empty	Prolyl 4-hy	P4HA1_HUMAN	61 kDa		0.57	[]	unknown	0	0	2	4	0	0
444	TRUE	Empty	Probable A	DDX6_HUMAN	54 kDa		0.14	[]	unknown	0	0	0	0	0	4
446	TRUE	Empty	Histone H1	H10_HUMAN	21 kDa		0.43	[]	unknown	3	0	0	3	0	0
456	TRUE	Empty	Cohesin su	STAG1_HUMAN	144 kDa	TRUE	0.052	[]	unknown	0	0	0	1	0	5
448	TRUE	Empty	Kinesin-lik	KIF22_HUMAN	73 kDa		0.032	[]	unknown	0	0	0	1	0	6
445	TRUE	Empty	Glypican-1	GPC1_HUMAN	62 kDa		0.032	[]	unknown	0	0	0	0	0	7
458	TRUE	Empty	General tra	TF3C4_HUMAN	92 kDa		0.37	[]	unknown	0	0	0	0	0	2
452	TRUE	Empty	Putative R	RBM3_HUMAN	17 kDa		0.032	[]	unknown	0	0	0	0	0	7
4.1	TRUE	Empty	Histone H2	H2B1O_HUMAN	14 kDa	TRUE	0.16	[]	unknown	53	34	54	74	24	101
270.1	TRUE	Empty	Hemoglobi	HBA_HUMAN	15 kDa	TRUE	0.048	[]	unknown	1	8	0	5	0	0
278.1	TRUE	Empty	Bromodom	BRD4_HUMAN	152 kDa	TRUE	0.0048	control low	unknown	1	0	1	7	0	11
478	TRUE	Empty	Procollage	PLOD1_HUMAN	84 kDa		0.34	[]	unknown	0	1	1	2	4	0
473	TRUE	Empty	Serine/argi	SRSF2_HUMAN	25 kDa		0.08	[]	unknown	0	0	1	1	1	6
470	TRUE	Empty	Solute carr	GTR1_HUMAN	54 kDa		0.032	[]	unknown	0	0	0	0	0	7

476	TRUE	Empty	Zinc finger	ZMYM4_HUMAN	173 kDa	TRUE	0.032	[]	unknown	0	0	0	1	0	6
472	TRUE	Empty	Histone-bi	RBBP7_HUMAN	48 kDa	TRUE	0.34	[]	unknown	1	0	1	5	0	1
466	TRUE	Empty	Inner centr	INCE_HUMAN	105 kDa		0.25	[]	unknown	0	0	1	4	0	1
469	TRUE	Empty	Eukaryotic	EIF3E_HUMAN	52 kDa		0.43	[]	unknown	0	0	3	2	1	0
479	TRUE	Empty	HEAT repe	HEAT1_HUMAN	242 kDa		0.14	[]	unknown	0	0	0	2	0	2
474	TRUE	Empty	Zinc finger	ZC3H4_HUMAN	140 kDa		0.23	[]	unknown	0	0	0	0	0	3
464	TRUE	Empty	Eukaryotic	EIF3C_HUMAN (+1)	105 kDa		0.17	[]	unknown	0	0	1	2	0	4
477	TRUE	Empty	Double-str	MRE11_HUMAN	81 kDa		0.23	[]	unknown	0	0	0	1	0	2
475	TRUE	Empty	Polycomb	SUZ12_HUMAN	83 kDa		0.23	[]	unknown	0	0	0	0	0	3
468	TRUE	Empty	Pleiotropic	PLRG1_HUMAN	57 kDa		0.14	[]	unknown	0	0	0	3	0	1
467	TRUE	Empty	Nucleolar	NOC2L_HUMAN	85 kDa		0.086	[]	unknown	0	0	0	4	0	1
471	TRUE	Empty	MAX gene-	MGAP_HUMAN	332 kDa		0.086	[]	unknown	0	0	0	0	0	5
465	TRUE	Empty	Importin su	IMA1_HUMAN	58 kDa		0.086	[]	unknown	0	0	0	0	0	5
463	TRUE	Empty	Chromodo	CHD1_HUMAN	197 kDa	TRUE	0.012	control low	unknown	0	0	0	0	0	9
219.1	TRUE	Empty	Histone H2	H2AZ_HUMAN	14 kDa	TRUE	0.078	[]	unknown	65	53	80	111	38	115
264.2	TRUE	Empty	Poly(rC)-bi	PCBP2_HUMAN	39 kDa	TRUE	0.097	[]	unknown	0	0	2	1	2	7
501	TRUE	Empty	Nucleolar	NOC3L_HUMAN	93 kDa		0.57	[]	unknown	1	0	1	1	2	1
495	TRUE	Empty	Peptidyl-pr	PPIG_HUMAN	89 kDa		0.086	[]	unknown	0	0	0	0	1	4
485	TRUE	Empty	N-acylneur	NEUA_HUMAN	48 kDa		0.25	[]	unknown	1	0	0	2	0	3
499	TRUE	Empty	Galectin-1	LEG1_HUMAN	15 kDa		0.16	[]	unknown	1	2	1	0	2	0
483	TRUE	Empty	Glutamate	DHE3_HUMAN (+1)	61 kDa		0.27	[]	unknown	1	1	2	3	0	0
500	TRUE	Empty	Metastasis	MTA3_HUMAN	68 kDa	TRUE	0.0073	control low	unknown	0	0	0	3	0	8
503	TRUE	Empty	Spliceosom	CWC15_HUMAN	27 kDa		0.5	[]	unknown	0	1	1	0	0	2
1.2	TRUE	Empty	Alpha-inter	AINX_HUMAN	55 kDa	TRUE	0.47	[]	unknown	0	0	19	15	14	0
494	TRUE	Empty	Replicatior	RFC1_HUMAN	128 kDa		0.14	[]	unknown	0	0	0	2	0	2
492	TRUE	Empty	Double-str	STAU1_HUMAN	63 kDa		0.36	[]	unknown	1	0	0	2	0	2
504	TRUE	Empty	Nucleolar a	NOLC1_HUMAN	74 kDa		0.059	[]	unknown	3	0	0	0	0	0
498	TRUE	Empty	Integrator	INT6_HUMAN	100 kDa		0.37	[]	unknown	0	0	0	2	0	0
496	TRUE	Empty	THO compl	THOC5_HUMAN	79 kDa		0.23	[]	unknown	0	0	0	0	0	3
491	TRUE	Empty	WD repeat	WDR36_HUMAN	105 kDa		0.14	[]	unknown	0	0	0	3	0	1
481	TRUE	Empty	Collagen al	COIA1_HUMAN	178 kDa		0.57	[]	unknown	0	2	0	4	0	0
489	TRUE	Empty	RNA polym	PAF1_HUMAN	60 kDa		0.37	[]	unknown	0	0	0	0	0	2
487	TRUE	Empty	pre-rRNA p	SPB1_HUMAN	97 kDa		0.086	[]	unknown	0	0	0	4	0	1
482	TRUE	Empty	Probable A	DDX47_HUMAN	51 kDa		0.086	[]	unknown	0	0	0	3	0	2
488	TRUE	Empty	Splicing fac	U2AF2_HUMAN	54 kDa		0.14	[]	unknown	0	0	0	0	0	4
484	TRUE	Empty	Methyl-Cp	MBD2_HUMAN	43 kDa	TRUE	0.086	[]	unknown	0	0	0	1	0	4
497	TRUE	Empty	Nesprin-2	SYNE2_HUMAN	796 kDa		0.23	[]	unknown	0	0	0	0	0	3
493	TRUE	Empty	Pumilio do	K0020_HUMAN	74 kDa		0.14	[]	unknown	0	0	0	2	0	2
502	TRUE	Empty	Protein kin	PKCB1_HUMAN	132 kDa		0.23	[]	unknown	0	0	0	1	0	2
486	TRUE	Empty	40S riboso	RS28_HUMAN	8 kDa		0.5	[]	unknown	0	1	0	3	0	0
490	TRUE	Empty	WD repeat	WDR82_HUMAN	35 kDa		0.37	[]	unknown	0	0	0	0	0	2
522	TRUE	Empty	Serine/argi	SRSF4_HUMAN	57 kDa	TRUE	0.014	[]	unknown	2	2	3	11	6	12
160.2	TRUE	Empty	ADP/ATP t	ADT3_HUMAN	33 kDa	TRUE	0.4	[]	unknown	2	5	5	5	4	10
39.2	TRUE	Empty	Myosin-10	MYH10_HUMAN	229 kDa	TRUE	0.52	[]	unknown	0	1	4	3	0	6
22.3	TRUE	Empty	Histone H2	H2A2B_HUMAN	14 kDa	TRUE	0.26	[]	unknown	63	51	76	107	38	114
524	TRUE	Empty	Protein S1	S10A7_HUMAN	11 kDa		0.43	[]	unknown	3	0	0	1	1	1
528	TRUE	Empty	Cornifin-A	SPR1A_HUMAN (+1)	10 kDa		0.036	[]	unknown	4	0	1	0	1	0
520	TRUE	Empty	Small nucle	RUXE_HUMAN	11 kDa		0.17	[]	unknown	0	1	0	3	0	3
8.5	TRUE	Empty	Histone H1	H1T_HUMAN	22 kDa	TRUE	0.36	[]	unknown	0	11	17	24	9	19
462.1	TRUE	Empty	REST corep	RCOR1_HUMAN	53 kDa	TRUE	0.032	[]	unknown	0	0	0	1	0	6
519	TRUE	Empty	Kinesin-lik	KI20A_HUMAN	100 kDa		0.086	[]	unknown	0	0	0	3	0	2
527	TRUE	Empty	Serine/thre	PGAM5_HUMAN	32 kDa		0.5	[]	unknown	0	0	1	2	0	1
523	TRUE	Empty	WD repeat	WDR18_HUMAN	47 kDa		0.5	[]	unknown	1	0	0	0	1	2
521	TRUE	Empty	ATP-depen	DHX8_HUMAN	139 kDa	TRUE	0.086	[]	unknown	0	0	0	2	0	3
518	TRUE	Empty	Coiled-coil	CHCH3_HUMAN	26 kDa		0.5	[]	unknown	0	0	1	1	2	0
529	TRUE	Empty	Lethal(3)m	LMBL3_HUMAN	88 kDa		0.14	[]	unknown	0	0	0	0	0	4
526	TRUE	Empty	RNA-bindir	RBM27_HUMAN	119 kDa		0.14	[]	unknown	0	0	0	0	0	4
506	TRUE	Empty	Mitotic che	BUB3_HUMAN	37 kDa		0.25	[]	unknown	0	0	1	2	0	3
512	TRUE	Empty	U3 small n	U3IP2_HUMAN	52 kDa		0.36	[]	unknown	1	0	0	3	1	0
525	TRUE	Empty	Putative 60	R39L5_HUMAN (+1)	6 kDa		0.5	[]	unknown	0	1	0	3	0	0
513	TRUE	Empty	Cleavage a	CPSF7_HUMAN	52 kDa		0.052	[]	unknown	0	0	0	1	0	5
509	TRUE	Empty	Pre-mRNA	FIP1_HUMAN	67 kDa		0.086	[]	unknown	0	0	0	1	0	4
515	TRUE	Empty	EMILIN-1 C	EMIL1_HUMAN	107 kDa		0.059	[]	unknown	0	3	0	0	0	0
517	TRUE	Empty	Golgi appa	GSLG1_HUMAN	135 kDa		0.5	[]	unknown	0	1	0	2	0	1
514	TRUE	Empty	Nuclear po	NUP93_HUMAN	93 kDa		0.5	[]	unknown	1	0	0	3	0	0
510	TRUE	Empty	Pre-mRNA	RBM22_HUMAN	47 kDa		0.052	[]	unknown	0	0	0	3	0	3
516	TRUE	Empty	Integrator	INT3_HUMAN	118 kDa		0.37	[]	unknown	0	0	0	0	0	2
511	TRUE	Empty	Tenascin O	TENA_HUMAN	241 kDa		0.078	[]	unknown	0	4	0	1	0	0
507	TRUE	Empty	Chromoso	CHAP1_HUMAN	89 kDa		0.14	[]	unknown	0	0	0	0	0	4
508	TRUE	Empty	Cold-induc	CIRBP_HUMAN	19 kDa		0.052	[]	unknown	0	0	0	0	0	6
4.2	TRUE	Empty	Histone H2	H2B1J_HUMAN	14 kDa	TRUE	0.22	[]	unknown	51	33	53	72	24	101
93.1	TRUE	Empty	60S acidic	RLA0_HUMAN	34 kDa	TRUE	0.54	[]	unknown	8	6	15	16	11	19
270.2	TRUE	Empty	Hemoglobi	HBA_BOVIN	15 kDa	TRUE	0.012	control hig	unknown	1	8	0	3	0	0
274.1	TRUE	Empty	40S riboso	RS27_HUMAN	9 kDa	TRUE	0.32	[]	unknown	4	1	3	5	2	2
379.1	TRUE	Empty	Homeobox	CUX1_HUMAN	164 kDa	TRUE	0.086	[]	unknown	0	0	0	1	0	4
546	TRUE	Empty	Protein tra	SC61B_HUMAN	10 kDa		0.57	[]	unknown	0	1	1	2	2	0
550	TRUE	Empty	Peptidyl-pr	PPIH_HUMAN	19 kDa		0.14	[]	unknown	0	0	0	2	1	1
555	TRUE	Empty	Cell divisio	CCAR1_HUMAN	133 kDa		0.5	[]	unknown	1	0	0	0	0	3
545	TRUE	Empty	Histone-lys	NSD2_HUMAN	152 kDa		0.5	[]	unknown	1	0	0	0	0	3
543	TRUE	Empty	Calponin-3	CNN3_HUMAN	36 kDa	TRUE	0.14	[]	unknown	0	0	0	0	2	2
544	TRUE	Empty	Cystatin-A	CYTA_HUMAN	11 kDa		0.052	[]	unknown	0	0	0	0	6	0
557	TRUE	Empty	General tra	TF3C2_HUMAN	101 kDa		0.14	[]	unknown	0	0	0	0	0	4
556	TRUE	Empty	Pre-mRNA	CWC22_HUMAN	105 kDa		0.14	[]	unknown	0	0	0	0	0	4
536	TRUE	Empty	Nuclear po	NU205_HUMAN	228 kDa		0.64	[]	unknown	1	1	0	3	0	0
540	TRUE	Empty	Splicing fac	U2AF1_HUMAN	28 kDa		0.64	[]	unknown	0	1	1	0	0	3
542	TRUE	Empty	Zinc finger	ZN516_HUMAN	124 kDa		0.14	[]	unknown	0	0	0	0	0	4
547	TRUE	Empty	Testis-expr	TEX10_HUMAN	106 kDa		0.14	[]	unknown	0	0	0	2	0	2
552	TRUE	Empty	Non-histor	HMGN1_HUMAN	11 kDa		0.5	[]	unknown	2	0	0	2	0	0
548	TRUE	Empty	ATP-citrate	ACLY_HUMAN	121 kDa		0.23	[]	unknown	0	0	0	0	1	2
554	TRUE	Empty	Protein SD	SDA1_HUMAN	80 kDa		0.23	[]	unknown	0	0	0	2	0	1
549	TRUE	Empty	RNA-bindir	RBM28_HUMAN	86 kDa		0.23	[]	unknown	0	0	0	2	0	1
531	TRUE	Empty	C-terminal	CTBP2_HUMAN	49 kDa	TRUE	0.052	[]	unknown	0	0	0	1	0	5
538	TRUE	Empty	KRR1 small	KRR1_HUMAN	44 kDa		0.14	[]	unknown	0	0	0	3	0	1
553	TRUE	Empty	Transitiona	TERA_HUMAN	89 kDa		0.23	[]	unknown	0	0	0	0	1	2

534	TRUE	Empty	MKI67 FHA	MK67I_HUMAN	34 kDa		0.086	[]	unknown	0	0	0	1	0	4
533	TRUE	Empty	Mediator c	MED23_HUMAN	156 kDa		0.086	[]	unknown	0	0	0	0	0	5
532	TRUE	Empty	Lymphoid-	HELLS_HUMAN	97 kDa		0.14	[]	unknown	0	0	0	0	0	4
551	TRUE	Empty	Nuclear po	NU153_HUMAN	154 kDa		0.37	[]	unknown	0	0	0	2	0	0
539	TRUE	Empty	Nuclear po	NUP98_HUMAN	198 kDa		0.14	[]	unknown	0	0	0	4	0	0
535	TRUE	Empty	Nuclear po	NU107_HUMAN	106 kDa		0.23	[]	unknown	0	0	0	3	0	0
537	TRUE	Empty	Polymeras	PDIP3_HUMAN	46 kDa		0.086	[]	unknown	0	0	0	0	0	5
541	TRUE	Empty	Activated F	TCP4_HUMAN	14 kDa		0.14	[]	unknown	0	0	0	0	0	4
5.4	TRUE	Empty	Actin, alph	ACTC_HUMAN (+1)	42 kDa	TRUE	< 0.00010	control hig	unknown	62	97	91	79	68	42
4.3	TRUE	Empty	Histone H2	H2B1A_HUMAN	14 kDa	TRUE	0.07	[]	unknown	41	22	42	45	21	68
152.2	TRUE	Empty	Histone de	HDAC1_HUMAN	55 kDa	TRUE	0.021	[]	unknown	1	1	3	10	0	12
274.2	TRUE	Empty	40S riboso	RS27L_HUMAN	9 kDa	TRUE	0.23	[]	unknown	4	2	3	5	2	2
575	TRUE	Empty	Single-stra	SSBP_HUMAN	17 kDa		0.64	[]	unknown	0	1	1	0	1	2
207.2	TRUE	Empty	Ezrin OS=H	EZRI_HUMAN	69 kDa	TRUE	0.16	[]	unknown	1	1	3	0	0	3
568	TRUE	Empty	Trifunction	ECHB_HUMAN	51 kDa		0.5	[]	unknown	0	2	0	1	0	1
574	TRUE	Empty	Lysine-spe	KDM2A_HUMAN	133 kDa		0.14	[]	unknown	0	0	0	0	0	4
559	TRUE	Empty	Cyclin-dep	CD11A_HUMAN (+1)	91 kDa		0.14	[]	unknown	0	0	0	0	0	4
563	TRUE	Empty	Nuclear ca	NCBP1_HUMAN	92 kDa		0.086	[]	unknown	0	0	0	0	0	5
560	TRUE	Empty	Histone ac	KAT7_HUMAN	71 kDa		0.14	[]	unknown	0	0	0	1	0	3
63.2	TRUE	Empty	Filamin-C (	FLNC_HUMAN	291 kDa	TRUE	0.34	[]	unknown	0	2	0	0	1	0
569	TRUE	Empty	Signal reco	SRP14_HUMAN	15 kDa		0.23	[]	unknown	0	0	0	1	0	2
573	TRUE	Empty	Cleavage s	CSTF1_HUMAN	48 kDa		0.23	[]	unknown	0	0	0	2	0	1
572	TRUE	Empty	Luc7-like p	LC7L3_HUMAN	51 kDa		0.14	[]	unknown	0	0	0	0	0	4
570	TRUE	Empty	Intron-binc	AQR_HUMAN	171 kDa		0.14	[]	unknown	0	0	0	2	0	2
566	TRUE	Empty	Chromodo	CDYL1_HUMAN	66 kDa		0.23	[]	unknown	0	0	0	2	0	1
564	TRUE	Empty	PDZ and LI	PDLI4_HUMAN	35 kDa		0.66	[]	unknown	0	0	1	0	2	0
571	TRUE	Empty	Ribosomal	LAS1L_HUMAN	83 kDa		0.23	[]	unknown	0	0	0	1	0	2
562	TRUE	Empty	THO compl	THOC6_HUMAN	38 kDa		0.14	[]	unknown	0	0	0	1	0	3
565	TRUE	Empty	LIM domai	LIMA1_HUMAN	85 kDa		0.23	[]	unknown	0	0	0	3	0	0
561	TRUE	Empty	Menin OS=	MEN1_HUMAN	68 kDa		0.23	[]	unknown	0	0	0	0	0	3
567	TRUE	Empty	Ribosome	BOP1_HUMAN	84 kDa		0.37	[]	unknown	0	0	0	2	0	0
318.2	TRUE	Empty	Copine-8 C	CPNE8_HUMAN	63 kDa	TRUE	1	[]	unknown	0	0	0	1	0	0
5.5	TRUE	Empty	Actin, cyto	ACTG_HUMAN	42 kDa	TRUE	< 0.00010	control hig	unknown	78	120	117	110	91	71
93.2	TRUE	Empty	60S acidic	RLA0L_HUMAN	34 kDa	TRUE	0.53	[]	unknown	7	6	14	15	10	18
1.3	TRUE	Empty	Desmin OS	DESM_HUMAN	54 kDa	TRUE	0.0019	control hig	unknown	24	28	0	26	0	16
576	TRUE	Empty	Y-box-bind	YBOX3_HUMAN	40 kDa	TRUE	0.19	[]	unknown	0	3	1	2	1	9
586	TRUE	Empty	Importin su	IMB1_HUMAN	97 kDa		0.23	[]	unknown	0	0	0	2	0	1
293.2	TRUE	Empty	Chromobo	CBX1_HUMAN	21 kDa	TRUE	0.39	[]	unknown	0	0	1	1	0	1
292.2	TRUE	Empty	Q3TTY5-DE	Q3TTY5-DECOY	?	TRUE	0.37	[]	unknown	0	0	0	0	2	0
595	TRUE	Empty	Glioma tun	GSCR2_HUMAN	54 kDa		0.23	[]	unknown	0	0	0	2	1	0
582	TRUE	Empty	Methyl-Cp	MBD3_HUMAN	33 kDa	TRUE	0.36	[]	unknown	0	0	1	1	0	3
598	TRUE	Empty	Elongation	EF2_HUMAN	95 kDa	TRUE	0.37	[]	unknown	0	0	3	0	1	3
505.1	TRUE	Empty	(Bos taurus	ENSEMBL:ENSBTAP00	?	TRUE	0.059	[]	unknown	0	3	0	0	0	0
585	TRUE	Empty	Developme	DRG1_HUMAN	41 kDa		0.34	[]	unknown	0	0	2	1	0	0
591	TRUE	Empty	Recombini	SUH_HUMAN	56 kDa		0.23	[]	unknown	0	0	0	2	0	1
588	TRUE	Empty	Peroxidasi	PXDN_HUMAN	165 kDa		0.34	[]	unknown	0	2	0	1	0	0
594	TRUE	Empty	Retinoblas	RBBP5_HUMAN	59 kDa		0.23	[]	unknown	0	0	0	1	0	2
530.1	TRUE	Empty	U1 small n	SNRPA_HUMAN	31 kDa	TRUE	0.14	[]	unknown	0	0	0	0	1	3
592	TRUE	Empty	Mediator c	MED14_HUMAN	161 kDa		0.23	[]	unknown	0	0	0	1	0	2
587	TRUE	Empty	Integrator	INT7_HUMAN	107 kDa		0.37	[]	unknown	0	0	0	0	0	2
590	TRUE	Empty	THO compl	THOC3_HUMAN	39 kDa		0.37	[]	unknown	0	0	0	0	0	2
593	TRUE	Empty	Transcripti	TCRG1_HUMAN	124 kDa		0.37	[]	unknown	0	0	0	0	0	2
583	TRUE	Empty	Pre-mRNA	FL2D_HUMAN	44 kDa		0.37	[]	unknown	0	0	0	0	0	2
597	TRUE	Empty	REST corep	RCOR2_HUMAN	58 kDa	TRUE	0.37	[]	unknown	0	0	0	0	0	2
480.1	TRUE	Empty	Cleavage s	CSTF2_HUMAN	61 kDa	TRUE	0.23	[]	unknown	0	0	0	0	0	3
596	TRUE	Empty	Protein dis	PDIA1_HUMAN	57 kDa		0.37	[]	unknown	0	0	0	2	0	0
589	TRUE	Empty	Plakophilin	PKP1_HUMAN	83 kDa		0.37	[]	unknown	0	0	0	0	2	0
584	TRUE	Empty	Heat shock	HS90B_HUMAN	83 kDa		0.37	[]	unknown	0	0	0	0	0	2
581	TRUE	Empty	Monofunct	C1TM_HUMAN	106 kDa		0.23	[]	unknown	0	0	0	0	0	3
1.4	TRUE	Empty	Neurofilar	NFM_HUMAN	102 kDa	TRUE	0.036	[]	unknown	8	0	0	0	0	7
577	TRUE	Empty	Eukaryotic	IF2B_HUMAN	38 kDa		0.23	[]	unknown	0	0	0	0	0	3
578	TRUE	Empty	Telomeric	TERF2_HUMAN	60 kDa		0.23	[]	unknown	0	0	0	0	0	3
579	TRUE	Empty	Casein kina	KC1A_HUMAN	39 kDa		0.37	[]	unknown	0	0	0	0	0	2
580	TRUE	Empty	Protein arg	ANM1_HUMAN	42 kDa		0.37	[]	unknown	0	0	0	0	0	2
2.2	TRUE	Empty	Epiplakin C	EPIPL_HUMAN	556 kDa	TRUE	0.61	[]	unknown	0	4	0	0	4	0
294.2	TRUE	Empty	Fibrillin-2 (	FBN2_HUMAN	315 kDa	TRUE	1	[]	unknown	0	0	0	0	0	0
89.2	TRUE	Empty	ATP-depen	DX39A_HUMAN	49 kDa	TRUE	0.12	[]	unknown	4	4	8	9	5	23
407.1	TRUE	Empty	Ig gamma-	IGHG1_HUMAN	36 kDa	TRUE	0.43	[]	unknown	2	0	1	0	3	0
79.2	TRUE	Empty	Polypyrimi	PTBP3_HUMAN	60 kDa	TRUE	0.02	[]	unknown	0	0	0	6	0	8
278.2	TRUE	Empty	Bromodor	BRD3_HUMAN	80 kDa	TRUE	0.024	[]	unknown	0	0	1	5	0	8
337.1	TRUE	Empty	Chromodo	CHD9_HUMAN	326 kDa	TRUE	0.086	[]	unknown	0	0	0	0	0	5
228.3	TRUE	Empty	Serine/thr	PP1G_HUMAN	37 kDa	TRUE	0.032	[]	unknown	0	0	0	1	0	6
600	TRUE	Empty	Heat shock	HSP71_HUMAN	70 kDa	TRUE	0.02	[]	unknown	0	0	0	3	0	11
624	TRUE	Empty	Serine/arg	SRSF5_HUMAN	31 kDa	TRUE	0.086	[]	unknown	0	0	0	0	6	8
616	TRUE	Empty	Dynein ligh	DYL1_HUMAN (+1)	10 kDa		0.66	[]	unknown	0	1	0	2	0	0
611	TRUE	Empty	Proteasom	PSA6_HUMAN	27 kDa		0.66	[]	unknown	0	0	1	0	2	0
603	TRUE	Empty	Transcripti	TAF6_HUMAN	73 kDa		0.23	[]	unknown	0	0	0	1	0	2
620	TRUE	Empty	Exosome c	EXOS9_HUMAN	49 kDa		0.23	[]	unknown	0	0	0	1	0	2
618	TRUE	Empty	Nucleolar c	NOC4L_HUMAN	58 kDa		0.23	[]	unknown	0	0	0	2	0	1
621	TRUE	Empty	Vacuolar p	VPS72_HUMAN	41 kDa		0.23	[]	unknown	0	0	0	1	0	2
601	TRUE	Empty	Actin-relat	ARP3_HUMAN	47 kDa		0.23	[]	unknown	0	0	0	1	0	2
614	TRUE	Empty	Splicing fac	SF3B4_HUMAN	44 kDa		0.23	[]	unknown	0	0	0	1	0	2
602	TRUE	Empty	Lysyl oxida	LOXL4_HUMAN	84 kDa		0.15	[]	unknown	0	2	0	0	0	0
17.2	TRUE	Empty	RNA-bindir	RMXL2_HUMAN	43 kDa	TRUE	0.16	[]	unknown	10	0	0	14	0	13
617	TRUE	Empty	Transducin	TBL3_HUMAN	89 kDa		0.37	[]	unknown	0	0	0	2	0	0
610	TRUE	Empty	Lactotransf	TRFL_HUMAN	78 kDa		0.37	[]	unknown	0	0	0	0	2	0
608	TRUE	Empty	DNA-direct	RPB3_HUMAN	31 kDa		0.37	[]	unknown	0	0	0	0	0	2
612	TRUE	Empty	DnaJ homo	DNJC9_HUMAN	30 kDa		0.37	[]	unknown	0	0	0	0	0	2
606	TRUE	Empty	Ubiquitin c	UBP10_HUMAN	87 kDa		0.37	[]	unknown	0	0	0	2	0	0
613	TRUE	Empty	Borealin O	BOREA_HUMAN	31 kDa		0.37	[]	unknown	0	0	0	2	0	0
609	TRUE	Empty	Eukaryotic	IF6_HUMAN	27 kDa		0.37	[]	unknown	0	0	0	2	0	0
604	TRUE	Empty	Leucine-ric	LRC15_HUMAN	64 kDa		0.37	[]	unknown	0	0	0	0	2	0
605	TRUE	Empty	E3 ubiquiti	RING2_HUMAN	38 kDa		0.37	[]	unknown	0	0	0	0	0	2
607	TRUE	Empty	Gremlin-1	GREM1_HUMAN	21 kDa		0.37	[]	unknown	0	0	0	0	2	0

615	TRUE	Empty	Ribosome	BMS1_HUMAN	146 kDa		0.37	[]	unknown	0	0	0	2	0	0
619	TRUE	Empty	General tra	TF2H3_HUMAN	34 kDa		0.37	[]	unknown	0	0	0	0	0	2
622	TRUE	Empty	Rac GTPase	RGAP1_HUMAN	71 kDa		0.37	[]	unknown	0	0	0	2	0	0
623	TRUE	Empty	Heterogen	HNRPF_HUMAN	46 kDa	TRUE	0.23	[]	unknown	0	0	0	0	0	3
264.3	TRUE	Empty	Poly(rC)-bi	PCBP4_HUMAN	41 kDa	TRUE	1	[]	unknown	0	0	0	0	0	1
52.2	TRUE	Empty	Ninein OS=	NIN_HUMAN	243 kDa	TRUE	0.61	[]	unknown	0	0	0	2	0	0
211.2	TRUE	Empty	Host cell fa	HCFC2_HUMAN	87 kDa	TRUE	0.61	[]	unknown	0	0	0	0	0	2
39.3	TRUE	Empty	Myosin-14	MYH14_HUMAN	228 kDa	TRUE	1	[]	unknown	0	0	0	2	0	0
337.2	TRUE	Empty	Chromodo	CHD6_HUMAN	305 kDa	TRUE	0.37	[]	unknown	0	0	0	0	0	3
4.4	TRUE	Empty	Histone H2	H2B1M_HUMAN	14 kDa	TRUE	0.083	[]	unknown	67	49	65	97	32	119
4.5	TRUE	Empty	Histone H2	H2B1D_HUMAN	14 kDa	TRUE	0.066	[]	unknown	67	49	65	97	32	119
10.2	TRUE	Empty	Ubiquitin-6	RL40_HUMAN	15 kDa	TRUE	0.009	control low	unknown	34	39	45	62	27	49
17.3	TRUE	Empty	RNA bindir	RMXL1_HUMAN	42 kDa	TRUE	0.21	[]	unknown	16	9	7	21	3	35
4.6	TRUE	Empty	Putative hi	H2B2C_HUMAN	21 kDa	TRUE	0.24	[]	unknown	5	2	8	8	2	10
407.2	TRUE	Empty	Ig gamma-	IGHG2_HUMAN (+2)	36 kDa	TRUE	0.57	[]	unknown	1	0	1	0	4	0
558.1	TRUE	Empty	Cofilin-1 O	COF1_HUMAN	19 kDa	TRUE	0.5	[]	unknown	0	0	1	1	2	0
643	TRUE	Empty	Spermatid	STRBP_HUMAN	74 kDa	TRUE	0.23	[]	unknown	0	0	0	0	0	3
241.2	TRUE	Empty	ATPase fan	ATD3C_HUMAN	46 kDa	TRUE	0.62	[]	unknown	0	2	1	2	1	5
337.3	TRUE	Empty	Chromodo	CHD7_HUMAN	336 kDa	TRUE	0.052	[]	unknown	0	0	0	0	0	6
599.1	TRUE	Empty	Transcripti	TEAD1_HUMAN	48 kDa	TRUE	0.37	[]	unknown	0	0	0	0	0	2
48.2	TRUE	Empty	40S riboso	RS4Y1_HUMAN	29 kDa	TRUE	0.61	[]	unknown	0	0	6	0	4	4
634	TRUE	Empty	SWI/SNF-r	SMRD3_HUMAN	55 kDa	TRUE	0.37	[]	unknown	0	0	0	0	0	2
625	TRUE	Empty	Cleavage s	CSTF3_HUMAN	83 kDa		0.37	[]	unknown	0	0	0	0	0	2
626	TRUE	Empty	Interferon-	E2AK2_HUMAN	62 kDa		0.37	[]	unknown	0	0	0	0	0	2
627	TRUE	Empty	RNA-bindi	RBMS1_HUMAN	45 kDa		0.37	[]	unknown	0	0	0	0	0	2
628	TRUE	Empty	Atherin OS	SAMD1_HUMAN	56 kDa		0.37	[]	unknown	0	0	0	0	0	2
629	TRUE	Empty	TOX high n	TOX4_HUMAN	66 kDa		0.37	[]	unknown	0	0	0	0	0	2
630	TRUE	Empty	Deoxynucl	TDIF2_HUMAN	84 kDa		0.37	[]	unknown	0	0	0	2	0	0
631	TRUE	Empty	Regulation	RPR1B_HUMAN	37 kDa		0.37	[]	unknown	0	0	0	0	0	2
632	TRUE	Empty	DNA prima	PRI1_HUMAN	50 kDa		0.37	[]	unknown	0	0	0	0	0	2
633	TRUE	Empty	Coiled-coil	CC137_HUMAN	33 kDa		0.37	[]	unknown	0	0	0	0	0	2
635	TRUE	Empty	Actin-relat	ARPC2_HUMAN	34 kDa		0.37	[]	unknown	0	0	0	0	2	0
636	TRUE	Empty	WD repeat	WDR76_HUMAN	70 kDa		0.37	[]	unknown	0	0	0	0	0	2
637	TRUE	Empty	Regulator	RENT1_HUMAN	124 kDa		0.37	[]	unknown	0	0	0	0	0	2
638	TRUE	Empty	Protein SS	SSXT_HUMAN	46 kDa		0.37	[]	unknown	0	0	0	0	0	2
639	TRUE	Empty	PCI domair	PCID2_HUMAN	46 kDa		0.37	[]	unknown	0	0	0	0	0	2
640	TRUE	Empty	Putative RI	LUC7L_HUMAN	44 kDa	TRUE	0.37	[]	unknown	0	0	0	0	0	2
641	TRUE	Empty	Eukaryotic	EIF3M_HUMAN	43 kDa		0.15	[]	unknown	0	0	2	0	0	0
642	TRUE	Empty	Putative RI	LC7L2_HUMAN	47 kDa	TRUE	0.37	[]	unknown	0	0	0	0	0	2
337.4	TRUE	Empty	Chromodo	CHD8_HUMAN	291 kDa	TRUE	0.23	[]	unknown	0	0	0	0	0	4
39.4	TRUE	Empty	Myosin-11	MYH11_HUMAN	227 kDa	TRUE	0.57	[]	unknown	0	2	0	2	0	3
1.5	TRUE	Empty	Peripherin	PERI_HUMAN	54 kDa	TRUE	0.032	[]	unknown	0	0	0	0	13	0
31.3	TRUE	Empty	Chromodo	CHD5_HUMAN	223 kDa	TRUE	< 0.00010	control low	unknown	0	0	0	0	0	24
64.2	TRUE	Empty	Probable g	SMCA2_HUMAN	181 kDa	TRUE	0.0027	control low	unknown	0	0	0	0	0	25
80.2	TRUE	Empty	DNA topoi	TOP1M_HUMAN	70 kDa	TRUE	1	[]	unknown	0	0	0	0	0	6
278.3	TRUE	Empty	Bromodom	BRDT_HUMAN	108 kDa	TRUE	1	[]	unknown	0	0	0	1	0	0
4.7	TRUE	Empty	Histone H2	H2B3B_HUMAN	14 kDa	TRUE	0.39	[]	unknown	50	31	50	69	23	97
6.2	TRUE	Empty	Histone H3	H31T_HUMAN	16 kDa	TRUE	0.0017	control hig	unknown	25	16	25	24	6	25
420.1	TRUE	Empty	Putative eu	IF2GL_HUMAN	51 kDa	TRUE	0.62	[]	unknown	0	0	3	1	1	3
219.2	TRUE	Empty	Histone H2	H2AV_HUMAN	14 kDa	TRUE	0.14	[]	unknown	65	53	80	111	38	115
420.2	TRUE	Empty	Eukaryotic	IF2G_HUMAN	51 kDa	TRUE	0.62	[]	unknown	0	0	3	1	1	3
94.2	TRUE	Empty	60S riboso	RL26L_HUMAN	17 kDa	TRUE	0.0034	control hig	unknown	10	6	9	9	4	0
558.2	TRUE	Empty	Cofilin-2 O	COF2_HUMAN	19 kDa	TRUE	0.37	[]	unknown	0	0	0	1	1	0
353.2	TRUE	Empty	TATA-bindi	RBP56_HUMAN	62 kDa	TRUE	0.37	[]	unknown	0	0	0	0	0	5
530.2	TRUE	Empty	U2 small n	RU2B_HUMAN	25 kDa	TRUE	0.61	[]	unknown	0	0	0	0	0	2
160.3	TRUE	Empty	ADP/ATP t	ADT1_HUMAN	33 kDa	TRUE	0.41	[]	unknown	0	0	3	0	3	7
462.2	TRUE	Empty	REST corep	RCOR3_HUMAN	56 kDa	TRUE	0.61	[]	unknown	0	0	0	0	0	1
318.3	TRUE	Empty	Copine-7 C	CPNE7_HUMAN	70 kDa	TRUE	0.61	[]	unknown	0	0	0	0	0	2
480.2	TRUE	Empty	Cleavage s	CSTFT_HUMAN	64 kDa	TRUE	0.37	[]	unknown	0	0	0	0	0	2
380.2	TRUE	Empty	Tropomyos	TPM3_HUMAN	33 kDa	TRUE	0.37	[]	unknown	0	0	0	0	2	0
599.2	TRUE	Empty	Transcripti	TEAD2_HUMAN	49 kDa	TRUE	0.61	[]	unknown	0	0	0	0	0	1
324.2	TRUE	Empty	Alpha-actir	ACTN4_HUMAN	105 kDa	TRUE	0.61	[]	unknown	0	0	0	0	2	0
505.2	TRUE	Empty	Fibulin-1 O	FBLN1_HUMAN	77 kDa	TRUE	1	[]	unknown	0	0	0	0	0	0
17.4	TRUE	Empty	RNA-bindir	RMXL3_HUMAN	115 kDa	TRUE	0.61	[]	unknown	0	0	0	4	0	0
379.2	TRUE	Empty	Protein CA	CASP_HUMAN	77 kDa	TRUE	1	[]	unknown	0	0	0	0	0	0
160.4	TRUE	Empty	ADP/ATP t	ADT4_HUMAN	35 kDa	TRUE	0.39	[]	unknown	0	2	0	0	0	0
34.2	TRUE	Empty	TREMBL:Q	Q28194	?	TRUE	0.0013	control hig	unknown	18	0	0	0	0	0
56.3	TRUE	Empty	Polyadenyl	PABP3_HUMAN	70 kDa	TRUE	1	[]	unknown	0	0	0	0	0	0
324.3	TRUE	Empty	Alpha-actir	ACTN2_HUMAN	104 kDa	TRUE	1	[]	unknown	0	0	0	0	0	0
22.4	TRUE	Empty	Histone H2	H2AX_HUMAN	15 kDa	TRUE	1	[]	unknown	0	0	0	0	0	0
159.2	TRUE	Empty	Lamina-ass	LAP2A_HUMAN	75 kDa	TRUE	1	[]	unknown	0	0	0	0	0	0
207.3	TRUE	Empty	Radixin OS	RADI_HUMAN	69 kDa	TRUE	0.39	[]	unknown	0	1	0	0	0	0
67.2	TRUE	Empty	60S riboso	RL3L_HUMAN	46 kDa	TRUE	1	[]	unknown	0	0	0	0	0	0

END OF FILE
