# Supplementary material for: Progeria‐based vascular model identifies networks associated with cardiovascular aging and disease
Source: Aging Cell. 2024 Apr 4;23(7):e14150. doi: 10.1111/acel.14150 (PMC11258467; doi:10.1111/acel.14150)
Supplement: Supplementary file 5 — Data S1. [file ACEL-23-e14150-s003.docx]

Table S1.

A table showing aniPOND-mass spectrometry spectral counts of proteins at replication fork of HGPS and control VMSCs at passage 7 and passage 14.
